# Genome-Wide Comparative Analysis of R2R3 MYB Gene Family in *Populus* and *Salix* and Identification of Male Flower Bud Development-Related Genes

**DOI:** 10.3389/fpls.2021.721558

**Published:** 2021-09-14

**Authors:** Fangwei Zhou, Yingnan Chen, Huaitong Wu, Tongming Yin

**Affiliations:** Key Laboratory for Tree Breeding and Germplasm Improvement, Southern Modern Forestry Collaborative Innovation Center, College of Forestry, Nanjing Forestry University, Nanjing, China

**Keywords:** *Populus*, *Salix*, R2R3 MYB transcription factors, GAMYB, pollen

## Abstract

The MYB transcription factor (TF) family is one of the largest plant transcription factor gene family playing vital roles in plant growth and development, including defense, cell differentiation, secondary metabolism, and responses to biotic and abiotic stresses. As a model tree species of woody plants, in recent years, the identification and functional prediction of certain MYB family members in the poplar genome have been reported. However, to date, the characterization of the gene family in the genome of the poplar’s sister species willow has not been done, nor are the differences and similarities between the poplar and willow genomes understood. In this study, we conducted the first genome-wide investigation of the R2R3 MYB subfamily in the willow, identifying 216 R2R3 MYB gene members, and combined with the poplar R2R3 MYB genes, performed the first comparative analysis of R2R3 MYB genes between the poplar and willow. We identified 81 and 86 pairs of R2R3 MYB paralogs in the poplar and willow, respectively. There were 17 pairs of tandem repeat genes in the willow, indicating active duplication of willow R2R3 MYB genes. A further 166 pairs of poplar and willow orthologs were identified by collinear and synonymous analysis. The findings support the duplication of R2R3 MYB genes in the ancestral species, with most of the R2R3 MYB genes being retained during the evolutionary process. The phylogenetic trees of the R2R3 MYB genes of 10 different species were drawn. The functions of the poplar and willow R2R3 MYB genes were predicted using reported functional groupings and clustering by OrthoFinder. Identified 5 subgroups in general expanded in woody species, three subgroups were predicted to be related to lignin synthesis, and we further speculate that the other two subgroups also play a role in wood formation. We analyzed the expression patterns of the GAMYB gene of subgroup 18 (S18) related to pollen development in the male flower buds of poplar and willow at different developmental stages by qRT-PCR. The results showed that the GAMYB gene was specifically expressed in the male flower bud from pollen formation to maturity, and that the expression first increased and then decreased. Both the specificity of tissue expression specificity and conservation indicated that GAMYB played an important role in pollen development in both poplar and willow and was an ideal candidate gene for the analysis of male flower development-related functions of the two species.

## Introduction

The initiation of transcription in eukaryotes is highly complex and often requires the assistance of a variety of protein factors (Shi et al., 2017). Transcription factors and RNA polymerase II form a transcription initiation complex to jointly participate in the transcription initiation process and control gene expression (Kamenova et al., 2019). MYB superfamily is one of the largest groupings of transcription factors and is found in both animals and plants. These proteins usually contain a highly conserved MYB domain at the N-terminus (Frampton et al., 1989; Stracke et al., 2001; Dubos et al., 2010; Jiang et al., 2011). Recently, it has been found that some MYB proteins also contain a MYB domain at the C-terminus (Linger and Price, 2009). The MYB domain is 51–52 amino acids in length and contains four imperfect amino acid repeats (R) each forming a helix-turn-helix (HTH) structure with three tryptophan residues acting as a hydrophobic core in each helix. These HTH structures are responsible for the specific binding of the MYB proteins to DNA (Cheng et al., 2016). According to the number of amino acids and tryptophan conservation, the MYB domain is divided into three subtypes, R1, R2, and R3 (Jin and Martin, 1999; Dubos et al., 2010). Based on the number of R repeats within the domain, MYB superfamily members are divided into four main groups: 1R-MYB (MYB-related proteins), 2R-MYB (R2R3), 3R-MYB (R1R2R3), and 4R-MYB (R1R2R2R1/2-MYB) (Frampton et al., 1989; Li et al., 2019). The R2R3 MYB subfamily is the most abundant type in plants (Jin and Martin, 1999; Millard et al., 2019b).

With the release of the whole-genome sequences of increasing numbers of plant species, R2R3 MYB proteins have been identified and analyzed in numerous species based, especially, on the structures of the conserved MYB domain. During plant evolution, the number of R2R3 MYB family members has expanded considerably due to a large number of gene duplication events. Studies have found that some gymnosperms have relatively few members of the R2R3 MYB family, for example, *Ginkgo biloba* (68 members) (Yang et al., 2021). And whole-genome analysis of dicotyledonous plants shows that most species have between 70 and 200 family members (Du et al., 2015), for example, *Arabidopsis thaliana* (126 members) (Stracke et al., 2001), *Zea mays* (157 members) (Du et al., 2012a), *Oryza sativa* (102 members) (Chen et al., 2006), *Vitis vinifera* (134 members) (Wong et al., 2016), *Camellia sinensis* (122 members) (Chen et al., 2021), and *Populus trichocarpa* (192 members) (Chai et al., 2014). Each pedigree continued to expand after the divergence. In addition to these expansion and diversification events, the tandem duplication of subgroups has also been predicted. Therefore, some species have more than 200 gene family members, for example, *Glycine max* (244 members) (Du et al., 2012b) and *Gossypium raimondii* (205 members) (He et al., 2016), while *Brassica rapa* ssp. *pekinensis* has 256 members, the largest known plant R2R3 MYB gene family (Wang et al., 2015). The differences in the numbers of R2R3 MYB genes between species, in addition to gene amplification, may also be caused by environmental adaptation, indicating specificity after separation from the last common ancestor (Chang and Duda, 2012).

Research on the functions of plant R2R3 MYBs indicates that the functions of these transcription factors are diverse (Yang et al., 2012). They can participate in plant growth and development in multiple ways (Ambawat et al., 2013). In *A. thaliana*, *AtMYB16* and *AtMYB106* are mainly involved in regulating epidermal hair differentiation (Baumann et al., 2007; Jakoby et al., 2008), while *AtMYB58* and *AtMYB63* can specifically activate lignin biosynthesis (Noda et al., 2015; Wang F. P. et al., 2018). R2R3 MYBs can also regulate the synthesis of flavonoids and other secondary metabolites by regulating the expression of genes related to plant secondary metabolism (Aharoni et al., 2001; Jiang and Rao, 2020). For example, *AtMYB75* (*PAP1*), *AtMYB90* (*PAP2*), *AtMYB113*, and *AtMYB114* in *A. thaliana* can regulate the accumulation of anthocyanins (Borevitz et al., 2000; Bhargava et al., 2010; Ma and Constabel, 2019). *AtMYB123* (*TT2*) function in proanthrocyanidin synthesis in plants (Nesi et al., 2001). R2R3 MYBs also play important roles in abiotic and biotic stress responses (Baldoni et al., 2015), such as *AtMYB15*, *AtMYB30*, *AtMYB60*, and *AtMYB96* from *A. thaliana* (Seo and Park, 2010; Cominelli et al., 2015). Additionally, R2R3 MYBs can also control cell morphogenesis and regulate the growth and development of flowers, fruits, and seeds (Lv et al., 2017). For example, *AtMYB33*, *AtMYB65*, and *AtMYB101* in *A. thaliana* play important roles in flower development, especially in pollen development (Gocal et al., 2001; Li et al., 2016). Genes regulating the development of stamens have also been found in other species with the particular gene identified as GAMYB (Aya et al., 2011). For example, the *HvGAMYB* gene of *Hordeum vulgare* plays an important role in the development of flowers, especially in the development of pollen (Murray et al., 2003), mutation of the rice GAMYB gene results in abnormal stamen development, shortened filaments, and sterile pollen (Ko et al., 2021), while the GAMYB gene *PtrMYB012* in the poplar heterologous transformation of *A. thaliana* also leads to male sterility (Zhang et al., 2020).

Poplar and willow belong to the Salicaceae family, which is widely distributed in the northern hemisphere (Wei et al., 2020). Both species adapt easily to different ecological environments, grow rapidly, and reproduce without difficulty. They are important tree species for timber forest, shelter forest, roadside trees, and greening and have important economic, ecological, and social value (Zhang J. et al., 2017). However, when the male tree reaches sexual maturity, it produces a great deal of pollen, which causes allergic reactions in some people (Futamura et al., 2000; Costache et al., 2021). Therefore, studying the reproductive mechanism of poplar and willow is valuable for the improvement of forest trees. At present, both the poplar and willow whole-genome sequences have been sequenced and are publicly available (Tuskan et al., 2006; Dai et al., 2014). Although R2R3 MYB genes have been identified and analyzed in the poplar (Chai et al., 2014), this is not the case for the willow. To date, most of our understanding of the function of the R2R3 MYB genes in plants is derived from the analysis of the model plant *A. thaliana*. In the poplar and willow, only a few R2R3 MYB genes have been cloned and functionally verified (Fang et al., 2020; Tang et al., 2020; Wang et al., 2020; Liu C. et al., 2021). As these genes play important roles in multiple growth and developmental pathways, it is urgent to characterize their roles in the Salicaceae, to identify and classify the genes, and thus to provide a reference for subsequent functional verification of the R2R3 MYB gene family. In this study, the R2R3 MYB gene family of willow was comprehensively identified and analyzed for the first time. By comparing the willow genes with the poplar R2R3 MYB genes, the specific poplar and willow family members after differentiation of the species were identified. Further, the function of the R2R3 MYB gene family members was comprehensively predicted, and the expression patterns of family members related to stamen development were verified, providing a reference for the functional verification of the R2R3 MYB gene family members in the Salicaceae.

## Materials and Methods

### Genome-Wide Identification and Sequence Analysis of *Populus, Salix*, and *A. thaliana* R2R3 MYB Genes

The consensus R2R3 MYB DNA-binding domain (containing 106 amino acid residues) (Ogata et al., 1994) sequence was used to identify homologous genes in the *Populus trichocarpa* genome database (version 3.1)^1^ and *Salix purpurea* genome database (version 1.0)^2^ (Zhou et al., 2018, Zhou R. et al., 2020; Costache et al., 2021). We also conducted further BLAST searches in the protein database using an *E*-value cut-off of 1e-005 (Chen et al., 2021). The Hidden Markov Model (HMM) profile for the R2R3 MYB binding domain was downloaded from Pfam^3^ to verify the data, and protein domains were manually identified using both Pfam and Smart^4^ (Letunic and Bork, 2018; Potter et al., 2018; El-Gebali et al., 2019), ensuring that the putative R2R3 MYB genes contained two MYB DNA-binding domains. Finally, 192 *Populus trichocarpa* and 216 *Salix purpurea* R2R3 MYB proteins were identified. Using these protein sequences, we obtained the corresponding cDNA and genomic sequences, chromosomal locations, intron distribution patterns, phases, and intron/exon boundaries at the Joint Genome Institute (JGI).^5^ In addition, 126 *A. thaliana* R2R3 MYB protein sequences were downloaded from the *A. thaliana* Information Resource (TAIR)^6^ (Dubos et al., 2010). The R2R3 MYB protein sequences of seven other species were downloaded, including *Physcomitrella patens* (50 members) (Du et al., 2013), *Chlamydomonas reinhardtii* (10 members) (Du et al., 2013), *Vitis vinifera* L. (134 members) (Wong et al., 2016), *Zea mays* (157 members) (Du et al., 2012a), *Brassica napus* (249 members) (Hajiebrahimi et al., 2017), *Malus domestica* (228 members) (Cao et al., 2013), and *Ginkgo biloba* L. (69 members) (Yang et al., 2021).

### Chromosomal Distribution of R2R3 MYB Genes

The chromosomal location information of the R2R3 MYB gene family was extracted using the annotation files of the poplar and willow genomes, and the length of each chromosome was obtained from the NCBI website^7^; the gene positions were drafted to chromosomes by using MapChart software (Voorrips, 2002). As described previously, homologous chromosomal segments generated by genome-wide duplication events were identified. Paralogous genes and tandem duplicated genes were identified based on criteria in the poplar and willow genome annotations. Paralogous genes were determined by aligning and phylogenetically analyzing full-length R2R3-MYB proteins (He et al., 2016; Zhou W. et al., 2020). Genes with a distribution on the chromosome in the range of 100 kb and sequences with a sequence similarity greater than 70% were considered to be tandem duplicates (Chai et al., 2014). To further analyze gene duplication events, the paralogous and orthologous gene pairs were aligned using ClustalX2.1 and analyzed using KaKs_calculator software to estimate their synonymous (ks) of evolution (Thompson et al., 1994; Zhang et al., 2006).

### Phylogenetic Analysis and Functional Prediction of R2R3 MYB Genes

ClustalX 2.1 was used to perform multiple sequence alignments on the full-length protein sequences of poplar and willow R2R3 MYBs under the default parameters, following which the alignments were manually adjusted before constructing the phylogenetic tree (Thompson et al., 1994). The maximum likelihood (ML) and neighbor-joining (NJ) phylogenetic trees were constructed using MAGE X64 using Poisson correction, pairwise deletion, and bootstrap analysis with 1,000 replicates (Kumar et al., 2018). The R2R3 MYB genes were then classified according to their phylogenetic relationship with the corresponding *A. thaliana* R2R3 MYB genes (Dubos et al., 2010).

All R2R3 MYB protein sequences from poplar, willow, *A. thaliana, P. patens*, *C. reinhardtii*, grapevine, maize, *B. napus*, apple, and ginkgo were clustered using OrthoFinder v2 with a cut-off *e*-value of 1 × 10^–3^ (Emms and Kelly, 2019). The multiple sequence alignments and phylogenetic trees were constructed with MEGA X64 (Kumar et al., 2018) using the full-length sequences of identified R2R3 MYB proteins for each orthogroup. The phylogenetic tree was visualized using EVOLVIEW^8^ and a folding phylogenetic tree, comprised of 10 species was constructed (Subramanian et al., 2019). Certain biological functions of the R2R3 MYB proteins were predicted based on their homologs in the phylogenetic tree.

### Gene Structure Analysis and Protein Motif Identification

The R2R3 MYB gene sequences and gene annotation files for poplar and willow can be found in the JGI genome portal. The gene structure data used were selected from a genome sequence annotation file. General Feature Format (GFF) files were used to extract the exact positions of all introns and exons in each predicted gene model. The number and length of the introns and exons were then calculated. Exon length (Exon 1–Exon 5) values were analyzed using Box plot in matplotlib for *Populus, Salix*, and *A. thaliana*. The exon-intron organization was determined by the online program Gene Structure Display Server (GSDS)^9^ comparing the predicted coding sequences to their corresponding full-length sequences (Hu et al., 2015).

To investigate conserved motifs in the R2R3 MYB protein sequences in more detail, we used the online program MEME^10^ to identify conserved motifs shared among the R2R3 MYB proteins (Bailey et al., 2009). The following parameter settings were used: any for the number of repetitions, the distribution of the motifs, zero or one per sequence, with up to 20 motifs in total. The minimum width of the motif was set at 2 amino acids and the maximum width at 250 amino acids (to identify long R2R3 domains). Other options used the default values. Only hits with e-values < 1e^–20^ were retained for further analysis. The orders of the ID numbers of the R2R3 MYB genes in the exon-intron figures and motif distribution figures are the same as those in the phylogenetic trees. TBtools software graphically represented the motif distribution of R2R3 MYB genes (Chen et al., 2020).

### Duplications and Syntenic Analysis of R2R3 MYB Genes

To determine the correspondence between poplar and willow R2R3 MYB genes, we analyzed the synteny and collinearity of these genes between the genomes by using MCScan software (Wang et al., 2012). The software is an algorithm that scans multiple genome sequences or sub-genome sequences to identify putative homologous chromosomal regions and then arranges these regions using genes as anchors (R2R3 MYB transcription factors in our case). We obtained information on the detailed chromosomal location of each R2R3 MYB gene from the genome annotation document. Based on this, the synteny and collinearity relationships of the R2R3 MYB genes of poplar and willow were visualized using Circos software (Krzywinski et al., 2009).

### Plant Material

Root, stem, and leaf samples were collected from 6-month-old cultured poplar and willow plantlets grown under natural conditions in the greenhouse of Nanjing Forestry University (32°N, 118°W) under natural conditions. The male and female flower buds of the poplar were collected from the *Populus deltoides* planted on the campus of Nanjing Forestry University, and the male and female flower buds of the willow were collected from the *Salix suchowensis* planted in the Baima Base of Nanjing Forestry University. The flower buds were collected from September 2019 to March 2020. The male and female flower buds of both poplar and willow consist of six different developmental stages: T1 (flower bud differentiation stage), T2 (flower bud dormancy stage), and T3-T6 (pollen grain production from formation to maturity). All the collected samples were immediately frozen in liquid nitrogen and stored at –80°C for RNA extraction.

### Gene Expression Analysis

Total RNA was extracted from the different poplar and willow tissues using the RNAprep Pure Polysaccharide Polyphenol Plant Total RNA Extraction Kit (TIANGEN, NanJing, China) following the manufacturer’s instructions. The RNA quality was assessed by 1.0% agarose gel electrophoresis. The first-strand cDNA was synthesized using 1 μg total RNA and One-Step gDNA Removal and cDNA Synthesis SuperMix (TIANGEN, NanJing, China). The reverse-transcribed cDNA was diluted with ddH_2_O at a ratio of 1:10 and used as a template for qRT PCR. Primers were designed using Primer 5.0 based on the laboratory’s existing poplar and willow CDS database, and primer specificity was determined by BLAST. *PtUBQ* (Potri.015G013600.1) and *DnaJ* (SapurV1A.0212s0110) were used as internal control genes for poplar and willow, respectively (Gutierrez et al., 2008; Zhang Y. X. et al., 2017). The total reaction mixture of each PCR experiment comprised of forward and reverse primers (4 pM each), 2 μl of cDNA (diluted 10 times), 10 μl of PowerUp^TM^ SYBR^TM^ Green Master Mix (Applied Biosystems, United States), and RNase-free water to 20 μl. The reaction was conducted in the 7,500 Fast Real-Time PCR System (Applied Biosystems, United States). The qRT-PCR reaction conditions were: 95°C for 3 min, followed by 40 cycles of 95°C for 15 s, 60°C for 15 s, and 72°C for 30 s. At the end of each experiment, the default parameters were used to conduct melting curve analysis for 60 s at 55–95°C in 0.3°C increments. The expression of the genes was analyzed according to the 2^–ΔΔCt^ method (Livak and Schmittgen, 2001).

### Prediction of *Cis*-Acting Elements in the Promoter Region of GAMYB Genes in Poplar and Willow

The 2,000-bp sequence upstream of the transcription starting point of the poplar and willow GAMYB genes was identified from the databases and the PlantCARE online website^11^ was used to predict the sequences and *cis*-acting elements of the promoter regions (Lescot et al., 2002).

## Results

### Identification of *Populus and Salix* R2R3 MYB Transcription Factors

According to the known sequence characteristics of the R2R3 MYB domain, 192 and 216 R2R3 MYB transcription factors were identified in the recently updated poplar genome database (version 3.1) and willow genome database (version 1.0), respectively (Tuskan et al., 2006; Zhou et al., 2018, Zhou R. et al., 2020). The identification of the poplar R2R3 MYB genes was consistent with the 192 R2R3-MYB members reported by Wilkins et al. (2009), and two fewer members than Chai et al. (2014). In the reference version (i.e., V3.1), the amino acid sequence of *PtrMYB076* does not have the typical R2R3 MYB domain and belongs to 1R-MYB, therefore it was not defined as R2R3 MYB, while *PtrMYB235* has no homolog gene in version 3.1. Thus, we only have 192 R2R3 MYB members. The R2R3 MYB domain sequence in poplar and willow was found to be analogous to that of other plant species, containing approximately 106 amino acids with only a few deletions or insertions. The domain contained characteristic amino acid residues with evenly distributed and highly conserved Trp (W) residues (Chen et al., 2021). Based on the HMM results, we manually verified the accuracy of the sequences and deleted incomplete R2R3 MYB domain sequences and R-R-type groups not listed as R2R3 MYB but classified as Atypical-MYB (Jiang and Rao, 2020). The accession numbers of the R2R3 MYB genes from poplar and willow are listed in Supplementary Tables 1, 2, respectively, together with information of their positions on the chromosomes. The amino acid sequences and CDS sequences of the poplar proteins are listed in Supplementary Sequences 2, 3, respectively, and the corresponding willow sequences in Supplementary Sequences 4, 5. The accession numbers and corresponding amino acid sequences of 126 R2R3 MYB genes in *A. thaliana* are listed in Supplementary Sequence 5 (Dubos et al., 2010).

### Chromosomal Distribution and Identification of Paralogous Gene Pairs in *Populus and Salix* R2R3 MYB Transcription Factors

**FIGURE 1:**
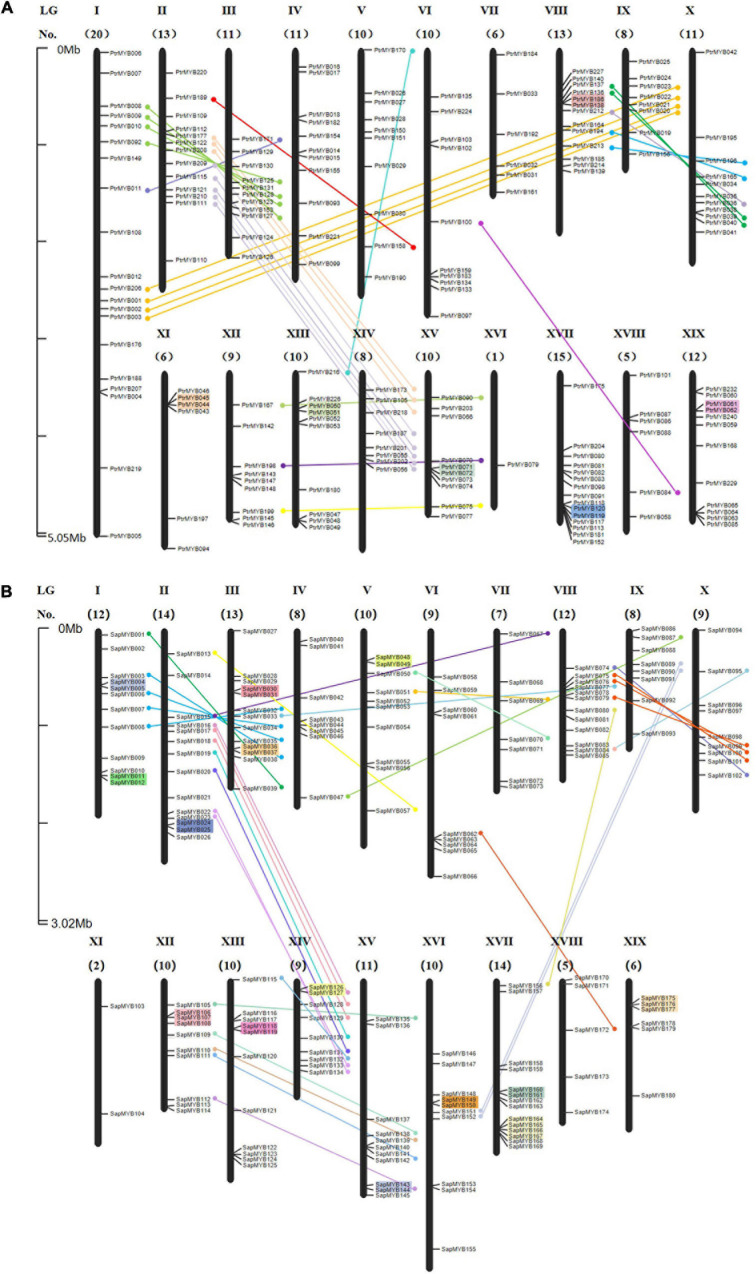
Chromosomal locations for R2R3 MYB genes of poplar and willow. **(A)** Poplar and **(B)** willow R2R3 MYB genes were mapped to their corresponding chromosomes based on the published genome information of the poplar and willow. Chromosome numbers are displayed above each chromosome. The numbers below indicate the number of R2R3 MYB genes on each chromosome. The scale is in megabases (Mb). Each gene is named according to the order of its corresponding location on the chromosome. Paralogous genes are indicated by lines, genes contained in the segmental duplicated homologous blocks are connected by lines of the same color, and tandemly duplicated genes are indicated by colored backgrounds.

To date, although there have been reports on expansion events in the poplar R2R3 MYB family (Chai et al., 2014), the situation in the willow is still unclear. To determine the relationship between genetic divergence and gene duplication within the R2R3 MYB family in the Salicaceae and using the genome annotation information, 189 poplar R2R3 MYB genes (Figure 1A) and 180 willow R2R3 MYB genes (Figure 1B) were mapped to the corresponding 19 chromosomes, respectively. According to the order of their corresponding locations on the chromosome, the willow R2R3 MYB genes were named *SpuMYB001* to *SpuMYB180.* 36 R2R3 MYB genes in the willow that could not be mapped to a chromosome, remaining only on unassembled scaffold fragments, were named *SpuMYB181* to *SpuMYB216.* The poplar has only three R2R3 MYB genes that are not mapped to chromosomes, and the naming of the poplar R2R3 MYB genes refers to published articles (Wilkins et al., 2009; Chai et al., 2014). The number of willow R2R3 MYB genes that were not mapped to chromosomes was significantly higher than that in the poplar, which may be the reason for the incomplete assembly of the willow genome. We found that the genes mapped on the chromosomes were unevenly distributed. The distribution numbers of the R2R3 MYB genes in the poplar and willow were compared, showing that there were significant differences in the number of genes on chromosomes I and XVI with the poplar having 20 R2R3 MYB genes on chromosome I while there were only 12 on chromosome I in the willow. In contrast, only one R2R3 MYB gene was identified on chromosome XVI in poplar while the willow had 10 genes on chromosome XVI. These observations suggest that chromosomes I and XVI undergo chromosomal rearrangement (Dai et al., 2014).

The presence of paralogous genes is the result of gene family expansion, which is mainly produced by segmental duplication and tandem duplication events. One or more genes located on the same chromosome within 100 kb and having high sequence similarities (> 70%) with their counterparts were considered to be tandem duplicates, denoted as the “T” event (Liu et al., 2014; Wang M. et al., 2018). A segmental duplication event is produced by a genome-wide replication event (Magwanga et al., 2018). The gene duplication seen in the poplar indicates a single genome-wide event. We refer to this duplication event as a “salicoid” duplication event, that is, the “P” event, which occurred around 58 Mya (Tuskan et al., 2006). About 6 Mya after the “salicoid” duplication event, two major inter-chromosomal rearrangements and several minor intra-chromosomal rearrangements occurred. After this chromosomal rearrangement event, called the “β” event, the ancestors of the modern willow appeared, indicating that willow and poplar were differentiated from a common paleotetraploid ancestor (Hou et al., 2016). The genome duplication in poplar and willow is very recent, occurring around 8–13 Mya, defined as the “Pt-α” events and “Sp-α” events, respectively (Sterck et al., 2005; Berlin et al., 2010). The Ks value reflects the generation time of the homologous gene to some extent and is often used as the basis for assessing the gene duplication events (Cui et al., 2006; Hou et al., 2015).

**FIGURE 2:**
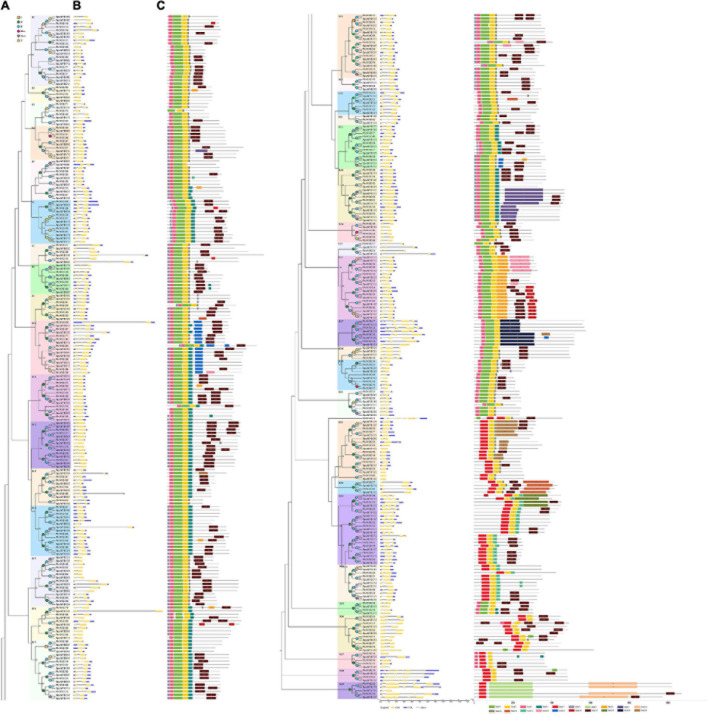
Phylogenetic relationships, gene structure, and motif composition of poplar and willow R2R3 MYB genes. **(A)** The phylogenetic tree was constructed with MAGE X64 using the Neighbor-Joining (NJ) method, with 1,000 bootstrap replicates based on multiple alignments of the full-length amino acid sequences of 192 R2R3 MYB genes from poplar and 216 R2R3 MYB genes from willow; this tree shows 39 subgroups (S1-S39) with high bootstrap values marked with colored backgrounds. Bootstrap values below 50 are not shown in the phylogenetic tree. Only one protein was not assigned to any subgroup. **(B)** GSDS was used to analyze the exon-intron structures of the R2R3 MYB genes. Exons and introns are indicated by yellow boxes and single black lines, respectively. Untranslated upstream and downstream regions are indicated by dark-blue bars at the ends of the sequence. The length of each R2R3 MYB gene can be estimated using the bottom scale. **(C)** Schematic representation of conserved motifs in 39 subgroups of poplar and willow R2R3 MYB proteins elucidated using MEME. Each motif is represented by a number in colored boxes. Black lines represent non-conserved sequences. The size of each motif can be estimated using the scale at the bottom. Motif sequences are listed in Supplementary Table 8. The numbers of conserved motifs in poplar and willow are shown in Supplementary Figure 3. The colored squares (duplication events) and circles (triplication events) placed on nodes represent gene lineage expansion(s) that can be associated with particular ancient polyploid events: γ, P, β, Pt-α, Sp-α. Positions are in Mb.

In this study, paralogous gene pairs were identified to determine the different types of duplication responsible for the R2R3 MYB family expansion in poplar and willow and the reasons for the differences in the numbers of R2R3 MYB genes. Using whole-genome analysis of gene duplications, we identified 81 pairs of paralogous genes in the poplar R2R3 MYB family (Supplementary Table 3), while 86 pairs of paralogous genes were identified in the willow (Supplementary Table 4). There were 70 pairs of homologous genes observed in the poplar, generated by the “salicoid” duplication event in the Salicaceae around 58 Mya. In addition, the recent poplar genome replication event i.e., Pt-α produced 6 pairs of tandem repeat genes and 3 pairs of paralogous genes (Figure 2A). In addition, two additional pairs of homologous genes were generated in the γ triplication event shared by the poplar, willow, and *A. thaliana*. The rate of evolution of genes in the willow genome was about twice that of poplar genome (Hou, 2016). There were four pairs of paralogous genes in the willow that were produced in theγtriplication event and 42 pairs generated in the “salicoid” duplication event. Surprisingly, 17 pairs of tandem repeat genes were identified in the willow, indicating that the willow R2R3 MYB family has been actively replicated. The amino acid sequences of *SpuMYB126* and *SpuMYB127* and *SpuMYB160* and *SpuMYB161* are the same; their corresponding cDNA and DNA sequences are also very similar (100 and 99.62%, 100 and 99.9%, respectively). At the same time, three (*SpuMYB175*, *SpuMYB176*, *SpuMYB177*) tandem repeats and four (*SpuMYB164*, *SpuMYB165*, *SpuMYB166*, *SpuMYB167*) tandem repeats were identified. Their amino acid sequences are highly similar, indicating recent replication events and providing strong evidence that active gene replication has contributed significantly to the expansion of the willow R2R3 MYB gene family. After genome duplication, antagonism eliminates most of the duplicate genes, so there are some genes produced by dispersed duplication. Different replication events appear to have led to the complexity of the R2R3 MYB genes in the willow genome (Dai et al., 2014; Hou et al., 2015). It also provides a theoretical basis suggesting that the willow has undergone more environmental adaptation than the poplar and has evolved more varieties. poplar contains 5 groups with about 40 species. Compared with poplar, willow has a wide variety of species, divided into 33–38 groups, and comprising about 520 species (Skvortsov, 1999; Hou, 2016).

### Synteny Analysis of R2R3 MYB Transcription Factors and Identification of Orthologous Gene Pairs

**FIGURE 3:**
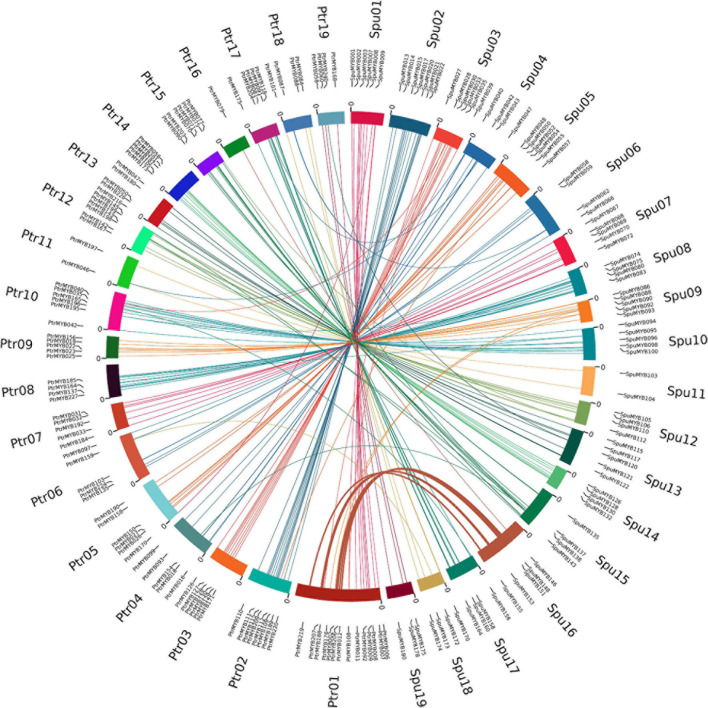
Duplication event analysis of R2R3 MYB genes and comparative synteny analysis between poplar and willow. Circos diagram showing gene positions of 166 orthologous gene pairs on poplar and willow chromosomes, Ptr01-Ptr18 represents 18 poplar chromosomes and Spu01-Spu18 represents 18 willow chromosomes. Orthologous gene pairs are located on a chromosome and connected with a line of the same color as the corresponding chromosome. The size of the chromosome is consistent with the size of the actual pseudochromosome. Positions are in Mb.

Genome-wide replication events in plants have been considered as a mechanism for diversification and adaptation to the environment. Although we found that the R2R3 MYB gene family of the willow had undergone more active replication than the poplar and that there were large phenotypic changes during their evolution, the two species also share many common characteristics, including the same chromosome number of 2*n* = 38 and the common “Salicoid” genomic duplication that occurred around 58 Mya, resulting in high macroscopic homology, and they also retained similar characteristics in terms of growth, reproduction and other developmental processes (Tuskan et al., 2006; Dai et al., 2014). To further analyze the diversity and evolutionary conservation of members of the R2R3 MYB gene family between the poplar and willow, we used OrthoFinder V2 analysis, combined with phylogeny, gene structures, and sequence similarities to identify the orthologous genes between the two species The results showed that most of the R2R3 MYB genes were preserved during the evolution of the poplar and willow. We identified 166 pairs of R2R3 MYB orthologous genes between the poplar and willow. In addition to 117 pairs of one-to-one orthologs, the genomes also contain 45 pairs of one-to-many and 4 pairs of many-to-many co-orthologous genes (Supplementary Table 5), indicating that these genes have expanded to varying degrees. Among them, 158 pairs of orthologous genes were generated during the β event of the fission or fusion of chromosomes in the ancestral genome 6 Mya (Figure 2A). There was also high bootstrap support (≥ 90%) with short branch lengths at the ends of the branches, indicative of high homology and recent co-evolutionary origins (Figure 3). Comparison of the R2R3 MYB genes from poplar and willow revealed that genes with orthologous relationships tended to be clustered together in the phylogenetic tree in contrast to the paralogous genes, indicating that amplifications in the R2R3 MYB gene family were more likely to have occurred in the ancestral species.

During evolution, large segmental duplications and small-scale tandem duplications have been the two main mechanisms for generating new genes. We found that the orthologous R2R3 MYB genes on the poplar chromosomes II–XV and XVII–XIX had similar distributions on the corresponding willow chromosomes, suggesting that they originated from the same tetraploid ancestor. However, no orthologous genes were found on the lower parts of chromosome 1 in both and willow although seven pairs of orthologous genes were detected on chromosome XVI in the willow. This chromosomal distribution of poplar and willow orthologous genes is consistent with previous reports that have described major inter-chromosomal rearrangements and chromosome fission and fusion involving chromosomes I and XVI between the two genomes (Dai et al., 2014; Hou et al., 2016). In addition, we have also identified orthologous genes on other corresponding chromosomes of the poplar and willow; although there are not as many genes as between chromosomes I and XVI, this confirms the conclusion reached by Hou et al. (2019) that, in addition to the large degree of recombination between the two important chromosomes in the poplar and willow, subtle changes between corresponding chromosomes may have also occurred (Hou et al., 2019). This provides a reference for the study of R2R3 MYB differentiation mechanisms after genome-wide replication events in the Salicaceae.

### Phylogeny, Conserved Gene Structures, and Protein Motif Analysis of *Populus and Salix* R2R3 MYB Transcription Factors

Members of the R2R3 MYB gene family in the poplar and willow were subdivided into 39 subgroups based on clades with at least 50% bootstrap support and named S1-S39 (Figure 2A). In our phylogenetic analysis, only the poplar gene *PtrMYB130* did not fit into any subgroup. In addition, the tree (Supplementary Figure 1) topology using maximum likelihood (ML) analysis was essentially the same as the NJ tree (Figure 2A), indicating that the two phylogenetic trees are in good agreement. To analyze the similarity and diversity of the gene structures within the subgroups, we used the GSDS tool to draw the exon/intron schematic structures of 408 R2R3 MYB genes in poplar and willow (Figure 2B). Usually, there is at least one intron in the R2R3 MYB DNA-binding domain, but in our analysis, we found that 5.7% (11/192) and 4.2% (9/216) of poplar and willow R2R3 MYB genes, respectively, lacked introns. In addition, the most common R2R3 MYB intron-exon structure, seen in 73% (140/192) of poplar and 72% (156/216) of willow genes, was a typical splicing of 3 exons and 2 introns. Some R2R3 MYB genes were found to have 2 or 4 exons. However, a total of 5 R2R3 MYB sequences (*PtrMYB131*, *PtrMYB213*, *SpuMYB082*, *PtrMYB196*, and *SpuMYB096*) possessed more than 6 introns. Among them, *PtrMYB213* and *SpuMYB082*, *PtrMYB196*, and *SpuMYB096* were identified as orthologous genes. They formed a separate subgroup (S38) containing a complex exon-intron structure. Although there are some differences in their intron-exon structure, it is worth noting that the results of the phylogenetic analysis of the poplar and willow R2R3 MYB family indicated that genes in the same subgroups usually contained very similar exon-intron patterns, a pattern that appeared to be conserved. For example, the R2R3 MYB genes in subgroup 4 (S4) had 3 exons and 2 introns, those in subgroup 34 (S34) had 2 exons and 1 intron, while the genes with the most exons were all found in subgroup 38 (S38). The analysis of the exon-intron structures of the R2R3 MYB genes supports our phylogenetic analysis. Our data indicate that there were some subgroups (i.e., S7, S10, S13, S14, S16, and S33) of R2R3 MYB with a specific exon-intron structure in which the introns were significantly longer. We speculate that the introns in the R2R3 MYB domain may play an important role in the evolution of the R2R3 MYB gene family through unknown mechanisms. Simultaneously, we found that of the 166 orthologs identified, most gene pairs had the same exon-intron pattern and were highly conserved. The R2R3 MYB genes are not only highly conserved in their exon-intron structures but are also conserved in length. Exons 1 and 2 usually encode almost the entire R2R3 MYB DNA-binding domain, making the first two exons almost the same size in plant species. Generally, exons 1 and 2 are very similar in length (exon 1,132 bp; exon 2,129 bp) and are also highly conserved (exon 1, 34% in poplar, 38% in willow; exon 2, 61% in poplar, 62% in willow). Exon 3 has the greatest diversity in sequence length (poplar, 30–1,131 bp; willow, 33–1,131 bp) (Supplementary Figure 2). We found that the exon lengths of the R2R3 MYB family members in poplar and willow are consistent with the average exon lengths in *A. thaliana* and grapes (Supplementary Figure 2; Matus et al., 2008). The restricted lengths of exons 1 and 2 in R2R3 MYB genes explain the high degree of conservation in the R2R3 MYB domain seen during plant evolution, suggesting that these conservative features play key roles in specific subgroup functions. Exons 3–12 encode the last region of the R3 repeat and the C-terminal region of the R2R3 MYB protein. The length and sequence differences in the exons can lead to different functional motifs and domains, which can explain functional divergence between R2R3 MYB homologs within and between plant species.

The C-terminal region next to the MYB domain in R2R3 MYB proteins usually has large sequence variations, although the domains in each protein subgroup contain functionally important motifs that are less conserved than those in the MYB domain. To analyze the conserved motifs in R2R3 MYB proteins, a total of 20 conserved motifs in the poplar and willow R2R3 MYB proteins were identified using the online MEME tool (Figure 2C). Comparing the distributions of 20 motifs between the poplar and willow, it was found that the number of motifs was similar between the two species and the motifs did not differ significantly in composition. The lengths of these 20 motifs varied between 8 and 250 amino acids, and the number of motifs in each R2R3 MYB sequence varied between 3 and 8. Most R2R3 MYB proteins had motifs 1, 2, 3, 6, 11, and 17 (Supplementary Figure 3), 1, 2, and 3 constituting the conserved R2 and R3 domains of “W-(X19)-W- (X19)–W -…-F/I-(X18)-W-(X18)-W-“. Motif 6 was also identified as the MYB domain, and the other motif functions are unknown. However, while motifs 1, 2, and 3 directly followed the MYB domain in the sequence and were particularly conserved in most subgroups, most motifs were selectively distributed between specific clades of the phylogenetic tree, with each R2R3 MYB subgroup having a common motif. For example, in subgroup 15 (S15), all R2R3 MYB proteins contained motifs 1, 2, 3, 6, 11, and 17, and in subgroup 12 (S12), all the proteins contained motifs 1, 2, 3, 4, 6, and 17. However, certain motifs appear to be unique to certain subgroups, for example, motif 19 was found in subgroup 27 (S27), while motifs 7 and 10 were only identified in subgroup 39 (S39). Some of the genes present in the same group did encode proteins that differed in their shared motifs. For example, *PtrMYB072* in subgroup 3 (S3) lacked motif 3, *SpuMYB211* in subgroup 16 (S16) lacked motif 6, *PtrMYB012* in subgroup 27 (S27) had an additional motif 20, and *SpuMYB148* and *PtrMYB168* in subgroup 14 (S14) had additional motifs 15 and 8, respectively. Generally, most R2R3 MYB genes clustered in the same subgroup showed common motif characteristics, implying that members of the same subgroup have similar functions. Subgroup 39 (S39) was located at the far end of the phylogenetic tree, and most of its members (4/5) had a very long C-terminal region. MEME search results found two unexpectedly large, conserved motifs (motifs 7 and 10) in the C-terminal region of this subgroup, and a large, conserved motif (motif 20) was found in subgroup 27 (S27). It is possible that this may indicate the presence of specific functions in this R2R3 MYB subgroup, however, the biological functions of these proteins require further verification.

### Putative Functions of *Populus and Salix* R2R3 MYB Transcription Factors

**FIGURE 4:**
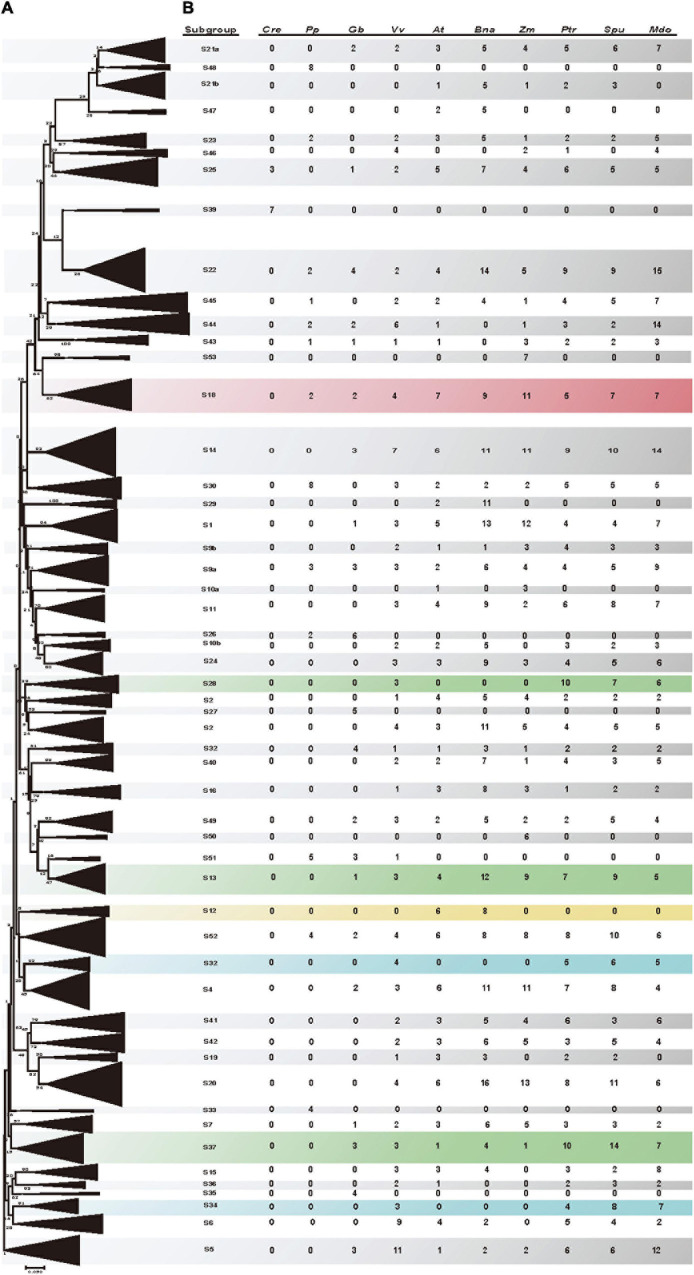
Phylogenetic tree of poplar, willow, *A. thaliana*, *P. patens*, *C. reinhardtii*, grapevine, maize, *B. napus*, apple, and ginkgo R2R3 MYB proteins. **(A)** Neighbor-joining phylogenetic tree (Supporting sequences 4) and Maximum likelihood (ML) phylogenetic tree (Supporting sequences 5) constructed using 1,431 amino acid sequences (Supporting sequences 5) including all of the R2R3-MYB proteins. Folding triangles represent 53 subgroups of the R2R3 MYB protein cluster, consistent with OrthoFinder clustering orthogroup, and prediction of the function of the subgroups, each triangle represents a R2R3-MYB subgroup. Supplementary Figure 4 shows an unfolded evolutionary tree with full classification of members. **(B)** Subgroup names are included next to each clade together with a short name to simplify nomenclature. The number of genes of each species for each subgroup is also included. Subgroups in general expanded in woody species and have been confirmed to be related to lignin synthesis are highlighted in green, and subgroups in general expanded in woody species and predicted to be related to wood development are highlighted in blue. The specific subgroups of Cruciferae are shown in yellow, and the subgroups related to stamen development are shown in red.

Amino acid changes in motifs and domains, as well as sequence length diversity are sources of functional differences. To examine the evolutionary relationships among the identified poplar and willow R2R3 MYBs and R2R3 MYBs from other species, the R2R3 MYB genes of 10 species (poplar, willow, *A. thaliana*, *P. patens*, *C. reinhardtii*, grapevine, maize, *B. napus*, apple, and ginkgo) were analyzed. According to the orthogroups identified by Orthofinder V2 and with reference to previous research on the R2R3 MYB gene family in different species (Stracke et al., 2001), the R2R3 MYB genes were subdivided into 53 subgroups (Figure 4). Since R2R3 MYB proteins in the same subgroup usually control the same molecular regulatory pathway, our results will provide support for the functional prediction of these poplar and willow R2R3 MYB genes. To predict the biological functions of poplar and willow R2R3 MYB, we referred to gene functions that have been fully verified in *A. thaliana* as well as gene functions that have been reported in other species, resulting in the functional annotation of 30 subgroups. For example, members of subgroup 5 (S5) and subgroup 6 (S6) are related to anthocyanin biosynthesis (He et al., 2021), subgroup 1 (S1) members participate in responses to biotic and abiotic stress (Froidure et al., 2010), while subgroup 9a (S9a) members are associated with trichome development (Wu et al., 2018; Zhou et al., 2021), while members of subgroup 37 (S37) and subgroup 46 (S46) all play key roles in lignin biosynthesis (Tohge et al., 2005; Zhou et al., 2009).

Although the number of genes contained in each subgroup was similar between the poplar and willow, we nevertheless identified several evolutionarily different subgroups. For example, the number of willow genes in subgroup 34 (S34) was double that of the poplar, indicating that after willow differentiation, the willow genes in this subgroup replicated more actively in this subgroup than in poplar, including two pairs of tandem repeat genes (*SpuMYB011* and *SpuMYB012*, *SpuMYB030* and *SpuMYB031*). Remarkably, several clades (S13, S28, S32, S34, and S37) of R2R3 MYB genes have undergone different degrees of expansion in woody plants, especially in the poplar and willow. It may be that many genes evolved differently after differentiation from the common ancestor, including some lineage specific gene duplication and gene loss events (Soler et al., 2015). Among them, subgroups 13, 28, and 37 (S13, S28, and S37), which are related to xylem development, have significantly expanded. It was found that the R2R3 MYB genes of subgroup 28 (S28) were highly expressed in vessels and fiber during wood formation, which could regulate the biosynthesis of lignin, cellulose, and xylose (McCarthy et al., 2010). Although the members of the subgroup 32 (S32) and subgroup 34 (S34) have not yet undergone functional verification, the grape, poplar, willow, and *A. thaliana* all evolved from the same ancient hexaploid ancestor. Both subgroups in the grape have R2R3 MYB members. In contrast, there were no R2R3 MYB members in *A. thaliana*, but more genes are replicated in the poplar and willow, so we speculate that the genes of subgroup 32 (S32) and subgroup 34 (S34) likely have similar functions and participate in wood formation. This further suggests that the genes of these subgroups may have specialized roles that were either acquired in poplar and willow or lost in *A. thaliana* after the divergence from the last common ancestor, and they are good candidates for further functional and phylogenetic analysis. In contrast, we also found that certain clades showed a bias toward *A. thaliana* species in the phylogenetic trees. For example, while subgroup 12 (S12) included six *A. thaliana* R2R3 MYB proteins and eight *B. napus* R2R3 MYB proteins, it did not include any poplar or willow genes, nor genes from other species. Recently, it has been reported that the members of this subgroup *AtMYB34*, *AtMYB51*, and *AtMYB122* are involved in the biosynthesis of glucosinolates (Frerigmann and Gigolashvili, 2014). This hypothesis was based on the understanding that glucosinolate compounds are Cruciferae plant-specific (Perez et al., 2021). Although the evolutionary links of all the subgroups are unclear, this study provides the groundwork for future functional studies of poplar and willow R2R3 MYB genes.

### Identification of R2R3 MYB Candidate Genes Related to Stamen Development in Poplar and Willow

Both poplar and willow are dioecious. The male plants are known to produce pollen, which is an important allergen. Therefore, we chose the GAMYB gene to understand the genes’ potential regulatory role in the development of male flowers. We used qRT-PCR technology to analyze the expression patterns of this subgroup of genes in six different developmental stages of male and female flower buds, leaf buds, stems and leaves of both poplar and willow.

**FIGURE 5:**
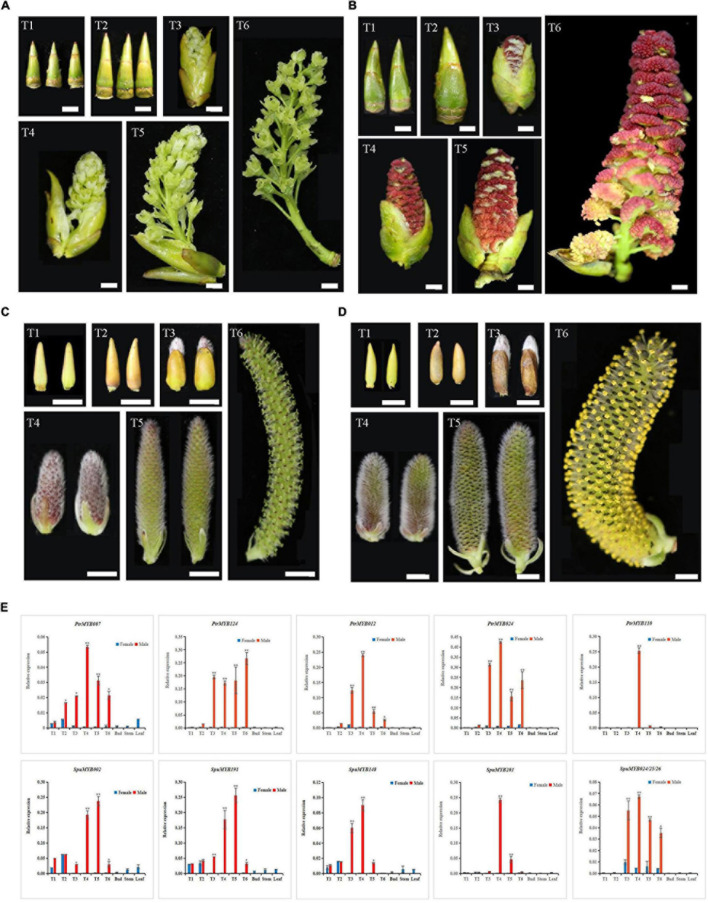
Determination of the expression profile of GAMYB genes in male and female flower buds by qRT-PCR. **(A)** The morphology of female flower buds of poplar at developmental stages T1-T6 as defined in the text. **(B)** The morphology of male flower buds of poplar at developmental stages T1-T6 as defined in the text. **(C)** The morphology of female flower buds of willow in the developmental stages T1-T6 as defined in the text. **(D)** The morphology of female flower buds of willow in the developmental stages T1-T6 as defined in the text. **(E)** The bar graph shows the results of the qRT-PCR analysis of *GAMYB* genes in different tissues and organs. The displayed value shown is the mean ± SD of three biological replicates (*P < 0.05; **P < 0.01, two-sided Student’s *t*-test). Bar = 0.5 mm.

The results showed that the expression levels of 12 poplar and willow R2R3 MYB genes the subgroup 18 (S18) differed in different organs and tissues (Figure 5). The vast majority of these R2R3 MYB genes had relatively low expression levels in different developmental stages of female flower buds, flower bud differentiation (T1) and dormancy (T2) stages of male flower buds as well as in leaf bud, stem, and leaf. From pollen formation to maturity (T3-T6), the expression levels first increased and then decreased, with similar expression patterns in both poplar and willow. *PtrMYB124* had a high expression level during the entire pollen development period, with its highest expression during the pollen maturity stage. The sequence similarities between *SpuMYB024*, *SpuMYB025*, and *SpuMYB026* were as high as 98.91%, and it was difficult to design specific primers to distinguish them. Therefore, we designed universal primers for these three genes to analyze their expression patterns and found that, compared with other GAMYB genes, these three genes were not only expressed in male flowers but were also detected in female flowers, although their expression in the latter was very low. It is speculated that these genes may have acquired different functions during evolution and thus may have a wider range of functions. The tissue specificities and high similarities of GAMYB genes expression in poplar and willow indicate functional conservation in members of this subgroup. Combined with the functional analysis of GAMYB genes in other species. This indicates that the GAMYB genes play an important role in the development of poplar and willow pollen and is an ideal candidate gene for the functional analysis of male flower development in poplar and willow.

### *Cis*-Element Identification in the Promoter Regions of GAMYB Genes

Cis-acting regulatory elements play an important role in the overall regulation of gene expression. Evidence indicates that genes with similar expression patterns may contain the same regulatory elements in their promoters. The GAMYB gene also plays an important role in the development of flowers, especially the development of anthers. To further understand the possible regulatory mechanism of GAMYB in the development of male poplar and willow flower buds, the PlantCARE database was used to identify the cis-acting regulatory elements (CREs) at 2,000 bp upstream of these genes, and a total of 1543 CREs were identified (Supplementary Table 6). It was found that all poplar and willow GAMYB promoter sequences contained TATA-box and CAAT-box core elements. Most genes also contain multiple types of MYB-related elements such as MBS, MYB, and MYC (Supplementary Figure 6). GAMYB is a gibberellin-dependent α-amylase transcription positive regulator, which can specifically bind to the GA response element in the promoter region of the α-amylase gene and other GA-regulated hydrolase genes (Murray et al., 2003). The TATC-box and P-box are cis-acting elements involved in the gibberellin reaction (Chen et al., 2021). We found that 9 GAMYB gene promoters contained one or more of these two elements, of which *SpuMYB002* contained 4 P-box and 1 TATC-box elements, the *PtrMYB043* promoter contained 3 P-box, *PtrMYB096* and *SpuMYB191* both contained 2 P-box, while *PtrMYB002*, *PtrMYB010*, *SpuMYB024*, *SpuMYB025*, and *SpuMYB026* each contained one P-box element. This indicates that these genes are combined with GA response elements to participate in the GA signaling pathway and regulate the development of flower buds by regulating the expression of genes in this pathway. It has been found that other GAMYB gene promoters contain other hormone response-related elements. For example, *SpuMYB201* contains the auxin response element TGA-element, and *PtrMYB033* contains the cis-acting element ABRE that participates in the abscisic acid response. MYB transcription factors usually form MBW complexes with bHLH and WD40 (Liu Y. F. et al., 2021). The bHLH transcription factor mainly regulates downstream target genes by recognizing the E-box (CANNTG). Among them, *PtrMYB012*, *PtrMYB110*, *SpuMYB024*, *SpuMYB025*, *SpuMYB026*, *SpuMYB201*, and *SpuMYB002* all contain G-box cis-elements, indicating that these genes may be regulated by bHLH transcription factors.

## Discussion

The “salicoid” duplication event in the Salicaceae family occurred around 58 Mya (Tuskan et al., 2006; Dai et al., 2014). About 6 Mya after the whole-genome duplication, chromosome breakage and fusion occurred in the paleotetraploid genome, and the genome doubled again (Tuskan et al., 2006; Dai et al., 2014). During the subsequent evolution, chromosome 1 of the poplar tree had a centromere break, and the lower part of chromosome I fused with the telomere of chromosome XVI to form chromosome I of willow, the upper part of which evolved to form the willow chromosome XVI (Hou et al., 2015, 2016). This chromosomal rearrangement event marked the appearance of the ancestors of the modern willow, indicating that the willow resulted from the poplar chromosome breakage and fusion. During the subsequent evolution after this differentiation, both poplar and willow not only retained the common Salicaceae characteristics but also evolved their own unique characters to adapt to the environment. Therefore, the comparative analysis of the important gene families of the poplar and willow is helpful to identify the retention and loss of key gene family members after the differentiation of the two species. The MYB transcription factor superfamily is one of the largest gene families in plants, and R2R3 MYB transcription factors participate in plant cell differentiation, growth, metabolism, and various stress responses (Millard et al., 2019a; Chang et al., 2020). Through comparative analysis of the R2R3 MYB transcription factor family in the poplar and willow, it was found that they have both retained most of the gene members of the ancestral species. It was found the family expanded faster in the willow than in the poplar through tandem replication or chromosome segment duplication. The mean substitution rates of willow and poplar are 1.09 × 10^–9^ and 0.67 × 10^–9^/site/year, respectively (Dai et al., 2014). The mean substitution rate in the willow was significantly higher than that in the poplar, which indicated that the evolutionary rate in the willow was faster than that in the poplar (Dai et al., 2014). Therefore, we infer that the rapid expansion of the R2R3 MYB gene family during the rapid evolutionary process in the willow may have resulted in more sub-functionalization, allowing the newly generated gene family members to be preserved and promoting better adaptation to a complex environment.

We combined our analysis with the poplar, willow, *A. thaliana*, *Physcomitrella patens*, *Chlamydomonas reinhardtii*, grapevine, maize, *Brassica napus*, apple, and ginkgo R2R3 MYB gene families for a comprehensive classification and evolutionary analysis. Phylogenetic analysis divided the R2R3 MYB transcription factors in the 10 species into 53 subgroups. Due to the conservation of the R2R3 MYB genes, genes with similar or identical functions were classified into the same subgroup, providing a reliable basis for studying the functions of related genes in this gene family. Thus, the R2R3 MYB members of subgroups (S13, S28, S31) have been confirmed to be involved in lignin biosynthesis in other species, and these subgroups have expanded to different degrees in the poplar and willow. Therefore, it is speculated that the R2R3 MYB genes of the poplar and willow clustered in the same subgroup are also related to wood development (Zhou et al., 2009). The *A. thaliana* R2R3 MYB genes of subgroup 6 (S6), *AtMYB75* (*PAP1*), *AtMYB90* (*PAP2*), *AtMYB113*, and *AtMYB114*, are the main transcription factors regulating anthocyanin biosynthesis (Borevitz et al., 2000; Bhargava et al., 2010). As it is speculated that there are functional similarities between poplar and willow and their R2R3 MYB members on the same branch, these genes may play important roles in regulating anthocyanin biosynthesis in poplar and willow. In subgroup 1 (S1), *AtMYB30*, *AtMYB60*, and *AtMYB96* can activate transcription factors that respond to stress and play important roles in plant growth under adverse conditions (Raffaele and Rivas, 2013; Rusconi et al., 2013). It is speculated that poplar and willow members of this subgroup with close genetic distances have similar functions and can respond to stress conditions. In addition, *AtMYB33* and *AtMYB65* of subgroup 18 (S18) participate in the GA signal transduction pathway to control flowering, resulting in the name of GAMYB for these genes (Gao et al., 2020). This gene subgroup has been found to have similar regulatory roles in *H. vulgare*, *A. thaliana*, *O. sativa*, and *C. sativus*. GAMYB genes have been shown to play important roles in the development of flowers, especially the development of anthers (Liu et al., 2010; Zhang et al., 2014; Abraham et al., 2016). Therefore, the study of the flowering mechanism in the poplar and willow is of significant value.

This study analyzed the expression patterns of the GAMYB gene in subgroup 18 (S18) at different developmental stages of male and female flower buds of poplar and willow. It is speculated that they play a role in the process of male flower formation. We further predicted the cis-acting elements in their promoter regions and found that most of the GAMYB gene promoter regions contain cis-acting elements related to hormone response, with 75% of the GAMYB gene promoters containing the cis-acting elements TATC-box and P-box related to the GA signaling pathway. Similarly, the GAMYB gene promoter sequence also contained numerous other cis-acting elements related to light response, plant growth and development, regulation, and the stress response. This implies that while members of this subgroup may have different biological functions and abilities to participate in signaling pathways, most are involved in GA signaling to regulate flowering pathways. We next intend to verify these gene functions through transgene studies and protein interactions, amongst other methods, which will lay a foundation for the understanding of the role of R2R3 MYB transcription factors in regulating male flower development and provide directions for breeding new non-pollen-producing varieties of male poplar and willow plants.

## Data Availability Statement

The datasets presented in this study can be found in online repositories. The names of the repository/repositories and accession number(s) can be found in the article/Supplementary Material.

## Author Contributions

TY and HW designed the research and revised the manuscript. FZ conducted the experiments and data analysis, and drafted the manuscript. YC participated in data analysis. All authors reviewed and approved the final manuscript.

## Conflict of Interest

The authors declare that the research was conducted in the absence of any commercial or financial relationships that could be construed as a potential conflict of interest.

## Publisher’s Note

All claims expressed in this article are solely those of the authors and do not necessarily represent those of their affiliated organizations, or those of the publisher, the editors and the reviewers. Any product that may be evaluated in this article, or claim that may be made by its manufacturer, is not guaranteed or endorsed by the publisher.

## Footnotes

1
https://phytozome.jgi.doe.gov/pz/portal.html#!info?alias=Org_Ptrichocarpa_er

2
https://phytozome.jgi.doe.gov/pz/portal.html#!info?alias=Org_Spurpurea

3
http://pfam.xfam.org/

4
http://smart.embl.de/smart/set_mode.cgi?NORMAL=10

5
https://phytozome.jgi.doe.gov/pz/portal.html#!search?show=KEYWORD&method=Org_Tcacao

6
https://www.arabidopsis.org/

7
https://www.ncbi.nlm.nih.gov/

8
https://evolgenius.info//evolview-v2/#login

9
http://gsds.cbi.pku.edu.cn/index.php

10
http://alternate.meme-suite.org/tools/meme

11
http://bioinformatics.psb.ugent.be/webtools/plantcare/html/

## Supplementary Material

Supplementary Figure 1 Maximum likelihood phylogenetic tree of poplar and willow R2R3 MYB genes.

Supplementary Figure 2 Distribution of exon lengths in poplar and willow R2R3 MYB genes.

Supplementary Figure 3 Distribution and numbers of different motifs in the poplar and willow R2R3 MYB genes.

Supplementary Figure 4 Neighbor-joining phylogenetic tree of poplar, willow, *A. thaliana*, *P. patens*, *C. reinhardtii*, grapevine, maize, *B. napus*, apple, and ginkgo R2R3 MYB genes.

Supplementary Figure 5 Maximum likelihood phylogenetic tree of poplar, willow, *A. thaliana*, *P. patens*, *C. reinhardtii*, grapevine, maize, *B. napus*, apple, and ginkgo R2R3 MYB genes.

Supplementary Figure 6 Cis-element analysis of the promoter regions of GAMYB genes.

Supplementary Table 1 List of 192 identified poplar R2R3 MYB transcription factors.

Supplementary Table 2 List of 216 identified willow R2R3 MYB transcription factors.

Supplementary Table 3 List of poplar R2R3 MYB paralogous gene pairs.

Supplementary Table 4 List of willow R2R3 MYB paralogous gene pairs.

Supplementary Table 5 List the poplar and willow R2R3 MYB orthologous gene pairs identified by OrthoFinder v2.

Supplementary Table 6 List of different cis-acting elements.

Supplementary Table 7 List of primer sequences used in qRT-PCR.

Supplementary Table 8 List different Motif sequences.

Supplementary Sequence 1 List of sequences of 192 poplar R2R3 MYB proteins.

Supplementary Sequence 2 List of the CDS sequences of 192 poplar R2R3 MYB proteins.

Supplementary Sequence 3 List of the sequences of 216 willow R2R3 MYB proteins.

Supplementary Sequence 4 List of the CDS sequences of 216 willow R2R3 MYB proteins.

Supplementary Sequence 5 List of the sequences of R2R3 MYB proteins from eight species: *A. thaliana* (126 members), *P. patens* (50 members), *C. reinhardtii* (10 members), *V. vinifera* (134 members), *Z. mays* (157 members), *B. napus* (249 members), *M. domestica* (228 members) and *G. biloba* (69 members).
