# Supplementary material for: Genome-Wide Comparative Analysis of R2R3 MYB Gene Family in Populus and Salix and Identification of Male Flower Bud Development-Related Genes
Source: Front Plant Sci. 2021 Sep 14;12:721558. doi: 10.3389/fpls.2021.721558 (PMC8477045; doi:10.3389/fpls.2021.721558)
Supplement: Supplementary Figure 1 — Maximum likelihood phylogenetic tree of poplar and willow R2R3 MYB genes. [file Data_Sheet_1.ZIP › Supplementary/Supplementary_Material.docx]

Supplementary Material

# Supplementary Figures

**Supplementary Figure 1.** Maximum likelihood phylogenetic tree of poplar and willow R2R3 MYB genes.

**Supplementary Figure 2.** Distribution of exon lengths in *Populus* and *Salix* R2R3 MYB genes.

**Supplementary Figure 3.** Distribution and numbers of different motifs in the poplar and willow R2R3 MYB genes.

**Supplementary Figure 4.** Neighbor-joining phylogenetic tree of poplar, willow, *A.thaliana*, *P. patens*, *C. reinhardtii*, grapevine, maize, *B. napus*, apple, and ginkgo R2R3 MYB genes.

**Supplementary Figure 5.** Maximum likelihood phylogenetic tree of poplar, willow, *A.thaliana*, *P. patens*, *C. reinhardtii*, grapevine, maize, *B. napus*, apple, and ginkgo R2R3 MYB genes.

**Supplementary Figure 6.** Cis-element analysis of the promoter regions of GAMYB genes.

# Supplementary Tables

**Supplementary Table 1.** List of 192 identified poplar R2R3 MYB transcription factors.

**Supplementary Table 2.** List of 216 identified willow R2R3 MYB transcription factors.

**Supplementary Table 3.** List of poplar R2R3 MYB paralogous gene pairs

**Supplementary Table 4.** List of willow R2R3 MYB paralogous gene pairs

**Supplementary Table 5.** List the poplar and willow R2R3 MYB orthologous gene pairs identified by OrthoFinder v2.

**Supplementary Table 6.** List of different cis-acting elements.

**Supplementary Table 7.** List of primer sequences used in qRT-PCR.

**Supplementary Table 8.** List different Motif sequences.

# Supplementary **Sequences**

**Supplementary Sequence 1.** List of sequences of 192 poplar R2R3 MYB proteins.

**Supplementary Sequence 2.** List of the CDS sequences of 192 poplar R2R3 MYB proteins.

**Supplementary Sequence 3.** List of the sequences of 216 willow R2R3 MYB proteins.

**Supplementary Sequence 4.** List of the CDS sequences of 216 willow R2R3 MYB proteins.

**Supplementary Sequence 5.** List of the sequences of R2R3 MYB proteins from eight species: *A. thaliana* (126 members), *P. patens* (50 members), *C. reinhardtii* (10 members), *V. vinifera* (134 members), *Z. mays* (157 members) , *B. napus* (249 members), *M. domestica* (228 members) and *G. biloba* (69 members).
