# Supplementary figures and images for: Genome-Wide Comparative Analysis of R2R3 MYB Gene Family in Populus and Salix and Identification of Male Flower Bud Development-Related Genes
Source: Front Plant Sci. 2021 Sep 14;12:721558. doi: 10.3389/fpls.2021.721558 (PMC8477045; doi:10.3389/fpls.2021.721558)

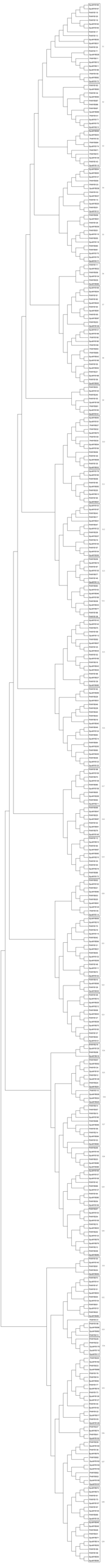

Supplement: Supplementary Figure 1 — Maximum likelihood phylogenetic tree of poplar and willow R2R3 MYB genes. [file Data_Sheet_1.ZIP › Supplementary/Supplementary Figure 1.pdf]

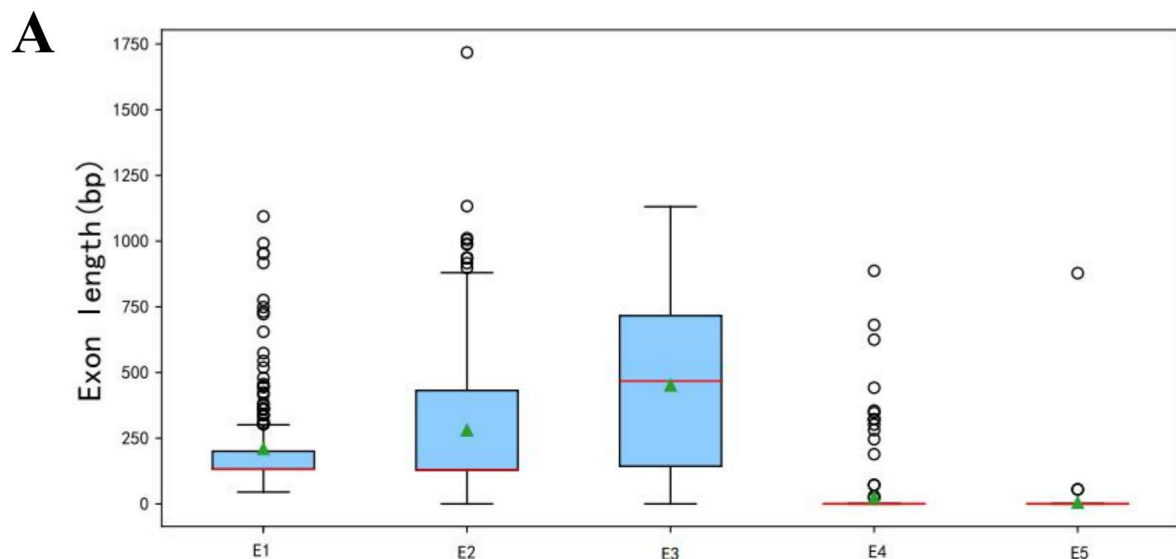

Exon CDS

Ptr 132 (33.9%) 129 (61.5%)

Spu 132 (37.5%) 129 (62.0%)

At 133 (36.8%) 130 (71.7%)

modal value(bp) & occurrence

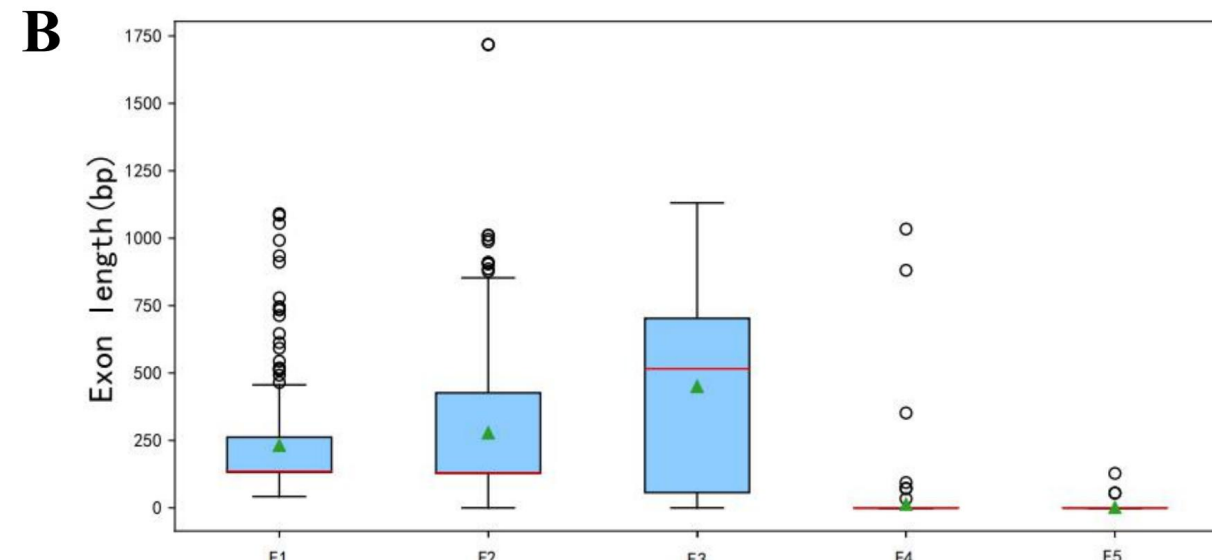

Exon CDS

Spu 132 (37.5%) 129 (62.0%)

Ptr 132 (33.9%) 129 (61.5%)

At 133 (36.8%) 130 (71.7%)

modal value(bp) & occurrence

Supplement: Supplementary Figure 1 — Maximum likelihood phylogenetic tree of poplar and willow R2R3 MYB genes. [file Data_Sheet_1.ZIP › Supplementary/Supplementary Figure 2.pdf]

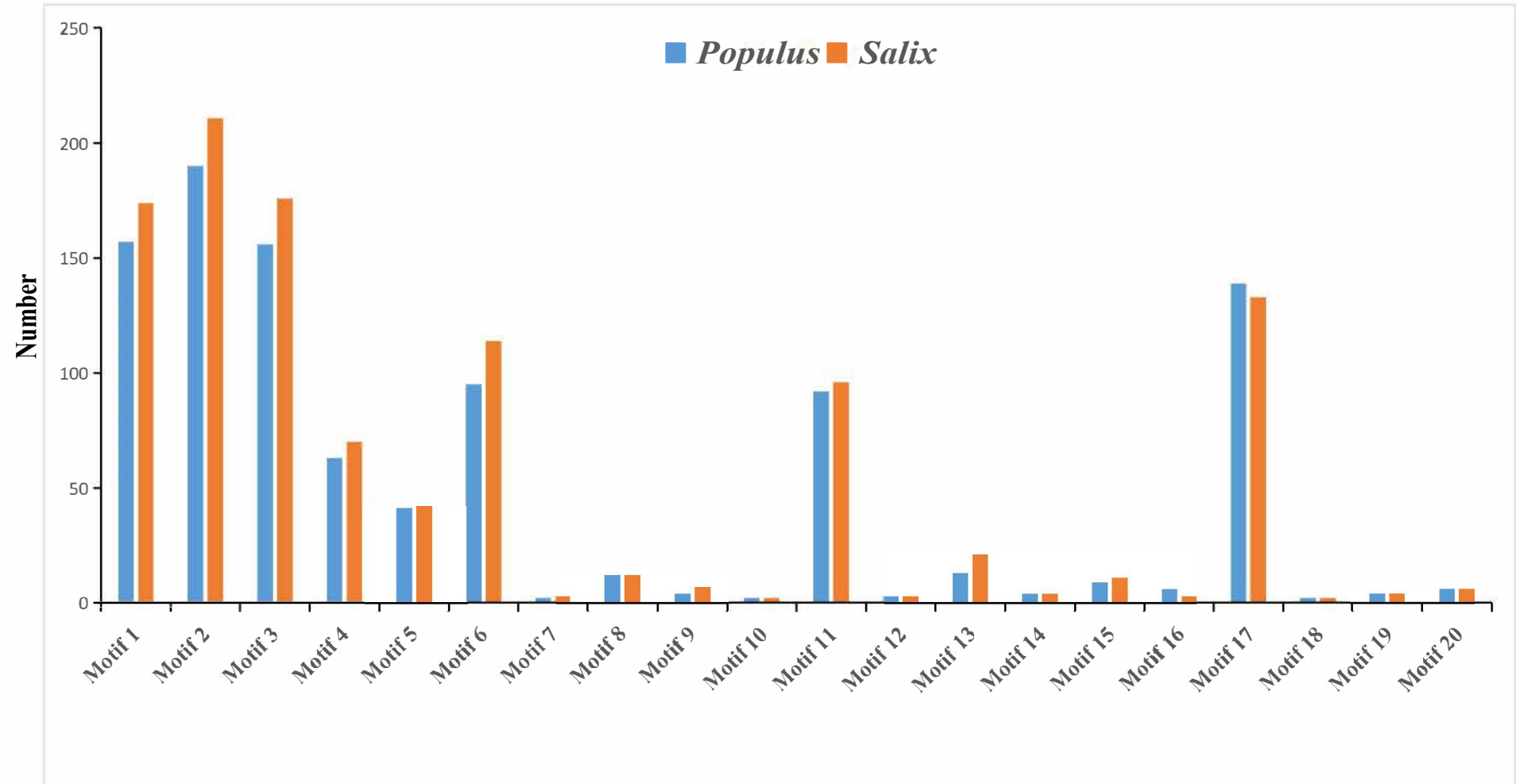

Supplement: Supplementary Figure 1 — Maximum likelihood phylogenetic tree of poplar and willow R2R3 MYB genes. [file Data_Sheet_1.ZIP › Supplementary/Supplementary Figure 3.pdf]

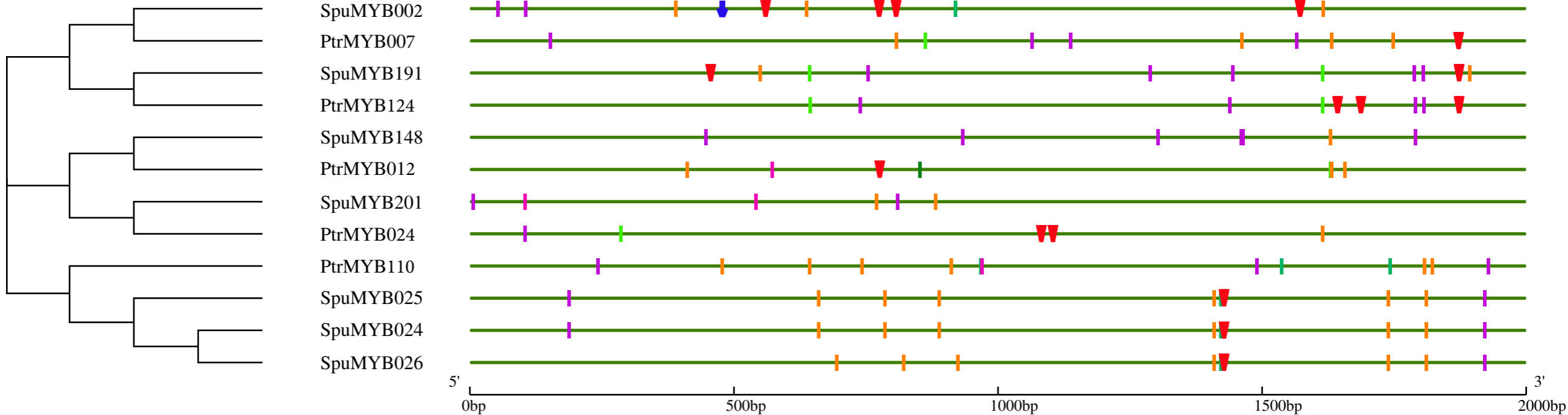

Legend:

Exon ABRE G-Box MBS MYB MYC P-box TATC-box W-box

Supplement: Supplementary Figure 1 — Maximum likelihood phylogenetic tree of poplar and willow R2R3 MYB genes. [file Data_Sheet_1.ZIP › Supplementary/Supplementary Figure 6.pdf]
