# Supplementary material for: Genome-Wide Comparative Analysis of R2R3 MYB Gene Family in Populus and Salix and Identification of Male Flower Bud Development-Related Genes
Source: Front Plant Sci. 2021 Sep 14;12:721558. doi: 10.3389/fpls.2021.721558 (PMC8477045; doi:10.3389/fpls.2021.721558)
Supplement: Supplementary Figure 1 — Maximum likelihood phylogenetic tree of poplar and willow R2R3 MYB genes. [file Supplementary Figure 4.pdf › Supplementary/Supplementary Figure 4.pdf]

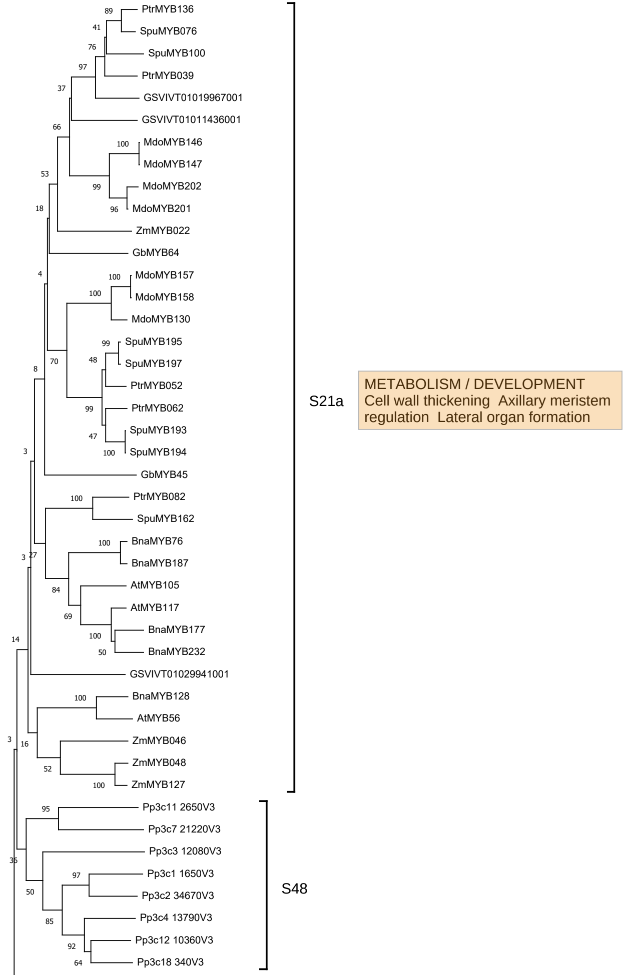
PtrMYB136
SpuMYB076
SpuMYB100
PtrMYB039
GSVIVT01019967001
GSVIVT01011436001
MdoMYB146
MdoMYB147
MdoMYB202
MdoMYB201
ZmMYB022
GbMYB64
MdoMYB157
MdoMYB158
MdoMYB130
SpuMYB195
SpuMYB197
PtrMYB052
PtrMYB062
SpuMYB193
SpuMYB194
GbMYB45
PtrMYB082
SpuMYB162
BnaMYB76
BnaMYB187
AtMYB105
AtMYB117
BnaMYB177
BnaMYB232
GSVIVT01029941001
BnaMYB128
AtMYB56
ZmMYB046
ZmMYB048
ZmMYB127
S21a
METABOLISM / DEVELOPMENT
Cell wall thickening Axillary meristem regulation Lateral organ formation
Pp3c11 2650V3
Pp3c7 21220V3
Pp3c3 12080V3
Pp3c1 1650V3
Pp3c2 34670V3
Pp3c4 13790V3
Pp3c12 10360V3
Pp3c18 340V3
S48

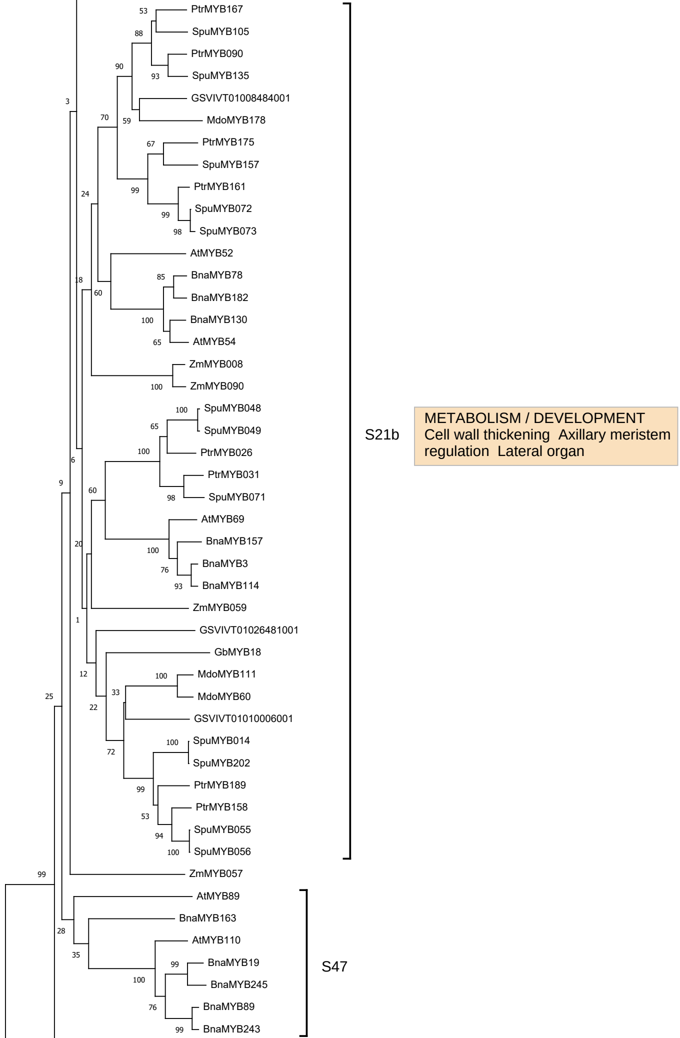
PtrMYB167
SpuMYB105
PtrMYB090
SpuMYB135
GSVIVT01008484001
MdoMYB178
PtrMYB175
SpuMYB157
PtrMYB161
SpuMYB072
SpuMYB073
AtMYB52
BnaMYB78
BnaMYB182
BnaMYB130
AtMYB54
ZmMYB008
ZmMYB090
SpuMYB048
SpuMYB049
PtrMYB026
PtrMYB031
SpuMYB071
AtMYB69
BnaMYB157
BnaMYB3
BnaMYB114
ZmMYB059
GSVIVT01026481001
GbMYB18
MdoMYB111
MdoMYB60
GSVIVT01010006001
SpuMYB014
SpuMYB202
PtrMYB189
PtrMYB158
SpuMYB055
SpuMYB056
ZmMYB057
AtMYB89
BnaMYB163
AtMYB110
BnaMYB19
BnaMYB245
BnaMYB89
BnaMYB243
S21b
METABOLISM / DEVELOPMENT
Cell wall thickening Axillary meristem regulation Lateral organ
S47

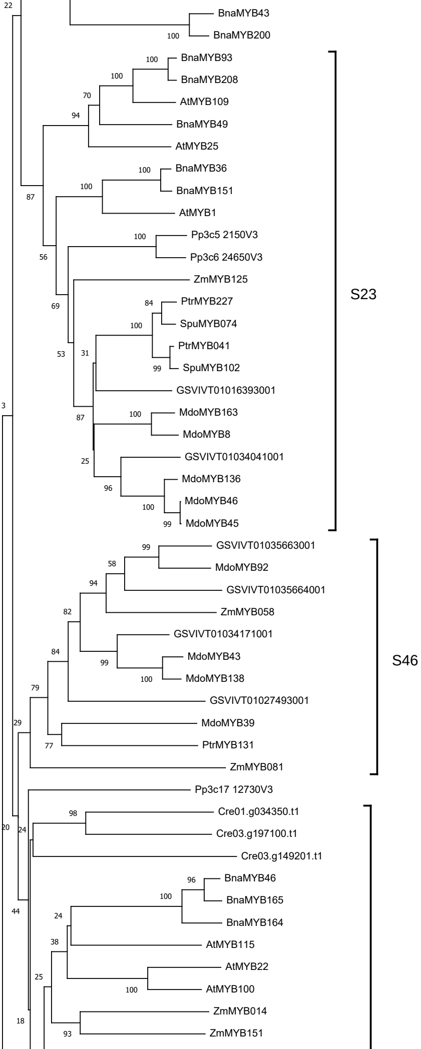

BnaMYB43
BnaMYB200
BnaMYB93
BnaMYB208
AtMYB109
BnaMYB49
AtMYB25
BnaMYB36
BnaMYB151
AtMYB1
Pp3c5 2150V3
Pp3c6 24650V3
ZmMYB125
PtrMYB227
SpuMYB074
PtrMYB041
SpuMYB102
GSVIVT01016393001
MdoMYB163
MdoMYB8
GSVIVT01034041001
MdoMYB136
MdoMYB46
MdoMYB45
S23
GSVIVT01035663001
MdoMYB92
GSVIVT01035664001
ZmMYB058
GSVIVT01034171001
MdoMYB43
MdoMYB138
GSVIVT01027493001
MdoMYB39
PtrMYB131
ZmMYB081
S46
Pp3c17 12730V3
Cre01.g034350.t1
Cre03.g197100.t1
Cre03.g149201.t1
BnaMYB46
BnaMYB165
BnaMYB164
AtMYB115
AtMYB22
AtMYB100
ZmMYB014
ZmMYB151

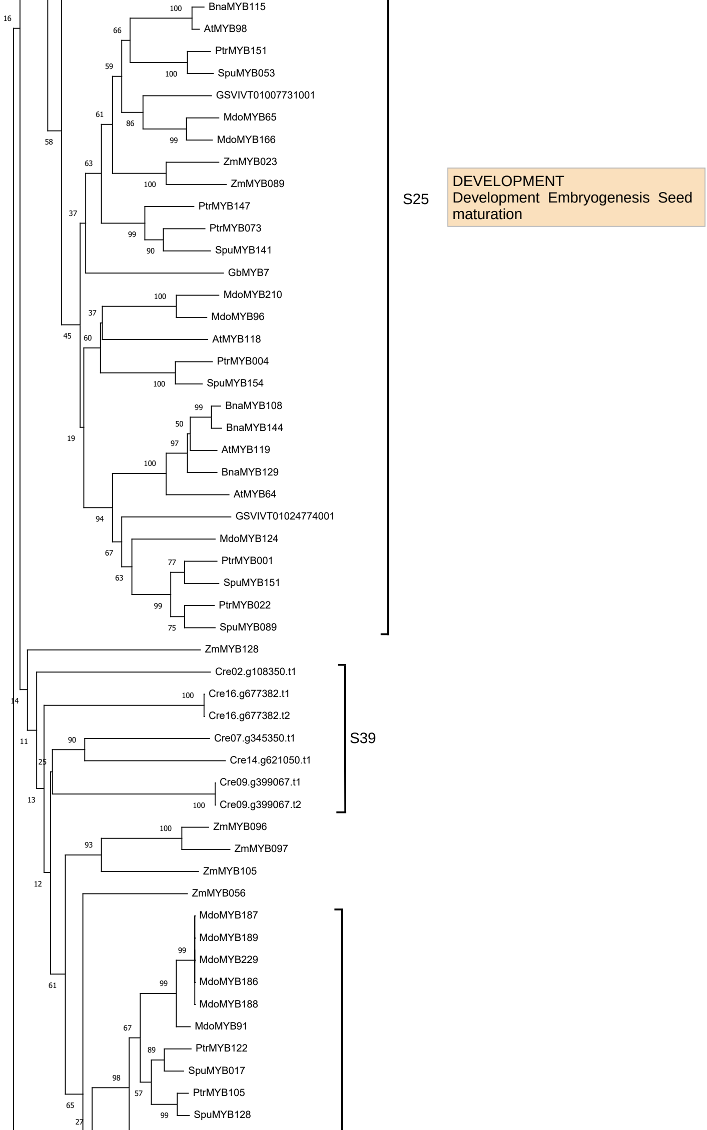
BnaMYB115
AtMYB98
PtrMYB151
SpuMYB053
GSVIVT01007731001
MdoMYB65
MdoMYB166
ZmMYB023
ZmMYB089
PtrMYB147
PtrMYB073
SpuMYB141
GbMYB7
MdoMYB210
MdoMYB96
AtMYB118
PtrMYB004
SpuMYB154
BnaMYB108
BnaMYB144
AtMYB119
BnaMYB129
AtMYB64
GSVIVT01024774001
MdoMYB124
PtrMYB001
SpuMYB151
PtrMYB022
SpuMYB089
S25
DEVELOPMENT
Development Embryogenesis Seed maturation
ZmMYB128
Cre02.g108350.t1
Cre16.g677382.t1
Cre16.g677382.t2
Cre07.g345350.t1
Cre14.g621050.t1
Cre09.g399067.t1
Cre09.g399067.t2
S39
ZmMYB096
ZmMYB097
ZmMYB105
ZmMYB056
MdoMYB187
MdoMYB189
MdoMYB229
MdoMYB186
MdoMYB188
MdoMYB91
PtrMYB122
SpuMYB017
PtrMYB105
SpuMYB128

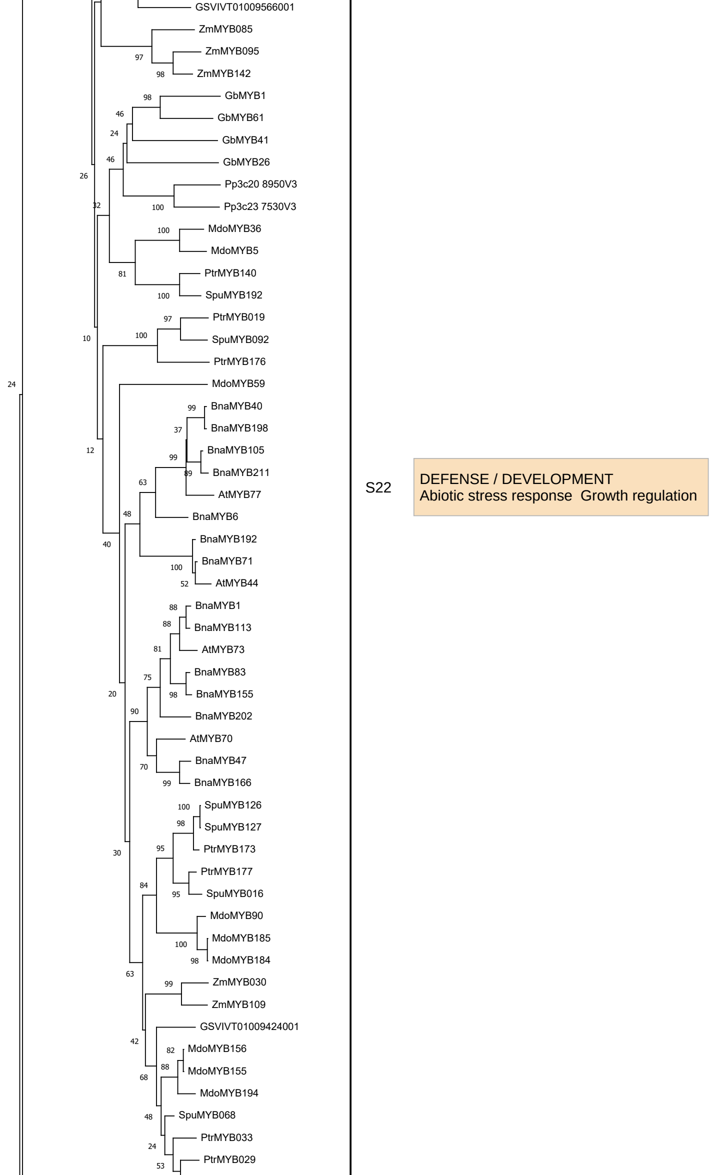
GSVIVT01009566001
ZmMYB085
ZmMYB095
ZmMYB142
GbMYB1
GbMYB61
GbMYB41
GbMYB26
Pp3c20 8950V3
Pp3c23 7530V3
MdoMYB36
MdoMYB5
PtrMYB140
SpuMYB192
PtrMYB019
SpuMYB092
PtrMYB176
MdoMYB59
BnaMYB40
BnaMYB198
BnaMYB105
BnaMYB211
AtMYB77
BnaMYB6
BnaMYB192
BnaMYB71
AtMYB44
BnaMYB1
BnaMYB113
AtMYB73
BnaMYB83
BnaMYB155
BnaMYB202
AtMYB70
BnaMYB47
BnaMYB166
SpuMYB126
SpuMYB127
PtrMYB173
PtrMYB177
SpuMYB016
MdoMYB90
MdoMYB185
MdoMYB184
ZmMYB030
ZmMYB109
GSVIVT01009424001
MdoMYB156
MdoMYB155
MdoMYB194
SpuMYB068
PtrMYB033
PtrMYB029
S22
DEFENSE / DEVELOPMENT
Abiotic stress response Growth regulation

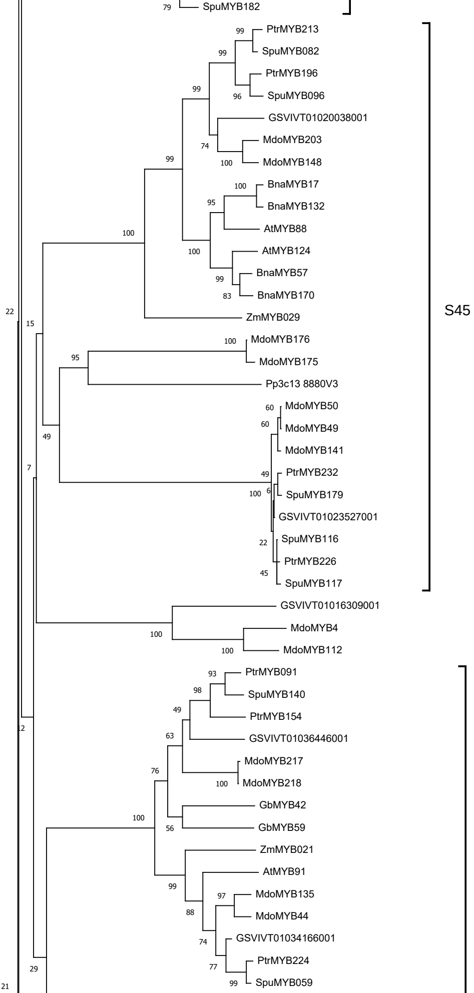

SpuMYB182
PtrMYB213
SpuMYB082
PtrMYB196
SpuMYB096
GSVIVT01020038001
MdoMYB203
MdoMYB148
BnaMYB17
BnaMYB132
AtMYB88
AtMYB124
BnaMYB57
BnaMYB170
ZmMYB029
S45
MdoMYB176
MdoMYB175
Pp3c13 8880V3
MdoMYB50
MdoMYB49
MdoMYB141
PtrMYB232
SpuMYB179
GSVIVT01023527001
SpuMYB116
PtrMYB226
SpuMYB117
GSVIVT01016309001
MdoMYB4
MdoMYB112
PtrMYB091
SpuMYB140
PtrMYB154
GSVIVT01036446001
MdoMYB217
MdoMYB218
GbMYB42
GbMYB59
ZmMYB021
AtMYB91
MdoMYB135
MdoMYB44
GSVIVT01034166001
PtrMYB224
SpuMYB059

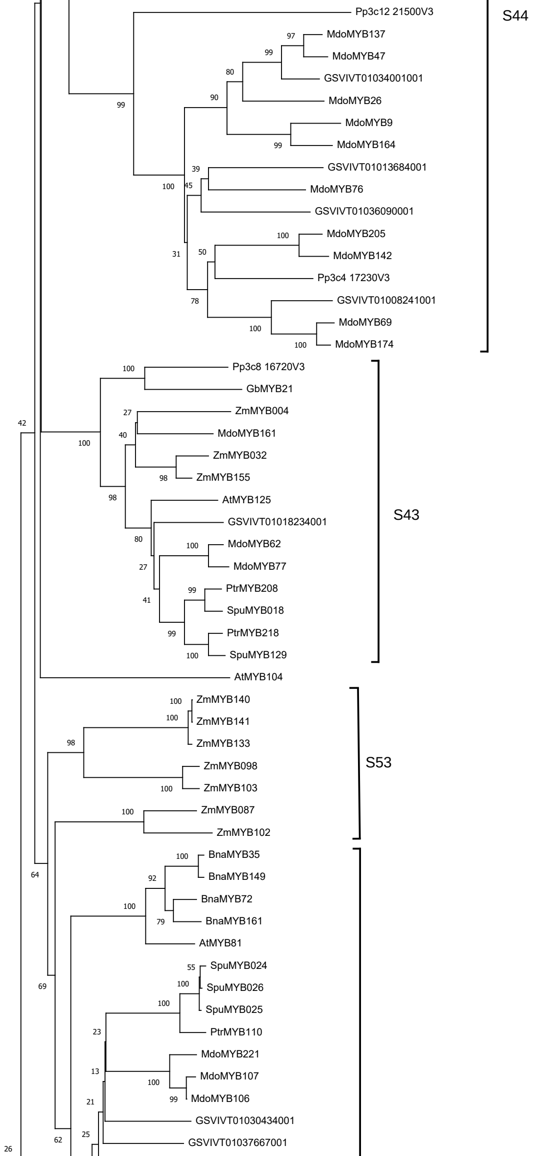

S44
Pp3c12 21500V3
MdoMYB137
MdoMYB47
GSVIVT01034001001
MdoMYB26
MdoMYB9
MdoMYB164
GSVIVT01013684001
MdoMYB76
GSVIVT01036090001
MdoMYB205
MdoMYB142
Pp3c4 17230V3
GSVIVT01008241001
MdoMYB69
MdoMYB174
S43
Pp3c8 16720V3
GbMYB21
ZmMYB004
MdoMYB161
ZmMYB032
ZmMYB155
AtMYB125
GSVIVT01018234001
MdoMYB62
MdoMYB77
PtrMYB208
SpuMYB018
PtrMYB218
SpuMYB129
AtMYB104
S53
ZmMYB140
ZmMYB141
ZmMYB133
ZmMYB098
ZmMYB103
ZmMYB087
ZmMYB102
BnaMYB35
BnaMYB149
BnaMYB72
BnaMYB161
AtMYB81
SpuMYB024
SpuMYB026
SpuMYB025
PtrMYB110
MdoMYB221
MdoMYB107
MdoMYB106
GSVIVT01030434001
GSVIVT01037667001

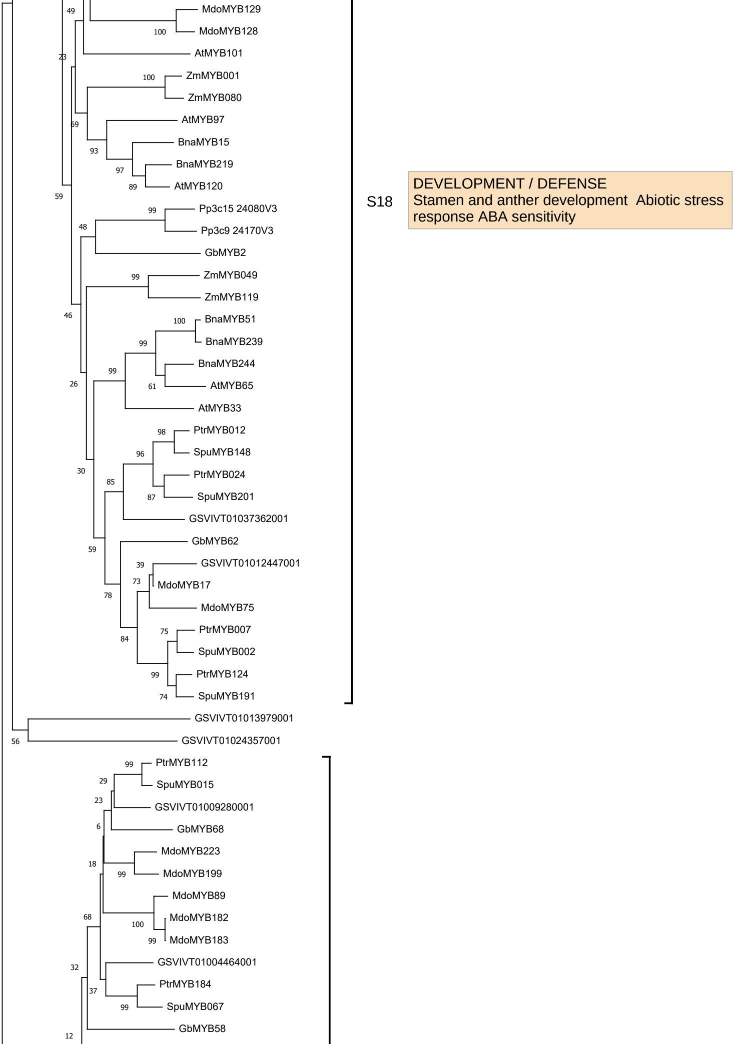

MdoMYB129
MdoMYB128
AtMYB101
ZmMYB001
ZmMYB080
AtMYB97
BnaMYB15
BnaMYB219
AtMYB120
Pp3c15 24080V3
Pp3c9 24170V3
GbMYB2
ZmMYB049
ZmMYB119
BnaMYB51
BnaMYB239
BnaMYB244
AtMYB65
AtMYB33
PtrMYB012
SpuMYB148
PtrMYB024
SpuMYB201
GSVIVT01037362001
GbMYB62
GSVIVT01012447001
MdoMYB17
MdoMYB75
PtrMYB007
SpuMYB002
PtrMYB124
SpuMYB191
GSVIVT01013979001
GSVIVT01024357001
PtrMYB112
SpuMYB015
GSVIVT01009280001
GbMYB68
MdoMYB223
MdoMYB199
MdoMYB89
MdoMYB182
MdoMYB183
GSVIVT01004464001
PtrMYB184
SpuMYB067
GbMYB58
S18
DEVELOPMENT / DEFENSE
Stamen and anther development Abiotic stress response ABA sensitivity

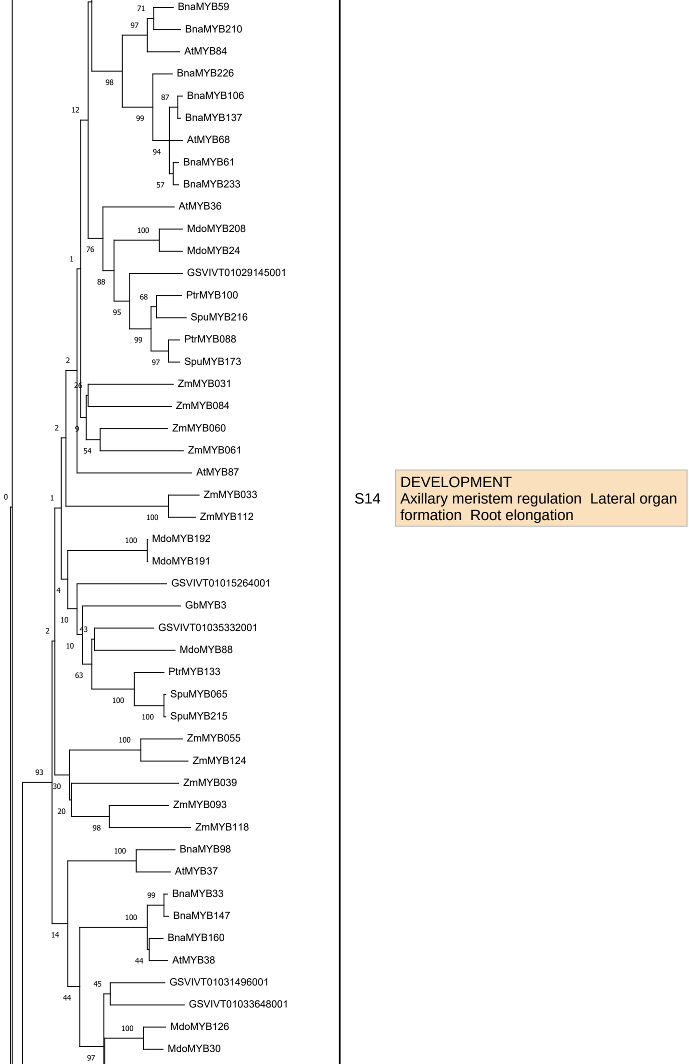

BnaMYB59
BnaMYB210
AtMYB84
BnaMYB226
BnaMYB106
BnaMYB137
AtMYB68
BnaMYB61
BnaMYB233
AtMYB36
MdoMYB208
MdoMYB24
GSVIVT01029145001
PtrMYB100
SpuMYB216
PtrMYB088
SpuMYB173
ZmMYB031
ZmMYB084
ZmMYB060
ZmMYB061
AtMYB87
ZmMYB033
ZmMYB112
MdoMYB192
MdoMYB191
GSVIVT01015264001
GbMYB3
GSVIVT01035332001
MdoMYB88
PtrMYB133
SpuMYB065
SpuMYB215
ZmMYB055
ZmMYB124
ZmMYB039
ZmMYB093
ZmMYB118
BnaMYB98
AtMYB37
BnaMYB33
BnaMYB147
BnaMYB160
AtMYB38
GSVIVT01031496001
GSVIVT01033648001
MdoMYB126
MdoMYB30
S14
DEVELOPMENT
Axillary meristem regulation Lateral organ formation Root elongation

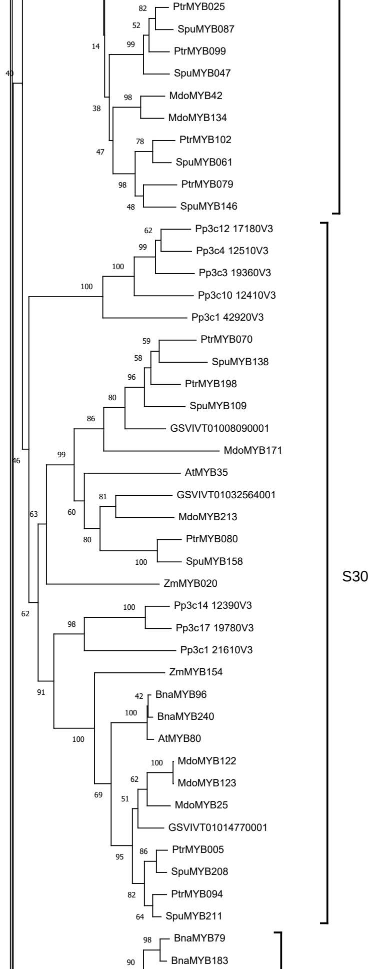
PtrMYB025
SpuMYB087
PtrMYB099
SpuMYB047
MdoMYB42
MdoMYB134
PtrMYB102
SpuMYB061
PtrMYB079
SpuMYB146
Pp3c12 17180V3
Pp3c4 12510V3
Pp3c3 19360V3
Pp3c10 12410V3
Pp3c1 42920V3
PtrMYB070
SpuMYB138
PtrMYB198
SpuMYB109
GSVIVT01008090001
MdoMYB171
AtMYB35
GSVIVT01032564001
MdoMYB213
PtrMYB080
SpuMYB158
ZmMYB020
Pp3c14 12390V3
Pp3c17 19780V3
Pp3c1 21610V3
ZmMYB154
BnaMYB96
BnaMYB240
AtMYB80
MdoMYB122
MdoMYB123
MdoMYB25
GSVIVT01014770001
PtrMYB005
SpuMYB208
PtrMYB094
SpuMYB211
BnaMYB79
BnaMYB183
S30

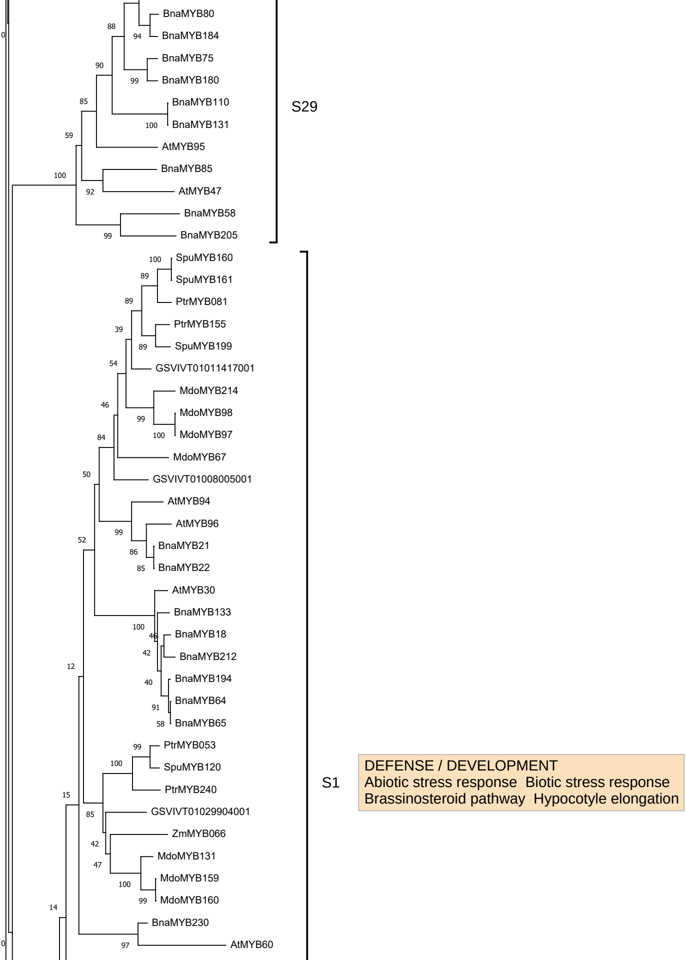

BnaMYB80
BnaMYB184
BnaMYB75
BnaMYB180
BnaMYB110
BnaMYB131
AtMYB95
BnaMYB85
AtMYB47
BnaMYB58
BnaMYB205
S29
SpuMYB160
SpuMYB161
PtrMYB081
PtrMYB155
SpuMYB199
GSVIVT01011417001
MdoMYB214
MdoMYB98
MdoMYB97
MdoMYB67
GSVIVT01008005001
AtMYB94
AtMYB96
BnaMYB21
BnaMYB22
AtMYB30
BnaMYB133
BnaMYB18
BnaMYB212
BnaMYB194
BnaMYB64
BnaMYB65
PtrMYB053
SpuMYB120
PtrMYB240
GSVIVT01029904001
ZmMYB066
MdoMYB131
MdoMYB159
MdoMYB160
BnaMYB230
AtMYB60
S1
DEFENSE / DEVELOPMENT
Abiotic stress response Biotic stress response
Brassinosteroid pathway Hypocotyle elongation

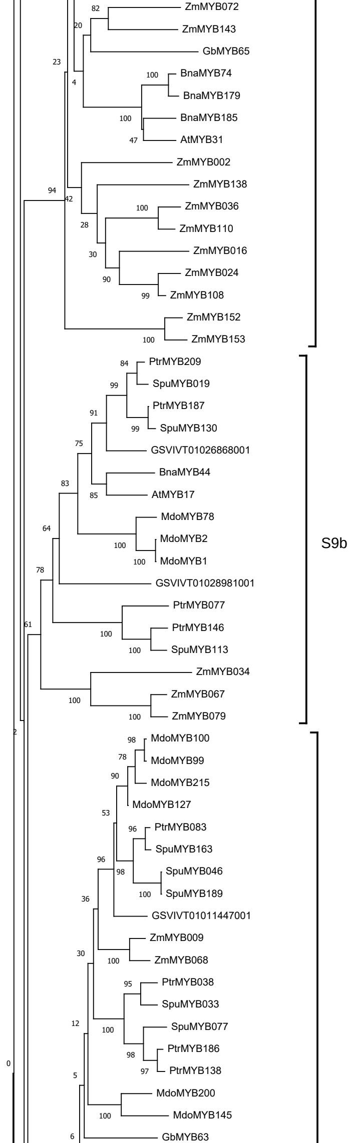
ZmMYB072
ZmMYB143
GbMYB65
BnaMYB74
BnaMYB179
BnaMYB185
AtMYB31
ZmMYB002
ZmMYB138
ZmMYB036
ZmMYB110
ZmMYB016
ZmMYB024
ZmMYB108
ZmMYB152
ZmMYB153
PtrMYB209
SpuMYB019
PtrMYB187
SpuMYB130
GSVIVT01026868001
BnaMYB44
AtMYB17
MdoMYB78
MdoMYB2
MdoMYB1
GSVIVT01028981001
PtrMYB077
PtrMYB146
SpuMYB113
ZmMYB034
ZmMYB067
ZmMYB079
S9b
MdoMYB100
MdoMYB99
MdoMYB215
MdoMYB127
PtrMYB083
SpuMYB163
SpuMYB046
SpuMYB189
GSVIVT01011447001
ZmMYB009
ZmMYB068
PtrMYB038
SpuMYB033
SpuMYB077
PtrMYB186
PtrMYB138
MdoMYB200
MdoMYB145
GbMYB63

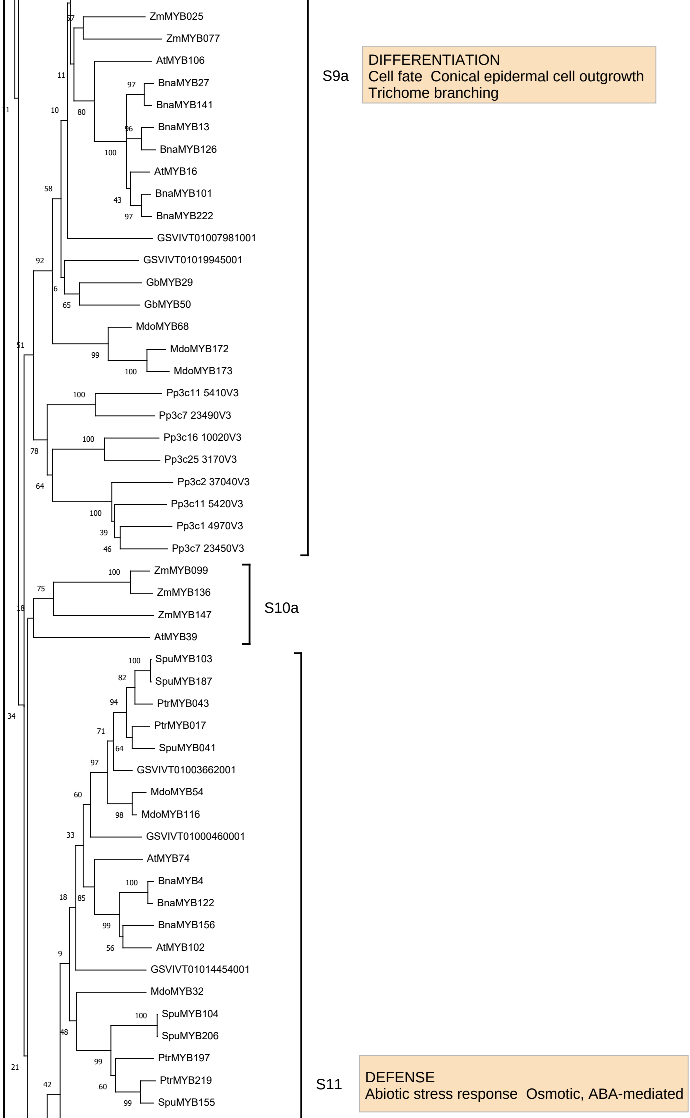

ZmMYB025
ZmMYB077
AtMYB106
BnaMYB27
BnaMYB141
BnaMYB13
BnaMYB126
AtMYB16
BnaMYB101
BnaMYB222
GSVIVT01007981001
GSVIVT01019945001
GbMYB29
GbMYB50
MdoMYB68
MdoMYB172
MdoMYB173
Pp3c11 5410V3
Pp3c7 23490V3
Pp3c16 10020V3
Pp3c25 3170V3
Pp3c2 37040V3
Pp3c11 5420V3
Pp3c1 4970V3
Pp3c7 23450V3
ZmMYB099
ZmMYB136
ZmMYB147
AtMYB39
SpuMYB103
SpuMYB187
PtrMYB043
PtrMYB017
SpuMYB041
GSVIVT01003662001
MdoMYB54
MdoMYB116
GSVIVT01000460001
AtMYB74
BnaMYB4
BnaMYB122
BnaMYB156
AtMYB102
GSVIVT01014454001
MdoMYB32
SpuMYB104
SpuMYB206
PtrMYB197
PtrMYB219
SpuMYB155
S9a
DIFFERENTIATION
Cell fate Conical epidermal cell outgrowth
Trichome branching
S10a
S11
DEFENSE
Abiotic stress response Osmotic, ABA-mediated

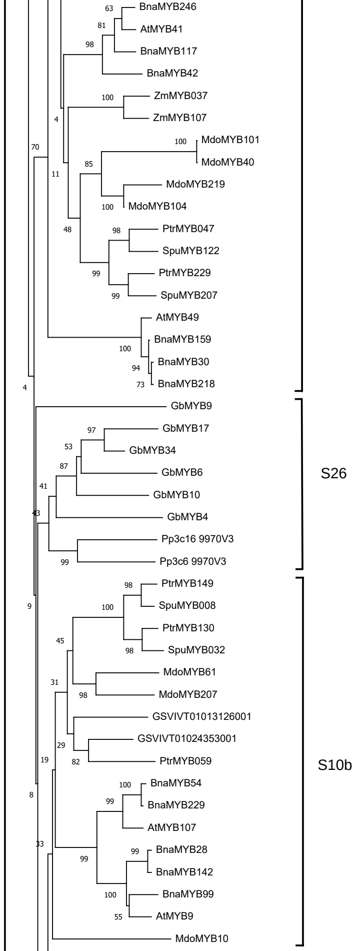
BnaMYB246
AtMYB41
BnaMYB117
BnaMYB42
ZmMYB037
ZmMYB107
MdoMYB101
MdoMYB40
MdoMYB219
MdoMYB104
PtrMYB047
SpuMYB122
PtrMYB229
SpuMYB207
AtMYB49
BnaMYB159
BnaMYB30
BnaMYB218
GbMYB9
GbMYB17
GbMYB34
GbMYB6
GbMYB10
GbMYB4
Pp3c16 9970V3
Pp3c6 9970V3
S26
PtrMYB149
SpuMYB008
PtrMYB130
SpuMYB032
MdoMYB61
MdoMYB207
GSVIVT01013126001
GSVIVT01024353001
PtrMYB059
BnaMYB54
BnaMYB229
AtMYB107
BnaMYB28
BnaMYB142
BnaMYB99
AtMYB9
MdoMYB10
S10b

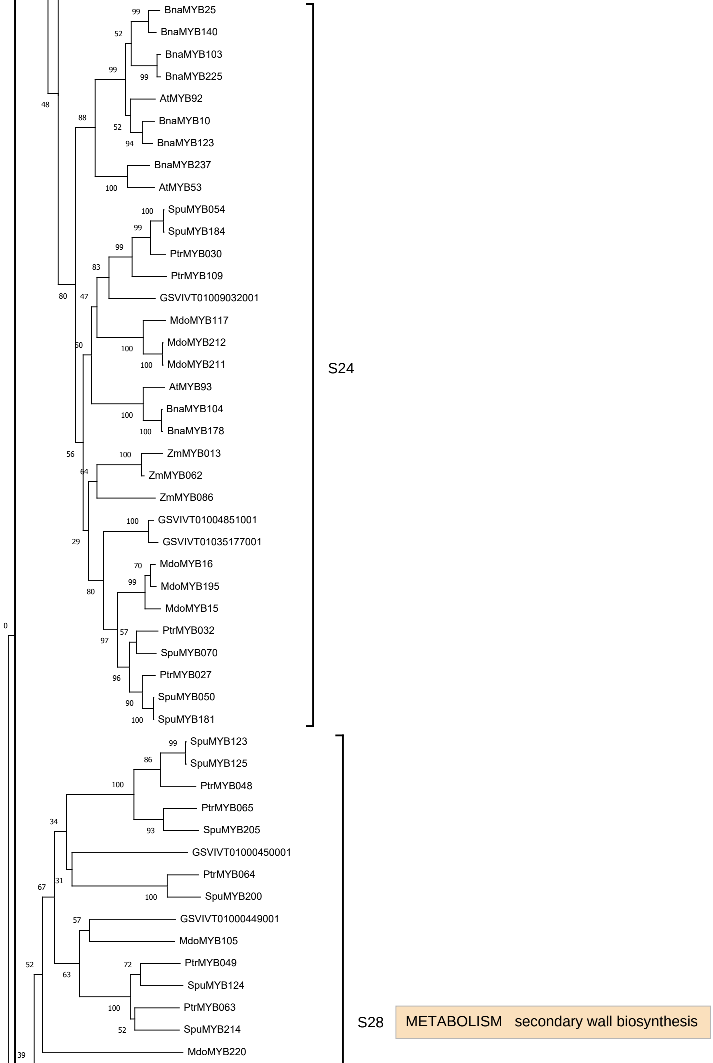

BnaMYB25
BnaMYB140
BnaMYB103
BnaMYB225
AtMYB92
BnaMYB10
BnaMYB123
BnaMYB237
AtMYB53
SpuMYB054
SpuMYB184
PtrMYB030
PtrMYB109
GSVIVT01009032001
MdoMYB117
MdoMYB212
MdoMYB211
AtMYB93
BnaMYB104
BnaMYB178
ZmMYB013
ZmMYB062
ZmMYB086
GSVIVT01004851001
GSVIVT01035177001
MdoMYB16
MdoMYB195
MdoMYB15
PtrMYB032
SpuMYB070
PtrMYB027
SpuMYB050
SpuMYB181
S24
SpuMYB123
SpuMYB125
PtrMYB048
PtrMYB065
SpuMYB205
GSVIVT01000450001
PtrMYB064
SpuMYB200
GSVIVT01000449001
MdoMYB105
PtrMYB049
SpuMYB124
PtrMYB063
SpuMYB214
MdoMYB220
S28
METABOLISM secondary wall biosynthesis

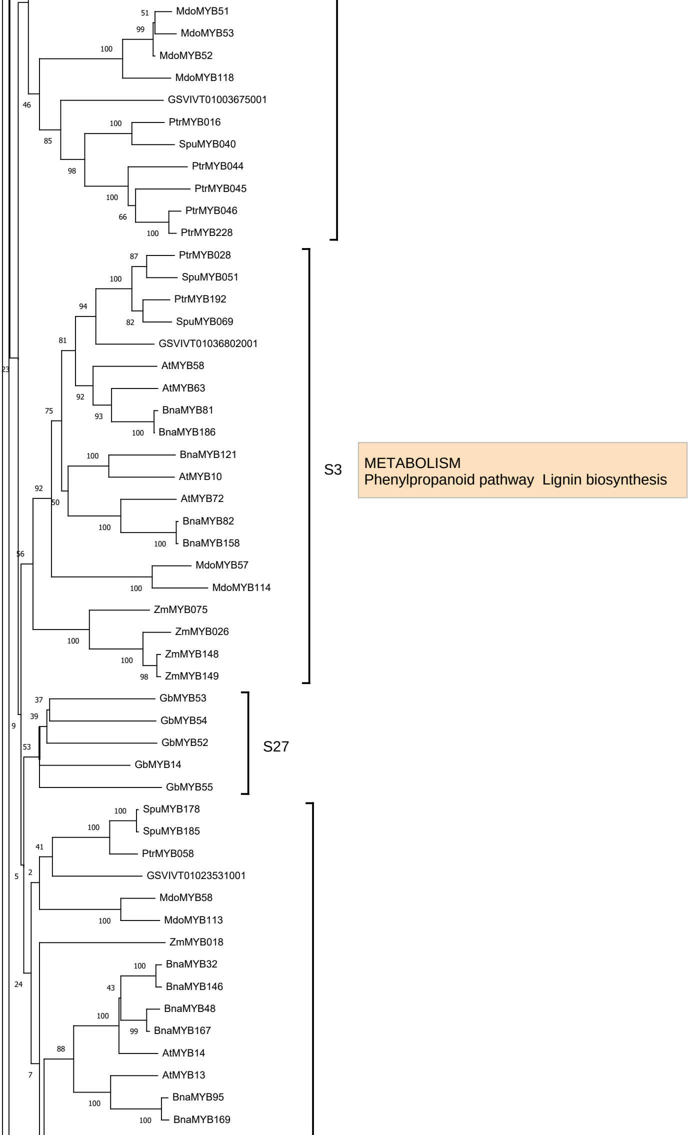
MdoMYB51
MdoMYB53
MdoMYB52
MdoMYB118
GSVIVT01003675001
PtrMYB016
SpuMYB040
PtrMYB044
PtrMYB045
PtrMYB046
PtrMYB228
PtrMYB028
SpuMYB051
PtrMYB192
SpuMYB069
GSVIVT01036802001
AtMYB58
AtMYB63
BnaMYB81
BnaMYB186
BnaMYB121
AtMYB10
AtMYB72
BnaMYB82
BnaMYB158
MdoMYB57
MdoMYB114
ZmMYB075
ZmMYB026
ZmMYB148
ZmMYB149
S3
METABOLISM
Phenylpropanoid pathway Lignin biosynthesis
GbMYB53
GbMYB54
GbMYB52
GbMYB14
GbMYB55
S27
SpuMYB178
SpuMYB185
PtrMYB058
GSVIVT01023531001
MdoMYB58
MdoMYB113
ZmMYB018
BnaMYB32
BnaMYB146
BnaMYB48
BnaMYB167
AtMYB14
AtMYB13
BnaMYB95
BnaMYB169

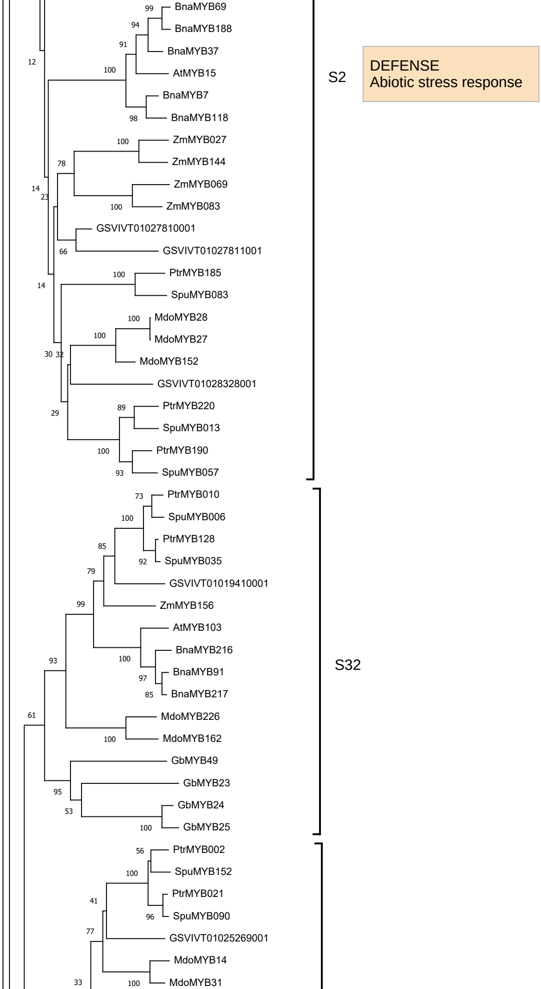

BnaMYB69
BnaMYB188
BnaMYB37
AtMYB15
BnaMYB7
BnaMYB118
ZmMYB027
ZmMYB144
ZmMYB069
ZmMYB083
GSVIVT01027810001
GSVIVT01027811001
PtrMYB185
SpuMYB083
MdoMYB28
MdoMYB27
MdoMYB152
GSVIVT01028328001
PtrMYB220
SpuMYB013
PtrMYB190
SpuMYB057
S2
DEFENSE
Abiotic stress response
PtrMYB010
SpuMYB006
PtrMYB128
SpuMYB035
GSVIVT01019410001
ZmMYB156
AtMYB103
BnaMYB216
BnaMYB91
BnaMYB217
MdoMYB226
MdoMYB162
GbMYB49
GbMYB23
GbMYB24
GbMYB25
S32
PtrMYB002
SpuMYB152
PtrMYB021
SpuMYB090
GSVIVT01025269001
MdoMYB14
MdoMYB31

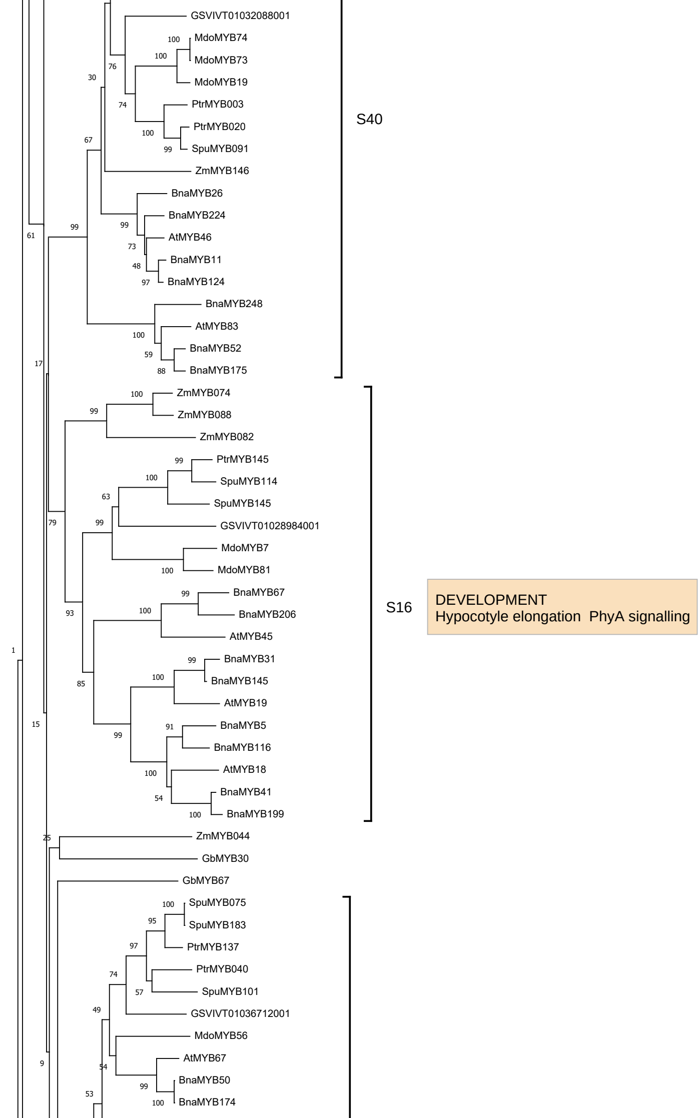
GSVIVT01032088001
MdoMYB74
MdoMYB73
MdoMYB19
PtrMYB003
PtrMYB020
SpuMYB091
ZmMYB146
BnaMYB26
BnaMYB224
AtMYB46
BnaMYB11
BnaMYB124
BnaMYB248
AtMYB83
BnaMYB52
BnaMYB175
S40
ZmMYB074
ZmMYB088
ZmMYB082
PtrMYB145
SpuMYB114
SpuMYB145
GSVIVT01028984001
MdoMYB7
MdoMYB81
BnaMYB67
BnaMYB206
AtMYB45
BnaMYB31
BnaMYB145
AtMYB19
BnaMYB5
BnaMYB116
AtMYB18
BnaMYB41
BnaMYB199
S16
DEVELOPMENT
Hypocotyle elongation PhyA signalling
ZmMYB044
GbMYB30
GbMYB67
SpuMYB075
SpuMYB183
PtrMYB137
PtrMYB040
SpuMYB101
GSVIVT01036712001
MdoMYB56
AtMYB67
BnaMYB50
BnaMYB174

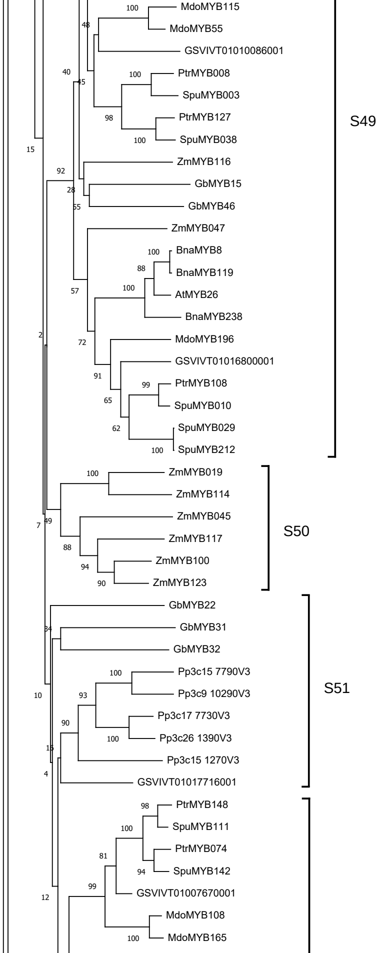

MdoMYB115
MdoMYB55
GSVIVT01010086001
PtrMYB008
SpuMYB003
PtrMYB127
SpuMYB038
ZmMYB116
GbMYB15
GbMYB46
ZmMYB047
BnaMYB8
BnaMYB119
AtMYB26
BnaMYB238
MdoMYB196
GSVIVT01016800001
PtrMYB108
SpuMYB010
SpuMYB029
SpuMYB212
S49
ZmMYB019
ZmMYB114
ZmMYB045
ZmMYB117
ZmMYB100
ZmMYB123
S50
GbMYB22
GbMYB31
GbMYB32
Pp3c15 7790V3
Pp3c9 10290V3
Pp3c17 7730V3
Pp3c26 1390V3
Pp3c15 1270V3
GSVIVT01017716001
S51
PtrMYB148
SpuMYB111
PtrMYB074
SpuMYB142
GSVIVT01007670001
MdoMYB108
MdoMYB165

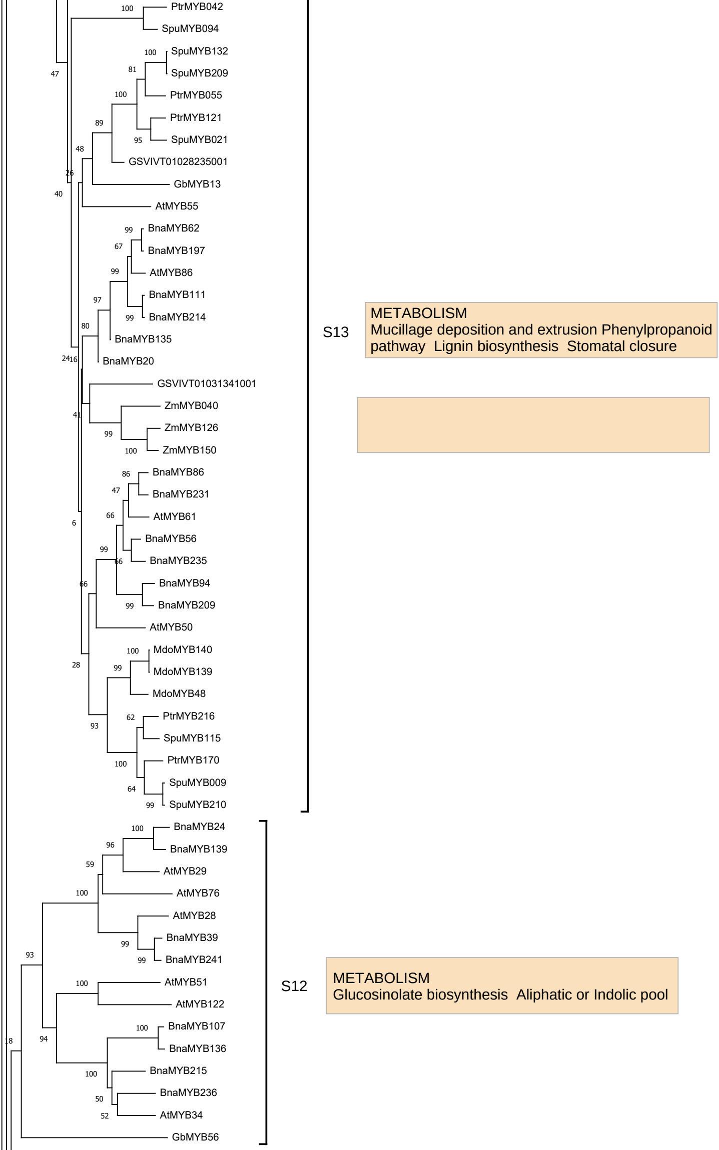
PtrMYB042
SpuMYB094
SpuMYB132
SpuMYB209
PtrMYB055
PtrMYB121
SpuMYB021
GSVIVT01028235001
GbMYB13
AtMYB55
BnaMYB62
BnaMYB197
AtMYB86
BnaMYB111
BnaMYB214
BnaMYB135
BnaMYB20
GSVIVT01031341001
ZmMYB040
ZmMYB126
ZmMYB150
BnaMYB86
BnaMYB231
AtMYB61
BnaMYB56
BnaMYB235
BnaMYB94
BnaMYB209
AtMYB50
MdoMYB140
MdoMYB139
MdoMYB48
PtrMYB216
SpuMYB115
PtrMYB170
SpuMYB009
SpuMYB210
S13
METABOLISM
Mucillage deposition and extrusion Phenylpropanoid pathway Lignin biosynthesis Stomatal closure
BnaMYB24
BnaMYB139
AtMYB29
AtMYB76
AtMYB28
BnaMYB39
BnaMYB241
AtMYB51
AtMYB122
BnaMYB107
BnaMYB136
BnaMYB215
BnaMYB236
AtMYB34
GbMYB56
S12
METABOLISM
Glucosinolate biosynthesis Aliphatic or Indolic pool

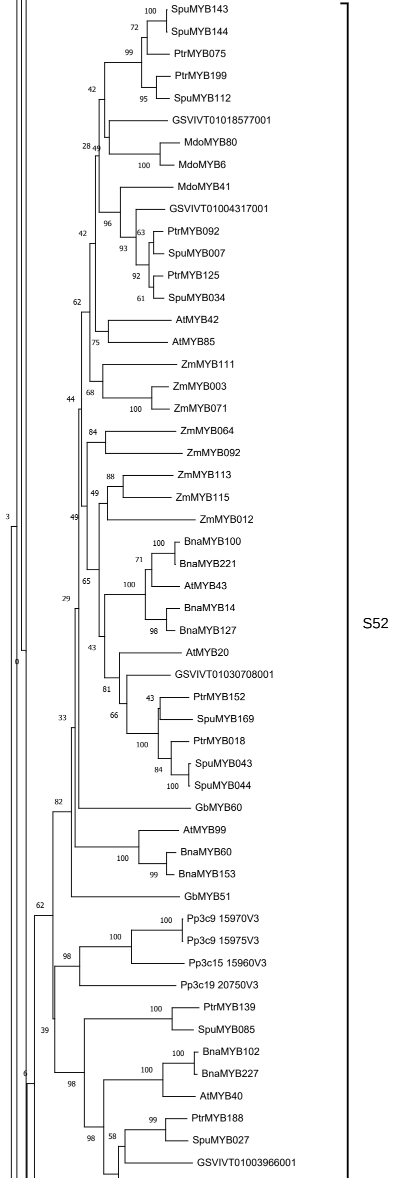
SpuMYB143
SpuMYB144
PtrMYB075
PtrMYB199
SpuMYB112
GSVIVT01018577001
MdoMYB80
MdoMYB6
MdoMYB41
GSVIVT01004317001
PtrMYB092
SpuMYB007
PtrMYB125
SpuMYB034
AtMYB42
AtMYB85
ZmMYB111
ZmMYB003
ZmMYB071
ZmMYB064
ZmMYB092
ZmMYB113
ZmMYB115
ZmMYB012
BnaMYB100
BnaMYB221
AtMYB43
BnaMYB14
BnaMYB127
AtMYB20
GSVIVT01030708001
PtrMYB152
SpuMYB169
PtrMYB018
SpuMYB043
SpuMYB044
GbMYB60
AtMYB99
BnaMYB60
BnaMYB153
GbMYB51
Pp3c9 15970V3
Pp3c9 15975V3
Pp3c15 15960V3
Pp3c19 20750V3
PtrMYB139
SpuMYB085
BnaMYB102
BnaMYB227
AtMYB40
PtrMYB188
SpuMYB027
GSVIVT01003966001
S52

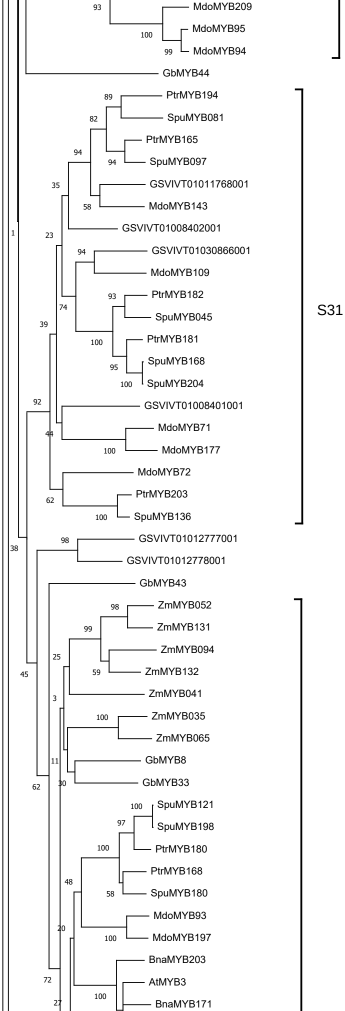
MdoMYB209
MdoMYB95
MdoMYB94
GbMYB44
PtrMYB194
SpuMYB081
PtrMYB165
SpuMYB097
GSVIVT01011768001
MdoMYB143
GSVIVT01008402001
GSVIVT01030866001
MdoMYB109
PtrMYB182
SpuMYB045
PtrMYB181
SpuMYB168
SpuMYB204
GSVIVT01008401001
MdoMYB71
MdoMYB177
MdoMYB72
PtrMYB203
SpuMYB136
S31
GSVIVT01012777001
GSVIVT01012778001
GbMYB43
ZmMYB052
ZmMYB131
ZmMYB094
ZmMYB132
ZmMYB041
ZmMYB035
ZmMYB065
GbMYB8
GbMYB33
SpuMYB121
SpuMYB198
PtrMYB180
PtrMYB168
SpuMYB180
MdoMYB93
MdoMYB197
BnaMYB203
AtMYB3
BnaMYB171

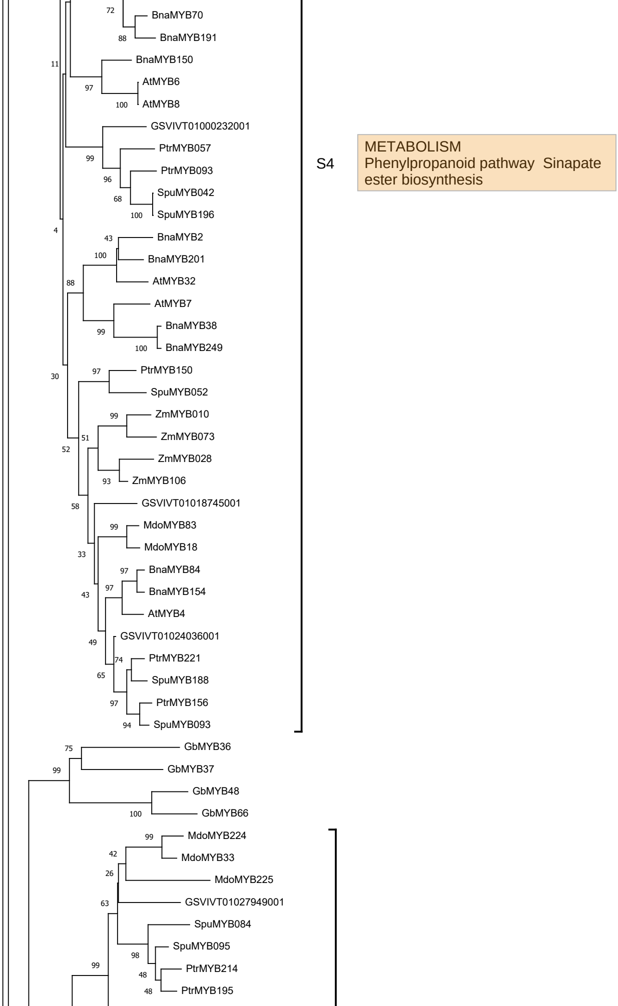

72
BnaMYB70
88
BnaMYB191
11
BnaMYB150
AtMYB6
97
100
AtMYB8
GSVIVT01000232001
PtrMYB057
99
PtrMYB093
96
SpuMYB042
68
100
SpuMYB196
4
43
BnaMYB2
100
BnaMYB201
AtMYB32
88
AtMYB7
BnaMYB38
99
100
BnaMYB249
97
PtrMYB150
30
SpuMYB052
99
ZmMYB010
ZmMYB073
51
52
ZmMYB028
93
ZmMYB106
GSVIVT01018745001
58
99
MdoMYB83
MdoMYB18
33
97
BnaMYB84
97
BnaMYB154
43
AtMYB4
GSVIVT01024036001
49
74
PtrMYB221
65
SpuMYB188
PtrMYB156
97
94
SpuMYB093
75
GbMYB36
99
GbMYB37
GbMYB48
100
GbMYB66
99
MdoMYB224
42
MdoMYB33
26
MdoMYB225
63
GSVIVT01027949001
SpuMYB084
SpuMYB095
98
99
PtrMYB214
48
48
PtrMYB195
S4
METABOLISM
Phenylpropanoid pathway Sinapate ester biosynthesis

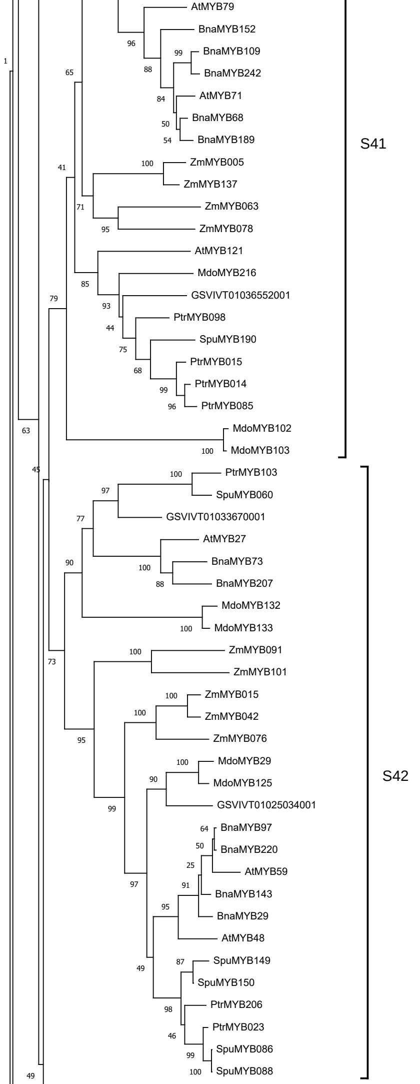

AtMYB79
BnaMYB152
BnaMYB109
BnaMYB242
AtMYB71
BnaMYB68
BnaMYB189
ZmMYB005
ZmMYB137
ZmMYB063
ZmMYB078
AtMYB121
MdoMYB216
GSVIVT01036552001
PtrMYB098
SpuMYB190
PtrMYB015
PtrMYB014
PtrMYB085
MdoMYB102
MdoMYB103
S41
PtrMYB103
SpuMYB060
GSVIVT01033670001
AtMYB27
BnaMYB73
BnaMYB207
MdoMYB132
MdoMYB133
ZmMYB091
ZmMYB101
ZmMYB015
ZmMYB042
ZmMYB076
MdoMYB29
MdoMYB125
GSVIVT01025034001
BnaMYB97
BnaMYB220
AtMYB59
BnaMYB143
BnaMYB29
AtMYB48
SpuMYB149
SpuMYB150
PtrMYB206
PtrMYB023
SpuMYB086
SpuMYB088
S42

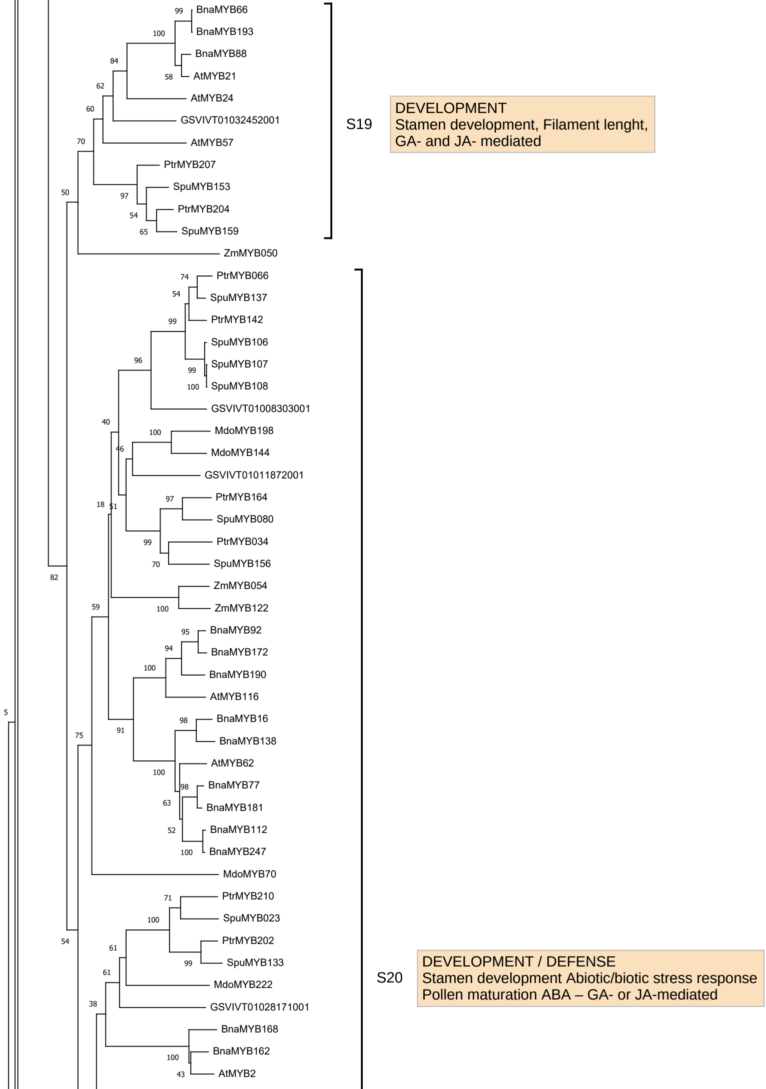

BnaMYB66
BnaMYB193
BnaMYB88
AtMYB21
AtMYB24
GSVIVT01032452001
AtMYB57
PtrMYB207
SpuMYB153
PtrMYB204
SpuMYB159
ZmMYB050
S19
DEVELOPMENT
Stamen development, Filament lenght,
GA- and JA- mediated
PtrMYB066
SpuMYB137
PtrMYB142
SpuMYB106
SpuMYB107
SpuMYB108
GSVIVT01008303001
MdoMYB198
MdoMYB144
GSVIVT01011872001
PtrMYB164
SpuMYB080
PtrMYB034
SpuMYB156
ZmMYB054
ZmMYB122
BnaMYB92
BnaMYB172
BnaMYB190
AtMYB116
BnaMYB16
BnaMYB138
AtMYB62
BnaMYB77
BnaMYB181
BnaMYB112
BnaMYB247
MdoMYB70
PtrMYB210
SpuMYB023
PtrMYB202
SpuMYB133
MdoMYB222
GSVIVT01028171001
BnaMYB168
BnaMYB162
AtMYB2
S20
DEVELOPMENT / DEFENSE
Stamen development Abiotic/biotic stress response
Pollen maturation ABA – GA- or JA-mediated

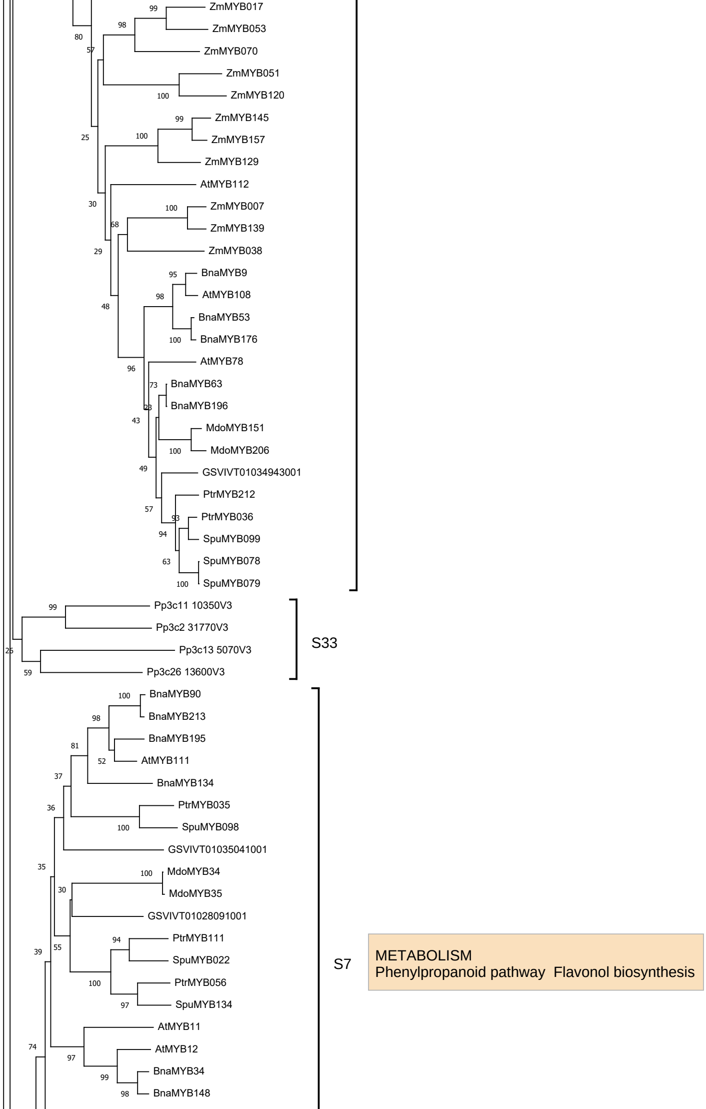
ZmMYB017
ZmMYB053
ZmMYB070
ZmMYB051
ZmMYB120
ZmMYB145
ZmMYB157
ZmMYB129
AtMYB112
ZmMYB007
ZmMYB139
ZmMYB038
BnaMYB9
AtMYB108
BnaMYB53
BnaMYB176
AtMYB78
BnaMYB63
BnaMYB196
MdoMYB151
MdoMYB206
GSVIVT01034943001
PtrMYB212
PtrMYB036
SpuMYB099
SpuMYB078
SpuMYB079
Pp3c11 10350V3
Pp3c2 31770V3
Pp3c13 5070V3
Pp3c26 13600V3
S33
BnaMYB90
BnaMYB213
BnaMYB195
AtMYB111
BnaMYB134
PtrMYB035
SpuMYB098
GSVIVT01035041001
MdoMYB34
MdoMYB35
GSVIVT01028091001
PtrMYB111
SpuMYB022
PtrMYB056
SpuMYB134
AtMYB11
AtMYB12
BnaMYB34
BnaMYB148
S7
METABOLISM
Phenylpropanoid pathway Flavonol biosynthesis

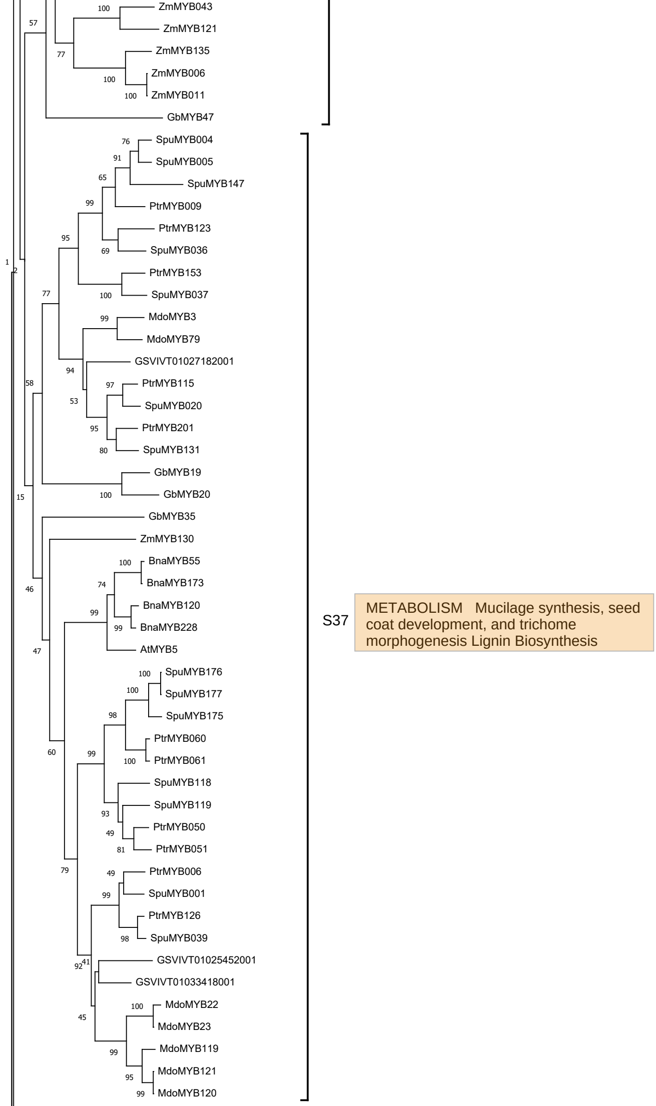
ZmMYB043
ZmMYB121
ZmMYB135
ZmMYB006
ZmMYB011
GbMYB47
SpuMYB004
SpuMYB005
SpuMYB147
PtrMYB009
PtrMYB123
SpuMYB036
PtrMYB153
SpuMYB037
MdoMYB3
MdoMYB79
GSVIVT01027182001
PtrMYB115
SpuMYB020
PtrMYB201
SpuMYB131
GbMYB19
GbMYB20
GbMYB35
ZmMYB130
BnaMYB55
BnaMYB173
BnaMYB120
BnaMYB228
AtMYB5
SpuMYB176
SpuMYB177
SpuMYB175
PtrMYB060
PtrMYB061
SpuMYB118
SpuMYB119
PtrMYB050
PtrMYB051
PtrMYB006
SpuMYB001
PtrMYB126
SpuMYB039
GSVIVT01025452001
GSVIVT01033418001
MdoMYB22
MdoMYB23
MdoMYB119
MdoMYB121
MdoMYB120
S37
METABOLISM Mucilage synthesis, seed coat development, and trichome morphogenesis Lignin Biosynthesis

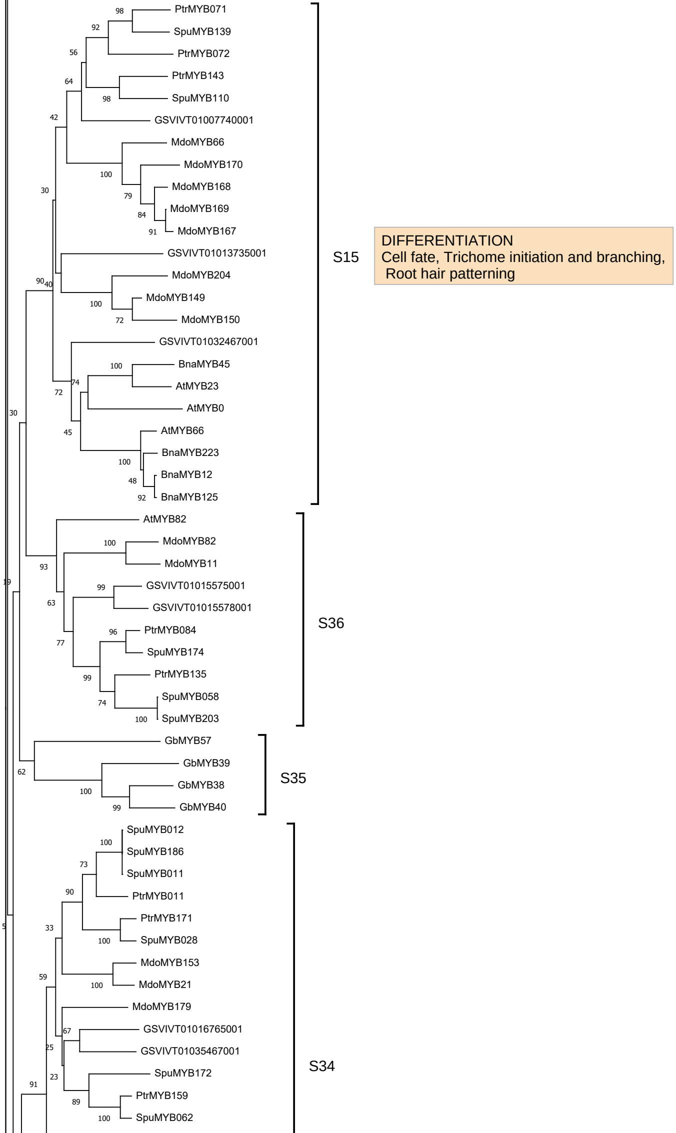
PtrMYB071
SpuMYB139
PtrMYB072
PtrMYB143
SpuMYB110
GSVIVT01007740001
MdoMYB66
MdoMYB170
MdoMYB168
MdoMYB169
MdoMYB167
GSVIVT01013735001
MdoMYB204
MdoMYB149
MdoMYB150
GSVIVT01032467001
BnaMYB45
AtMYB23
AtMYB0
AtMYB66
BnaMYB223
BnaMYB12
BnaMYB125
S15
DIFFERENTIATION
Cell fate, Trichome initiation and branching,
Root hair patterning
AtMYB82
MdoMYB82
MdoMYB11
GSVIVT01015575001
GSVIVT01015578001
PtrMYB084
SpuMYB174
PtrMYB135
SpuMYB058
SpuMYB203
S36
GbMYB57
GbMYB39
GbMYB38
GbMYB40
S35
SpuMYB012
SpuMYB186
SpuMYB011
PtrMYB011
PtrMYB171
SpuMYB028
MdoMYB153
MdoMYB21
MdoMYB179
GSVIVT01016765001
GSVIVT01035467001
SpuMYB172
PtrMYB159
SpuMYB062
S34

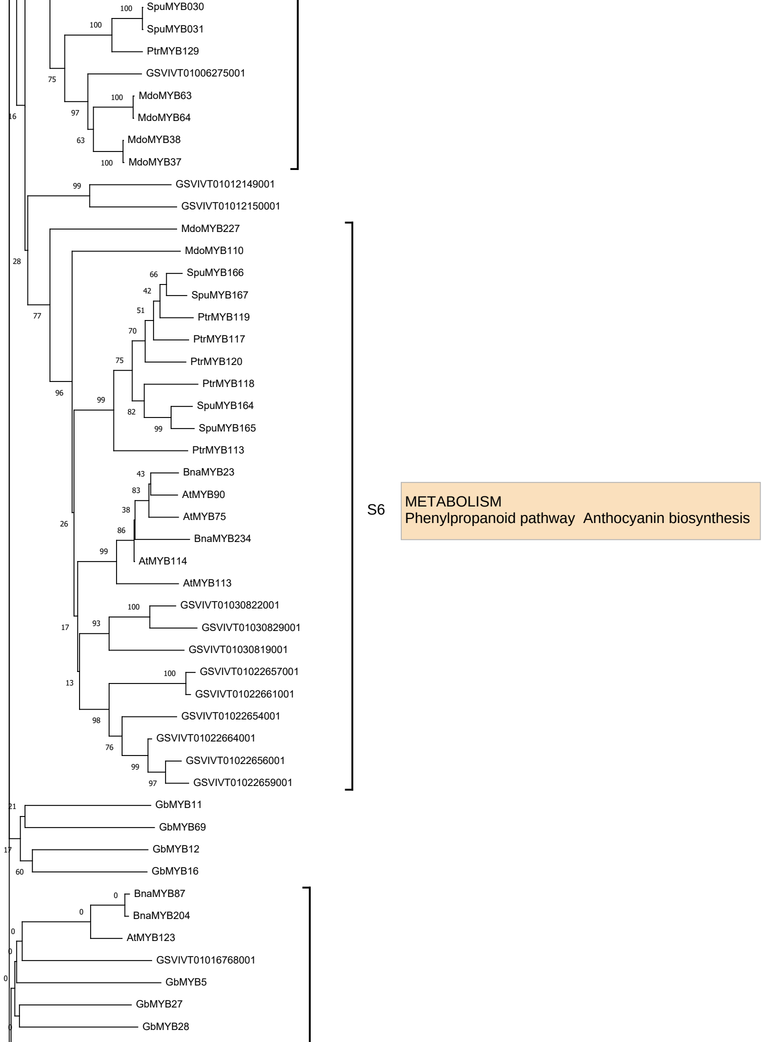
SpuMYB030
SpuMYB031
PtrMYB129
GSVIVT01006275001
MdoMYB63
MdoMYB64
MdoMYB38
MdoMYB37
GSVIVT01012149001
GSVIVT01012150001
MdoMYB227
MdoMYB110
SpuMYB166
SpuMYB167
PtrMYB119
PtrMYB117
PtrMYB120
PtrMYB118
SpuMYB164
SpuMYB165
PtrMYB113
BnaMYB23
AtMYB90
AtMYB75
BnaMYB234
AtMYB114
AtMYB113
GSVIVT01030822001
GSVIVT01030829001
GSVIVT01030819001
GSVIVT01022657001
GSVIVT01022661001
GSVIVT01022654001
GSVIVT01022664001
GSVIVT01022656001
GSVIVT01022659001
GbMYB11
GbMYB69
GbMYB12
GbMYB16
BnaMYB87
BnaMYB204
AtMYB123
GSVIVT01016768001
GbMYB5
GbMYB27
GbMYB28
S6
METABOLISM
Phenylpropanoid pathway Anthocyanin biosynthesis

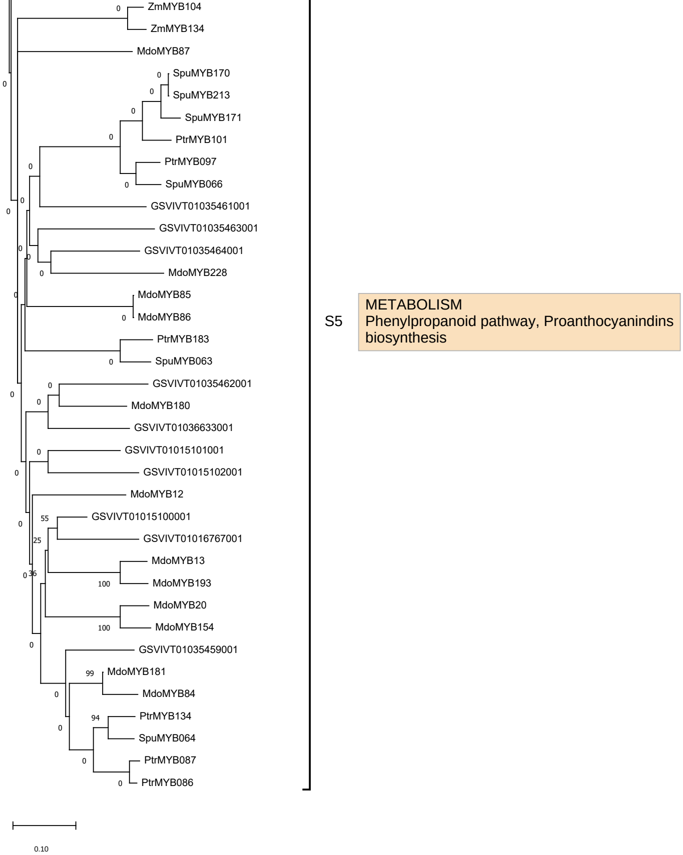

ZmMYB104
ZmMYB134
MdoMYB87
SpuMYB170
SpuMYB213
SpuMYB171
PtrMYB101
PtrMYB097
SpuMYB066
GSVIVT01035461001
GSVIVT01035463001
GSVIVT01035464001
MdoMYB228
MdoMYB85
MdoMYB86
PtrMYB183
SpuMYB063
GSVIVT01035462001
MdoMYB180
GSVIVT01036633001
GSVIVT01015101001
GSVIVT01015102001
MdoMYB12
GSVIVT01015100001
GSVIVT01016767001
MdoMYB13
MdoMYB193
MdoMYB20
MdoMYB154
GSVIVT01035459001
MdoMYB181
MdoMYB84
PtrMYB134
SpuMYB064
PtrMYB087
PtrMYB086
0.10
S5
METABOLISM
Phenylpropanoid pathway, Proanthocyanindins biosynthesis
