# Supplementary material for: Genome-Wide Comparative Analysis of R2R3 MYB Gene Family in Populus and Salix and Identification of Male Flower Bud Development-Related Genes
Source: Front Plant Sci. 2021 Sep 14;12:721558. doi: 10.3389/fpls.2021.721558 (PMC8477045; doi:10.3389/fpls.2021.721558)
Supplement: Supplementary Figure 1 — Maximum likelihood phylogenetic tree of poplar and willow R2R3 MYB genes. [file Supplementary Figure 5.pdf › Supplementary/Supplementary Figure 5.pdf]

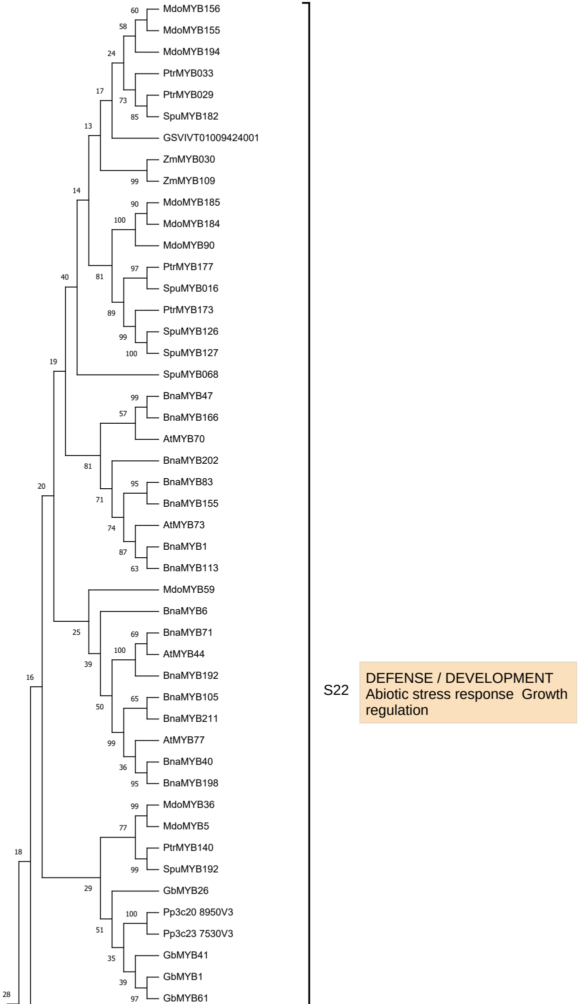
MdoMYB156
MdoMYB155
MdoMYB194
PtrMYB033
PtrMYB029
SpuMYB182
GSVIVT01009424001
ZmMYB030
ZmMYB109
MdoMYB185
MdoMYB184
MdoMYB90
PtrMYB177
SpuMYB016
PtrMYB173
SpuMYB126
SpuMYB127
SpuMYB068
BnaMYB47
BnaMYB166
AtMYB70
BnaMYB202
BnaMYB83
BnaMYB155
AtMYB73
BnaMYB1
BnaMYB113
MdoMYB59
BnaMYB6
BnaMYB71
AtMYB44
BnaMYB192
BnaMYB105
BnaMYB211
AtMYB77
BnaMYB40
BnaMYB198
MdoMYB36
MdoMYB5
PtrMYB140
SpuMYB192
GbMYB26
Pp3c20 8950V3
Pp3c23 7530V3
GbMYB41
GbMYB1
GbMYB61
S22
DEFENSE / DEVELOPMENT
Abiotic stress response Growth regulation

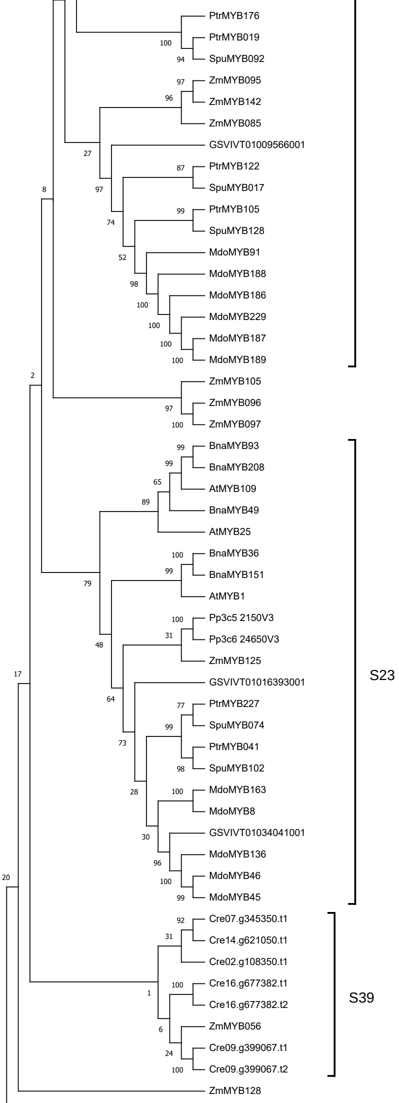
PtrMYB176
PtrMYB019
SpuMYB092
ZmMYB095
ZmMYB142
ZmMYB085
GSVIVT01009566001
PtrMYB122
SpuMYB017
PtrMYB105
SpuMYB128
MdoMYB91
MdoMYB188
MdoMYB186
MdoMYB229
MdoMYB187
MdoMYB189
ZmMYB105
ZmMYB096
ZmMYB097
BnaMYB93
BnaMYB208
AtMYB109
BnaMYB49
AtMYB25
BnaMYB36
BnaMYB151
AtMYB1
Pp3c5 2150V3
Pp3c6 24650V3
ZmMYB125
GSVIVT01016393001
PtrMYB227
SpuMYB074
PtrMYB041
SpuMYB102
MdoMYB163
MdoMYB8
GSVIVT01034041001
MdoMYB136
MdoMYB46
MdoMYB45
S23
Cre07.g345350.t1
Cre14.g621050.t1
Cre02.g108350.t1
Cre16.g677382.t1
Cre16.g677382.t2
ZmMYB056
Cre09.g399067.t1
Cre09.g399067.t2
S39
ZmMYB128

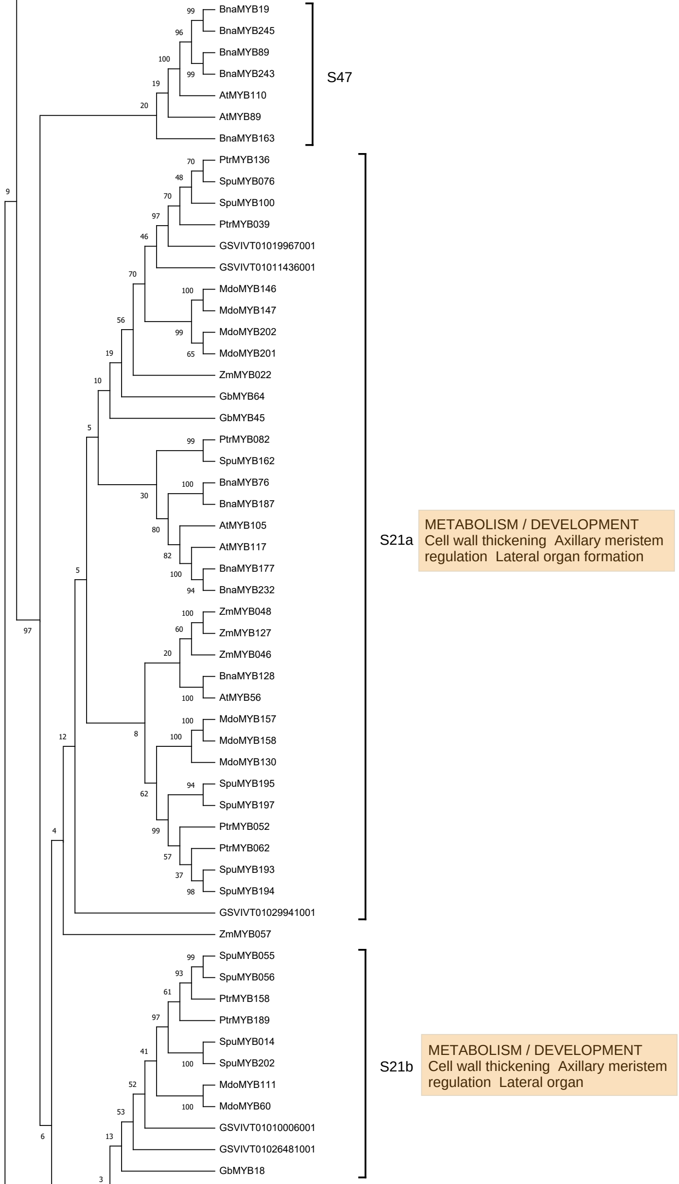
BnaMYB19
BnaMYB245
BnaMYB89
BnaMYB243
AtMYB110
AtMYB89
BnaMYB163
S47
PtrMYB136
SpuMYB076
SpuMYB100
PtrMYB039
GSVIVT01019967001
GSVIVT01011436001
MdoMYB146
MdoMYB147
MdoMYB202
MdoMYB201
ZmMYB022
GbMYB64
GbMYB45
PtrMYB082
SpuMYB162
BnaMYB76
BnaMYB187
AtMYB105
AtMYB117
BnaMYB177
BnaMYB232
ZmMYB048
ZmMYB127
ZmMYB046
BnaMYB128
AtMYB56
MdoMYB157
MdoMYB158
MdoMYB130
SpuMYB195
SpuMYB197
PtrMYB052
PtrMYB062
SpuMYB193
SpuMYB194
GSVIVT01029941001
S21a
METABOLISM / DEVELOPMENT
Cell wall thickening Axillary meristem regulation Lateral organ formation
ZmMYB057
SpuMYB055
SpuMYB056
PtrMYB158
PtrMYB189
SpuMYB014
SpuMYB202
MdoMYB111
MdoMYB60
GSVIVT01010006001
GSVIVT01026481001
GbMYB18
S21b
METABOLISM / DEVELOPMENT
Cell wall thickening Axillary meristem regulation Lateral organ

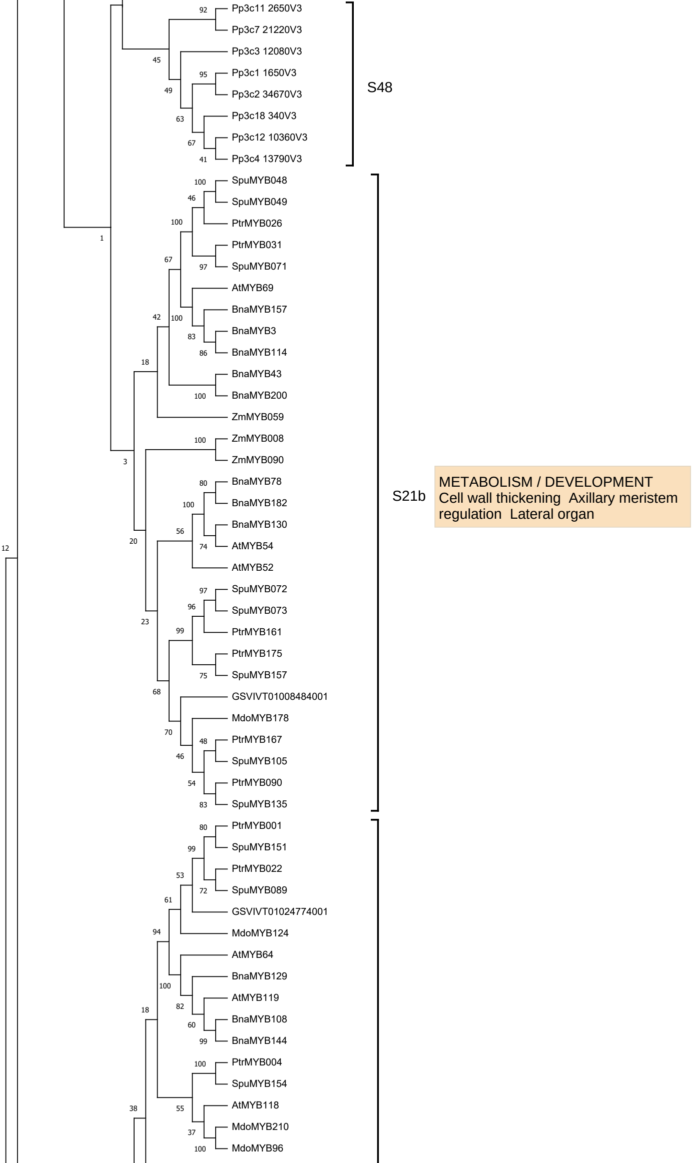
Pp3c11 2650V3
Pp3c7 21220V3
Pp3c3 12080V3
Pp3c1 1650V3
Pp3c2 34670V3
Pp3c18 340V3
Pp3c12 10360V3
Pp3c4 13790V3
S48
SpuMYB048
SpuMYB049
PtrMYB026
PtrMYB031
SpuMYB071
AtMYB69
BnaMYB157
BnaMYB3
BnaMYB114
BnaMYB43
BnaMYB200
ZmMYB059
ZmMYB008
ZmMYB090
BnaMYB78
BnaMYB182
BnaMYB130
AtMYB54
AtMYB52
SpuMYB072
SpuMYB073
PtrMYB161
PtrMYB175
SpuMYB157
GSVIVT01008484001
MdoMYB178
PtrMYB167
SpuMYB105
PtrMYB090
SpuMYB135
S21b
METABOLISM / DEVELOPMENT
Cell wall thickening Axillary meristem regulation Lateral organ
PtrMYB001
SpuMYB151
PtrMYB022
SpuMYB089
GSVIVT01024774001
MdoMYB124
AtMYB64
BnaMYB129
AtMYB119
BnaMYB108
BnaMYB144
PtrMYB004
SpuMYB154
AtMYB118
MdoMYB210
MdoMYB96

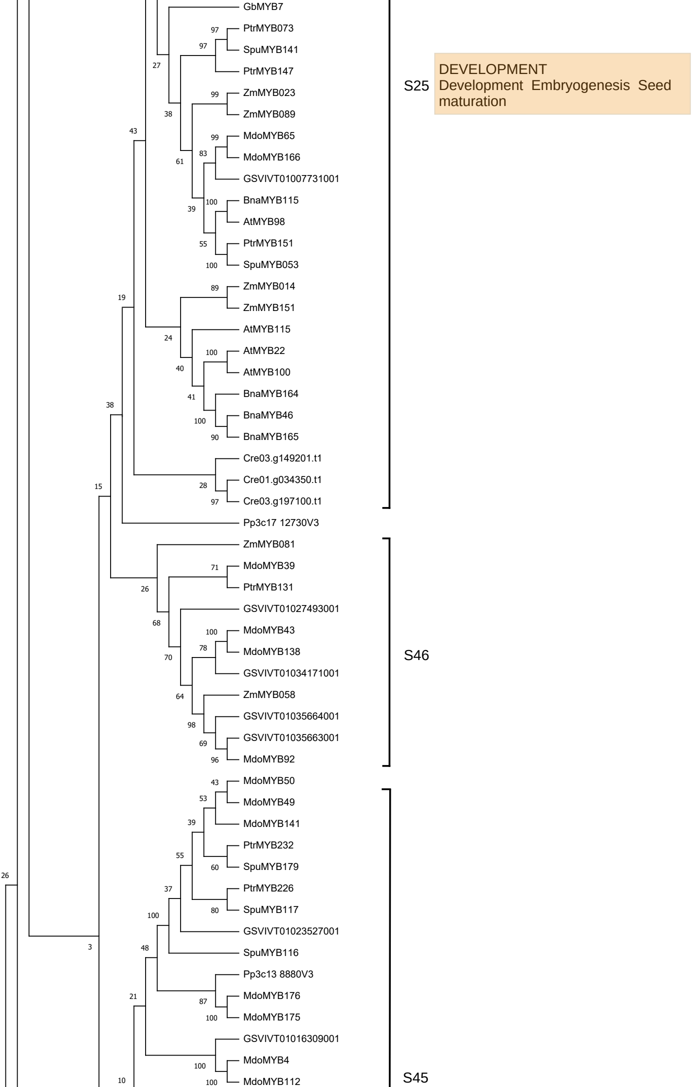
GbMYB7
PtrMYB073
SpuMYB141
PtrMYB147
ZmMYB023
ZmMYB089
MdoMYB65
MdoMYB166
GSVIVT01007731001
BnaMYB115
AtMYB98
PtrMYB151
SpuMYB053
ZmMYB014
ZmMYB151
AtMYB115
AtMYB22
AtMYB100
BnaMYB164
BnaMYB46
BnaMYB165
Cre03.g149201.t1
Cre01.g034350.t1
Cre03.g197100.t1
Pp3c17 12730V3
ZmMYB081
MdoMYB39
PtrMYB131
GSVIVT01027493001
MdoMYB43
MdoMYB138
GSVIVT01034171001
ZmMYB058
GSVIVT01035664001
GSVIVT01035663001
MdoMYB92
MdoMYB50
MdoMYB49
MdoMYB141
PtrMYB232
SpuMYB179
PtrMYB226
SpuMYB117
GSVIVT01023527001
SpuMYB116
Pp3c13 8880V3
MdoMYB176
MdoMYB175
GSVIVT01016309001
MdoMYB4
MdoMYB112
S25
DEVELOPMENT
Development Embryogenesis Seed maturation
S46
S45

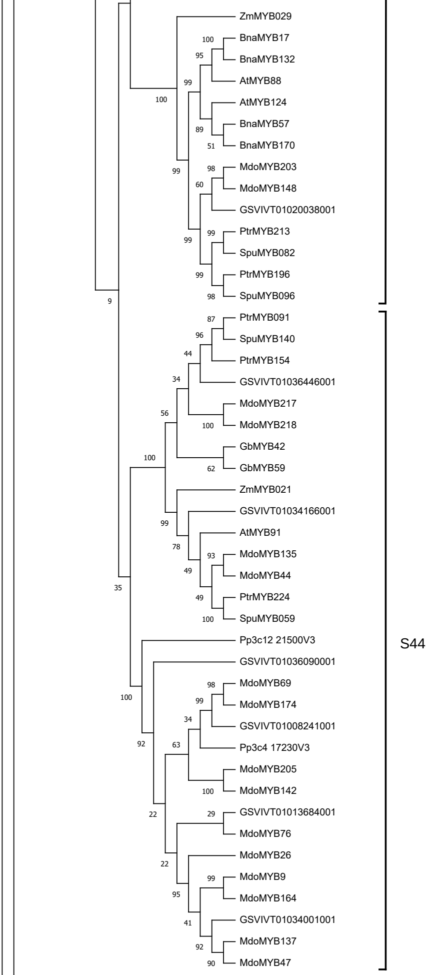
ZmMYB029
BnaMYB17
BnaMYB132
AtMYB88
AtMYB124
BnaMYB57
BnaMYB170
MdoMYB203
MdoMYB148
GSVIVT01020038001
PtrMYB213
SpuMYB082
PtrMYB196
SpuMYB096
PtrMYB091
SpuMYB140
PtrMYB154
GSVIVT01036446001
MdoMYB217
MdoMYB218
GbMYB42
GbMYB59
ZmMYB021
GSVIVT01034166001
AtMYB91
MdoMYB135
MdoMYB44
PtrMYB224
SpuMYB059
Pp3c12 21500V3
GSVIVT01036090001
MdoMYB69
MdoMYB174
GSVIVT01008241001
Pp3c4 17230V3
MdoMYB205
MdoMYB142
GSVIVT01013684001
MdoMYB76
MdoMYB26
MdoMYB9
MdoMYB164
GSVIVT01034001001
MdoMYB137
MdoMYB47
S44

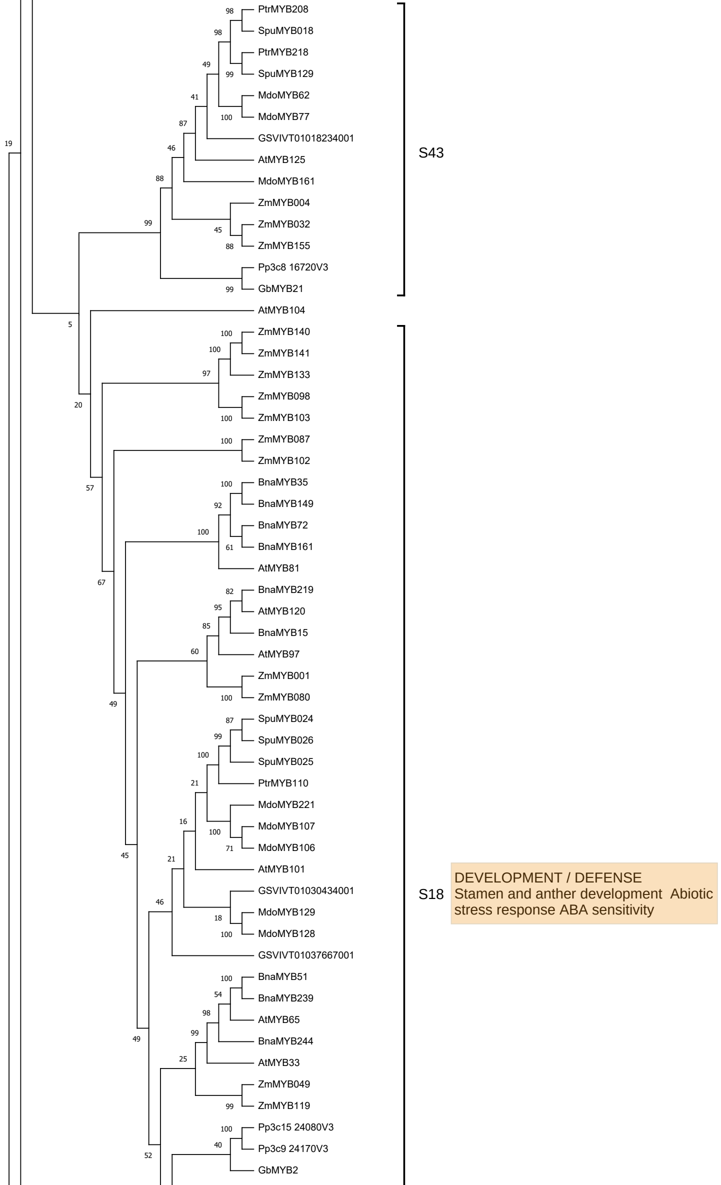
PtrMYB208
SpuMYB018
PtrMYB218
SpuMYB129
MdoMYB62
MdoMYB77
GSVIVT01018234001
AtMYB125
MdoMYB161
ZmMYB004
ZmMYB032
ZmMYB155
Pp3c8 16720V3
GbMYB21
S43
AtMYB104
ZmMYB140
ZmMYB141
ZmMYB133
ZmMYB098
ZmMYB103
ZmMYB087
ZmMYB102
BnaMYB35
BnaMYB149
BnaMYB72
BnaMYB161
AtMYB81
BnaMYB219
AtMYB120
BnaMYB15
AtMYB97
ZmMYB001
ZmMYB080
SpuMYB024
SpuMYB026
SpuMYB025
PtrMYB110
MdoMYB221
MdoMYB107
MdoMYB106
AtMYB101
GSVIVT01030434001
MdoMYB129
MdoMYB128
GSVIVT01037667001
BnaMYB51
BnaMYB239
AtMYB65
BnaMYB244
AtMYB33
ZmMYB049
ZmMYB119
Pp3c15 24080V3
Pp3c9 24170V3
GbMYB2
S18
DEVELOPMENT / DEFENSE
Stamen and anther development Abiotic stress response ABA sensitivity

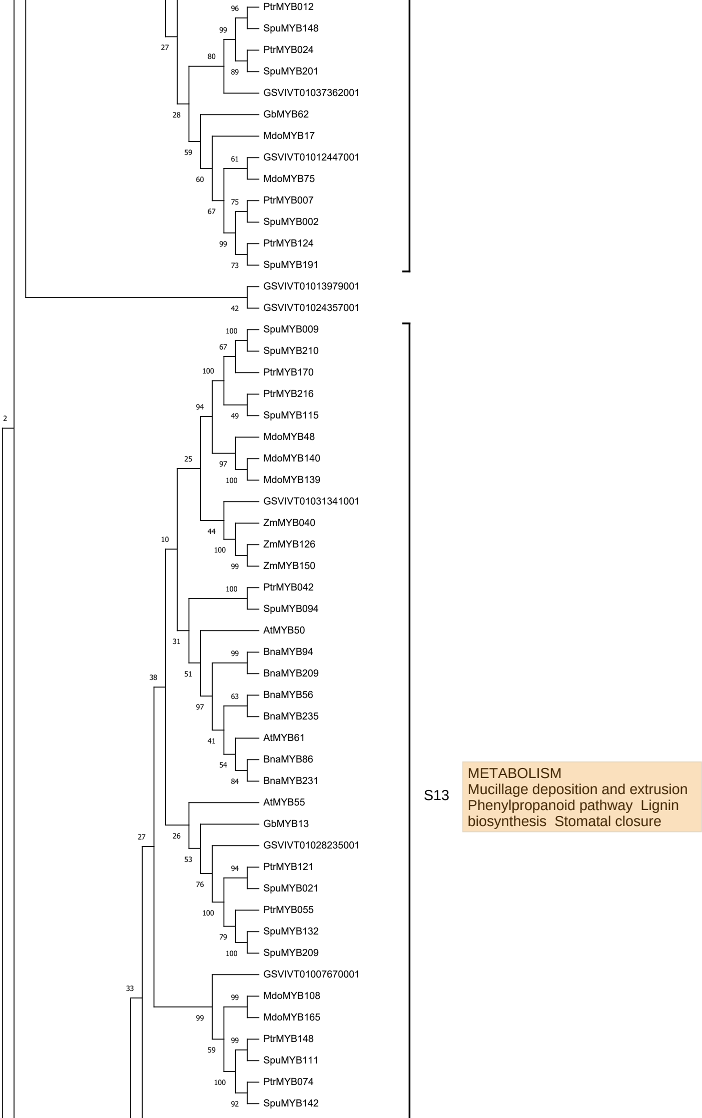
PtrMYB012
SpuMYB148
PtrMYB024
SpuMYB201
GSVIVT01037362001
GbMYB62
MdoMYB17
GSVIVT01012447001
MdoMYB75
PtrMYB007
SpuMYB002
PtrMYB124
SpuMYB191
GSVIVT01013979001
GSVIVT01024357001
SpuMYB009
SpuMYB210
PtrMYB170
PtrMYB216
SpuMYB115
MdoMYB48
MdoMYB140
MdoMYB139
GSVIVT01031341001
ZmMYB040
ZmMYB126
ZmMYB150
PtrMYB042
SpuMYB094
AtMYB50
BnaMYB94
BnaMYB209
BnaMYB56
BnaMYB235
AtMYB61
BnaMYB86
BnaMYB231
AtMYB55
GbMYB13
GSVIVT01028235001
PtrMYB121
SpuMYB021
PtrMYB055
SpuMYB132
SpuMYB209
GSVIVT01007670001
MdoMYB108
MdoMYB165
PtrMYB148
SpuMYB111
PtrMYB074
SpuMYB142
S13
METABOLISM
Mucillage deposition and extrusion
Phenylpropanoid pathway Lignin
biosynthesis Stomatal closure

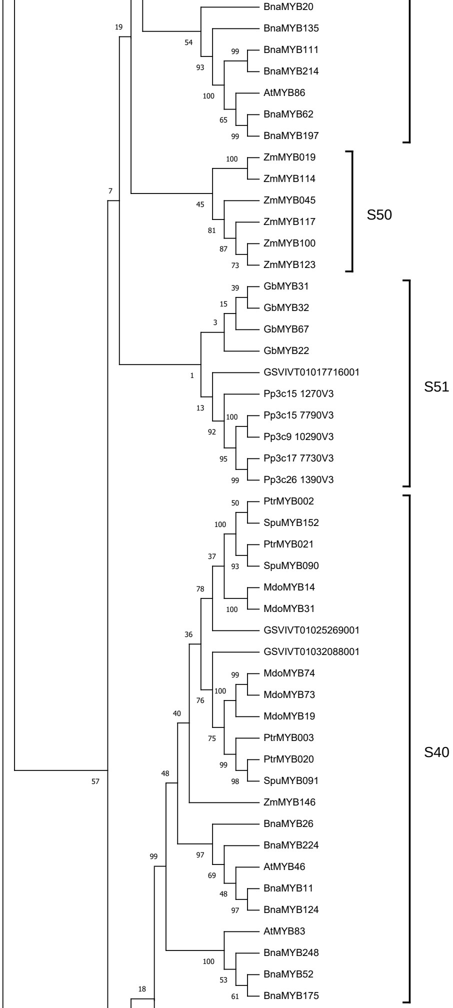
BnaMYB20
BnaMYB135
BnaMYB111
BnaMYB214
AtMYB86
BnaMYB62
BnaMYB197
ZmMYB019
ZmMYB114
ZmMYB045
ZmMYB117
ZmMYB100
ZmMYB123
S50
GbMYB31
GbMYB32
GbMYB67
GbMYB22
GSVIVT01017716001
Pp3c15 1270V3
Pp3c15 7790V3
Pp3c9 10290V3
Pp3c17 7730V3
Pp3c26 1390V3
S51
PtrMYB002
SpuMYB152
PtrMYB021
SpuMYB090
MdoMYB14
MdoMYB31
GSVIVT01025269001
GSVIVT01032088001
MdoMYB74
MdoMYB73
MdoMYB19
PtrMYB003
PtrMYB020
SpuMYB091
ZmMYB146
BnaMYB26
BnaMYB224
AtMYB46
BnaMYB11
BnaMYB124
AtMYB83
BnaMYB248
BnaMYB52
BnaMYB175
S40

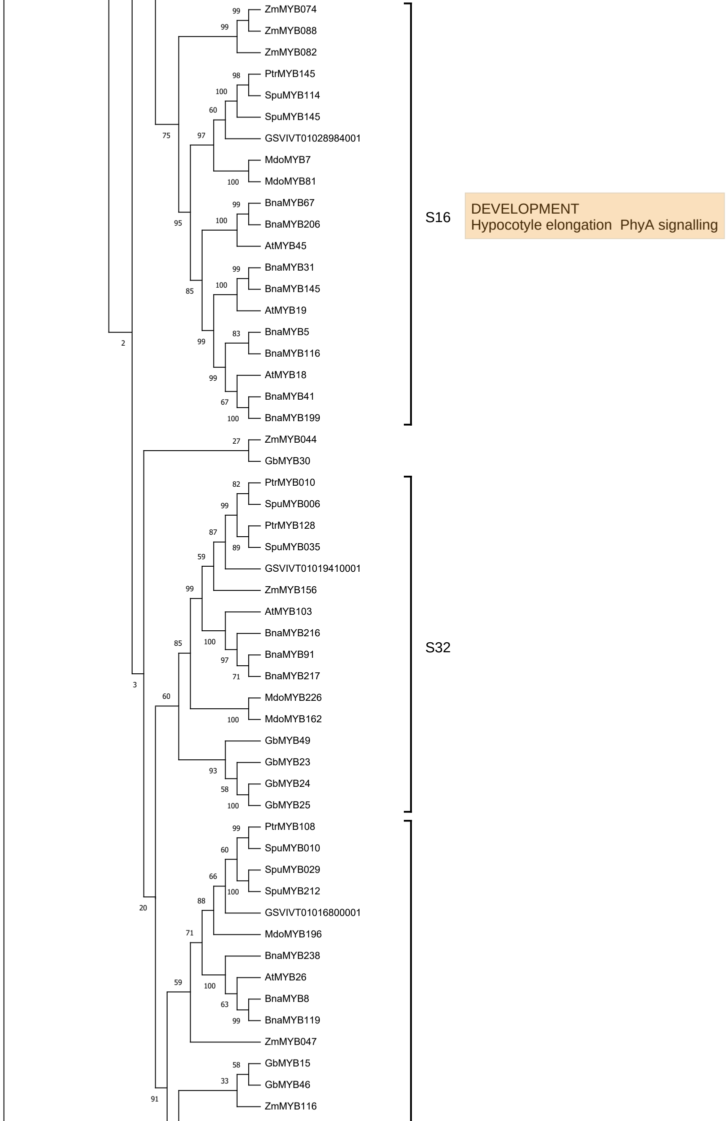

ZmMYB074
ZmMYB088
ZmMYB082
PtrMYB145
SpuMYB114
SpuMYB145
GSVIVT01028984001
MdoMYB7
MdoMYB81
BnaMYB67
BnaMYB206
AtMYB45
BnaMYB31
BnaMYB145
AtMYB19
BnaMYB5
BnaMYB116
AtMYB18
BnaMYB41
BnaMYB199
ZmMYB044
GbMYB30
PtrMYB010
SpuMYB006
PtrMYB128
SpuMYB035
GSVIVT01019410001
ZmMYB156
AtMYB103
BnaMYB216
BnaMYB91
BnaMYB217
MdoMYB226
MdoMYB162
GbMYB49
GbMYB23
GbMYB24
GbMYB25
PtrMYB108
SpuMYB010
SpuMYB029
SpuMYB212
GSVIVT01016800001
MdoMYB196
BnaMYB238
AtMYB26
BnaMYB8
BnaMYB119
ZmMYB047
GbMYB15
GbMYB46
ZmMYB116
S16
DEVELOPMENT
Hypocotyle elongation PhyA signalling
S32

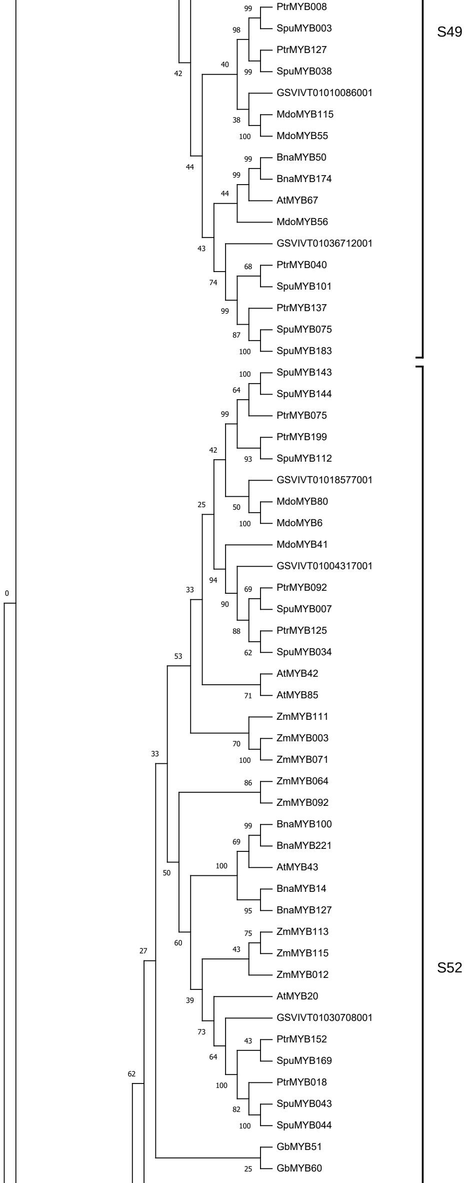
PtrMYB008
SpuMYB003
PtrMYB127
SpuMYB038
GSVIVT01010086001
MdoMYB115
MdoMYB55
BnaMYB50
BnaMYB174
AtMYB67
MdoMYB56
GSVIVT01036712001
PtrMYB040
SpuMYB101
PtrMYB137
SpuMYB075
SpuMYB183
S49
SpuMYB143
SpuMYB144
PtrMYB075
PtrMYB199
SpuMYB112
GSVIVT01018577001
MdoMYB80
MdoMYB6
MdoMYB41
GSVIVT01004317001
PtrMYB092
SpuMYB007
PtrMYB125
SpuMYB034
AtMYB42
AtMYB85
ZmMYB111
ZmMYB003
ZmMYB071
ZmMYB064
ZmMYB092
BnaMYB100
BnaMYB221
AtMYB43
BnaMYB14
BnaMYB127
ZmMYB113
ZmMYB115
ZmMYB012
AtMYB20
GSVIVT01030708001
PtrMYB152
SpuMYB169
PtrMYB018
SpuMYB043
SpuMYB044
GbMYB51
GbMYB60
S52

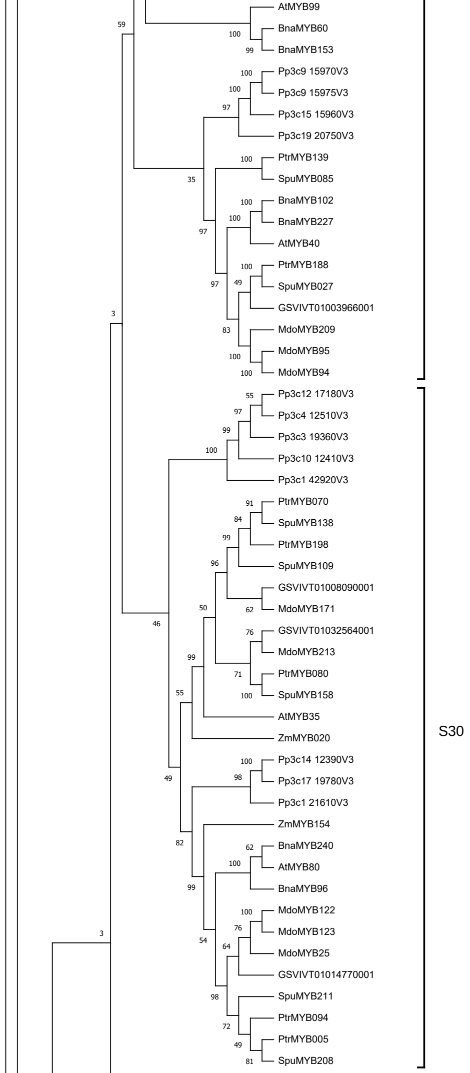
AtMYB99
BnaMYB60
BnaMYB153
Pp3c9 15970V3
Pp3c9 15975V3
Pp3c15 15960V3
Pp3c19 20750V3
PtrMYB139
SpuMYB085
BnaMYB102
BnaMYB227
AtMYB40
PtrMYB188
SpuMYB027
GSVIVT01003966001
MdoMYB209
MdoMYB95
MdoMYB94
Pp3c12 17180V3
Pp3c4 12510V3
Pp3c3 19360V3
Pp3c10 12410V3
Pp3c1 42920V3
PtrMYB070
SpuMYB138
PtrMYB198
SpuMYB109
GSVIVT01008090001
MdoMYB171
GSVIVT01032564001
MdoMYB213
PtrMYB080
SpuMYB158
AtMYB35
ZmMYB020
Pp3c14 12390V3
Pp3c17 19780V3
Pp3c1 21610V3
ZmMYB154
BnaMYB240
AtMYB80
BnaMYB96
MdoMYB122
MdoMYB123
MdoMYB25
GSVIVT01014770001
SpuMYB211
PtrMYB094
PtrMYB005
SpuMYB208
S30

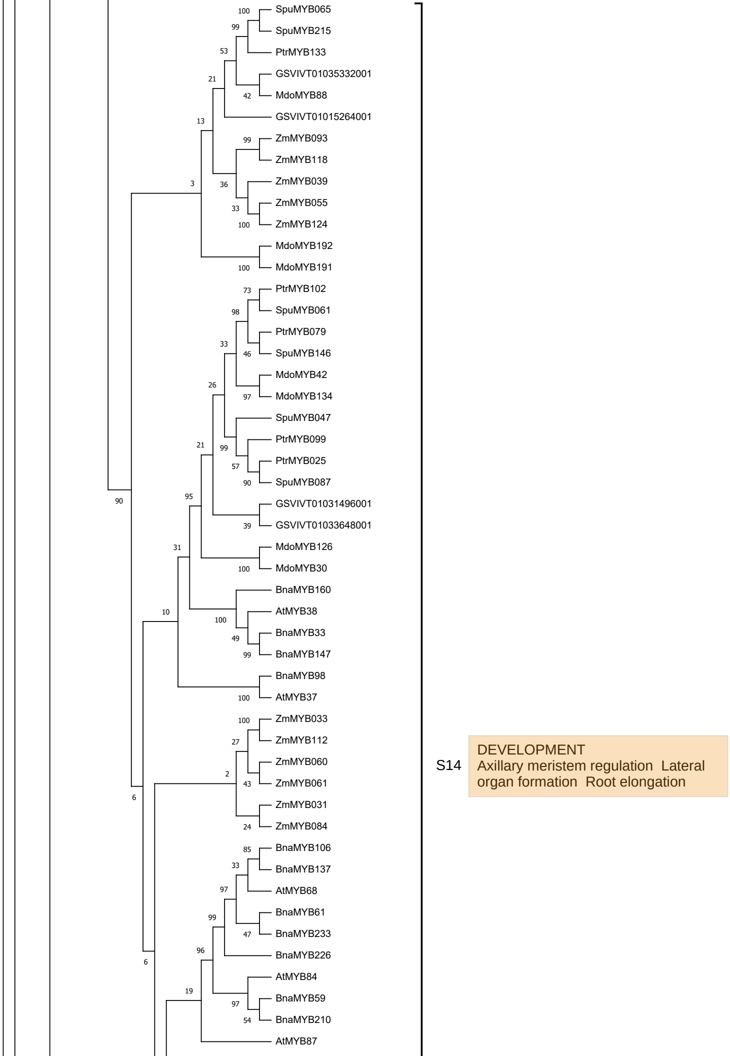
SpuMYB065
SpuMYB215
PtrMYB133
GSVIVT01035332001
MdoMYB88
GSVIVT01015264001
ZmMYB093
ZmMYB118
ZmMYB039
ZmMYB055
ZmMYB124
MdoMYB192
MdoMYB191
PtrMYB102
SpuMYB061
PtrMYB079
SpuMYB146
MdoMYB42
MdoMYB134
SpuMYB047
PtrMYB099
PtrMYB025
SpuMYB087
GSVIVT01031496001
GSVIVT01033648001
MdoMYB126
MdoMYB30
BnaMYB160
AtMYB38
BnaMYB33
BnaMYB147
BnaMYB98
AtMYB37
ZmMYB033
ZmMYB112
ZmMYB060
ZmMYB061
ZmMYB031
ZmMYB084
BnaMYB106
BnaMYB137
AtMYB68
BnaMYB61
BnaMYB233
BnaMYB226
AtMYB84
BnaMYB59
BnaMYB210
AtMYB87
S14
DEVELOPMENT
Axillary meristem regulation Lateral organ formation Root elongation

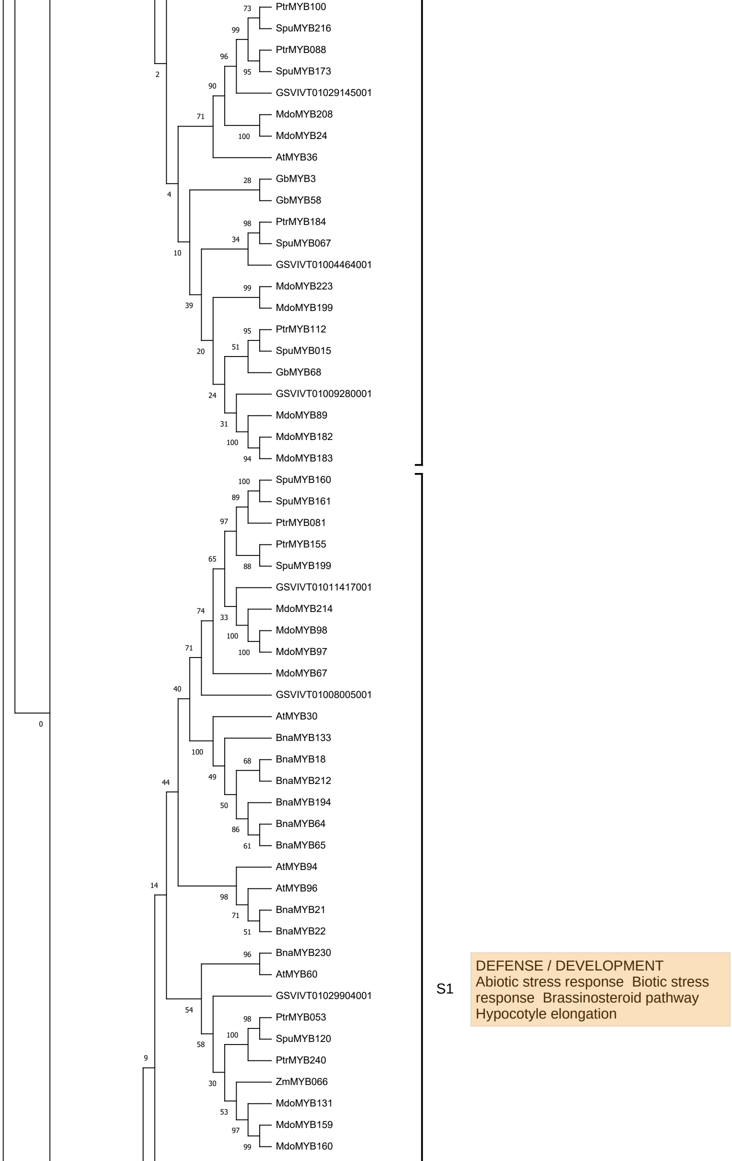

PtrMYB100
SpuMYB216
PtrMYB088
SpuMYB173
GSVIVT01029145001
MdoMYB208
MdoMYB24
AtMYB36
GbMYB3
GbMYB58
PtrMYB184
SpuMYB067
GSVIVT01004464001
MdoMYB223
MdoMYB199
PtrMYB112
SpuMYB015
GbMYB68
GSVIVT01009280001
MdoMYB89
MdoMYB182
MdoMYB183
SpuMYB160
SpuMYB161
PtrMYB081
PtrMYB155
SpuMYB199
GSVIVT01011417001
MdoMYB214
MdoMYB98
MdoMYB97
MdoMYB67
GSVIVT01008005001
AtMYB30
BnaMYB133
BnaMYB18
BnaMYB212
BnaMYB194
BnaMYB64
BnaMYB65
AtMYB94
AtMYB96
BnaMYB21
BnaMYB22
BnaMYB230
AtMYB60
GSVIVT01029904001
PtrMYB053
SpuMYB120
PtrMYB240
ZmMYB066
MdoMYB131
MdoMYB159
MdoMYB160
S1
DEFENSE / DEVELOPMENT
Abiotic stress response Biotic stress response Brassinosteroid pathway Hypocotyle elongation

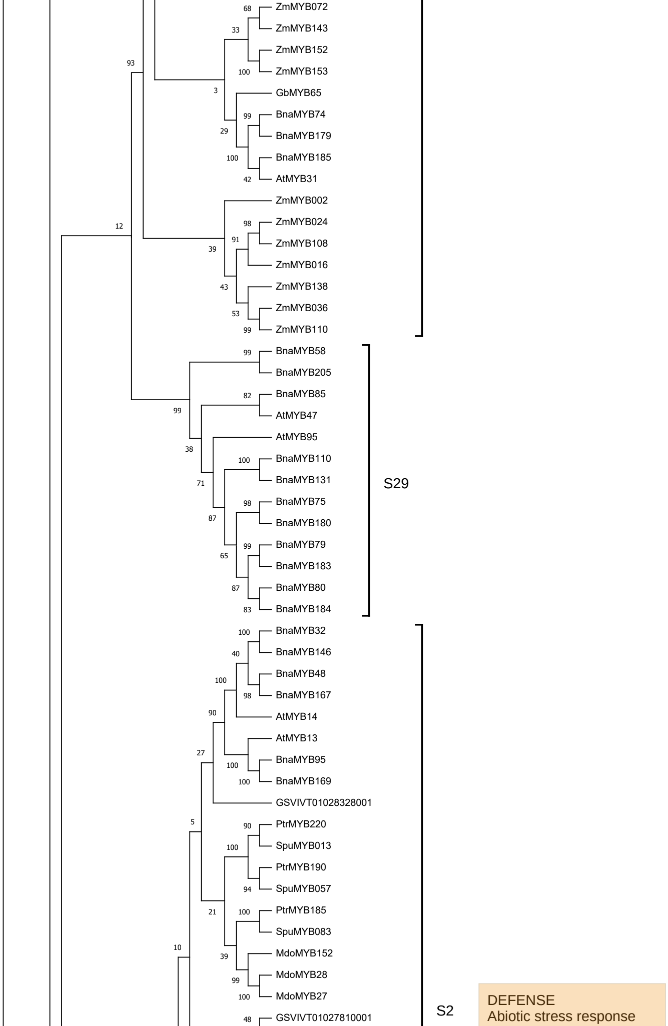

ZmMYB072
ZmMYB143
ZmMYB152
ZmMYB153
GbMYB65
BnaMYB74
BnaMYB179
BnaMYB185
AtMYB31
ZmMYB002
ZmMYB024
ZmMYB108
ZmMYB016
ZmMYB138
ZmMYB036
ZmMYB110
BnaMYB58
BnaMYB205
BnaMYB85
AtMYB47
AtMYB95
BnaMYB110
BnaMYB131
BnaMYB75
BnaMYB180
BnaMYB79
BnaMYB183
BnaMYB80
BnaMYB184
S29
BnaMYB32
BnaMYB146
BnaMYB48
BnaMYB167
AtMYB14
AtMYB13
BnaMYB95
BnaMYB169
GSVIVT01028328001
PtrMYB220
SpuMYB013
PtrMYB190
SpuMYB057
PtrMYB185
SpuMYB083
MdoMYB152
MdoMYB28
MdoMYB27
GSVIVT01027810001
S2
DEFENSE
Abiotic stress response

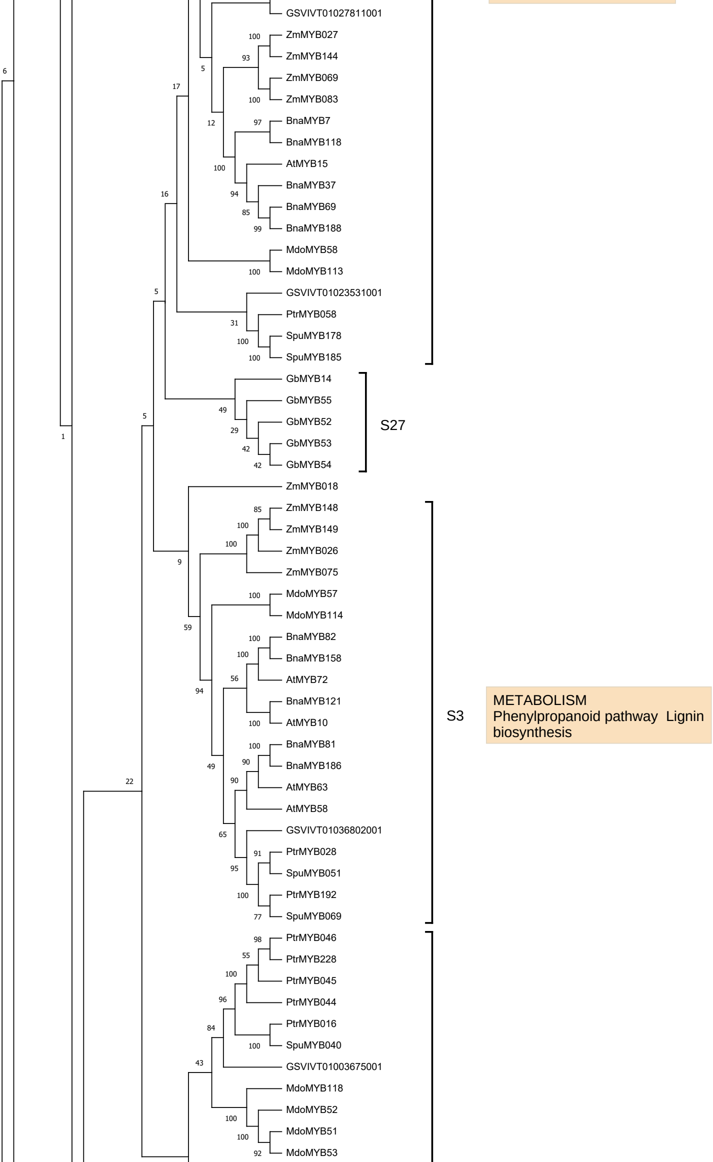

GSVIVT01027811001
ZmMYB027
ZmMYB144
ZmMYB069
ZmMYB083
BnaMYB7
BnaMYB118
AtMYB15
BnaMYB37
BnaMYB69
BnaMYB188
MdoMYB58
MdoMYB113
GSVIVT01023531001
PtrMYB058
SpuMYB178
SpuMYB185
GbMYB14
GbMYB55
GbMYB52
GbMYB53
GbMYB54
S27
ZmMYB018
ZmMYB148
ZmMYB149
ZmMYB026
ZmMYB075
MdoMYB57
MdoMYB114
BnaMYB82
BnaMYB158
AtMYB72
BnaMYB121
AtMYB10
BnaMYB81
BnaMYB186
AtMYB63
AtMYB58
GSVIVT01036802001
PtrMYB028
SpuMYB051
PtrMYB192
SpuMYB069
S3
METABOLISM
Phenylpropanoid pathway Lignin biosynthesis
PtrMYB046
PtrMYB228
PtrMYB045
PtrMYB044
PtrMYB016
SpuMYB040
GSVIVT01003675001
MdoMYB118
MdoMYB52
MdoMYB51
MdoMYB53

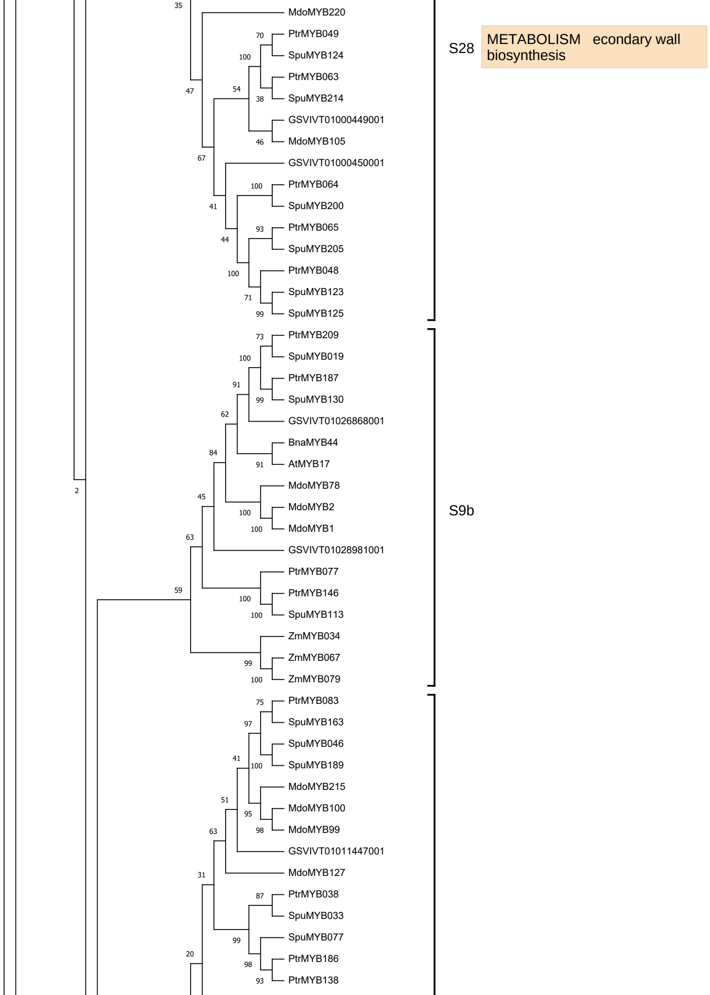
MdoMYB220
PtrMYB049
SpuMYB124
PtrMYB063
SpuMYB214
GSVIVT01000449001
MdoMYB105
GSVIVT01000450001
PtrMYB064
SpuMYB200
PtrMYB065
SpuMYB205
PtrMYB048
SpuMYB123
SpuMYB125
S28
METABOLISM econdary wall biosynthesis
PtrMYB209
SpuMYB019
PtrMYB187
SpuMYB130
GSVIVT01026868001
BnaMYB44
AtMYB17
MdoMYB78
MdoMYB2
MdoMYB1
GSVIVT01028981001
PtrMYB077
PtrMYB146
SpuMYB113
ZmMYB034
ZmMYB067
ZmMYB079
S9b
PtrMYB083
SpuMYB163
SpuMYB046
SpuMYB189
MdoMYB215
MdoMYB100
MdoMYB99
GSVIVT01011447001
MdoMYB127
PtrMYB038
SpuMYB033
SpuMYB077
PtrMYB186
PtrMYB138

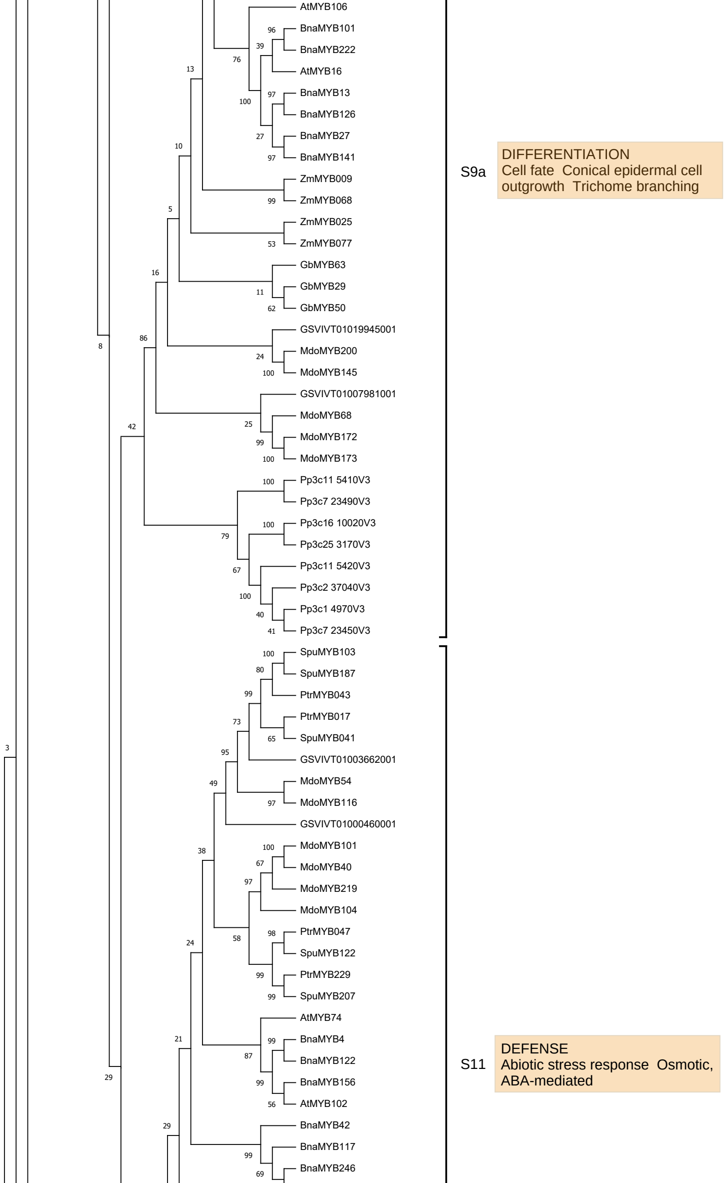
AtMYB106
BnaMYB101
BnaMYB222
AtMYB16
BnaMYB13
BnaMYB126
BnaMYB27
BnaMYB141
ZmMYB009
ZmMYB068
ZmMYB025
ZmMYB077
GbMYB63
GbMYB29
GbMYB50
GSVIVT01019945001
MdoMYB200
MdoMYB145
GSVIVT01007981001
MdoMYB68
MdoMYB172
MdoMYB173
Pp3c11 5410V3
Pp3c7 23490V3
Pp3c16 10020V3
Pp3c25 3170V3
Pp3c11 5420V3
Pp3c2 37040V3
Pp3c1 4970V3
Pp3c7 23450V3
S9a
DIFFERENTIATION
Cell fate Conical epidermal cell outgrowth Trichome branching
SpuMYB103
SpuMYB187
PtrMYB043
PtrMYB017
SpuMYB041
GSVIVT01003662001
MdoMYB54
MdoMYB116
GSVIVT01000460001
MdoMYB101
MdoMYB40
MdoMYB219
MdoMYB104
PtrMYB047
SpuMYB122
PtrMYB229
SpuMYB207
AtMYB74
BnaMYB4
BnaMYB122
BnaMYB156
AtMYB102
BnaMYB42
BnaMYB117
BnaMYB246
S11
DEFENSE
Abiotic stress response Osmotic, ABA-mediated

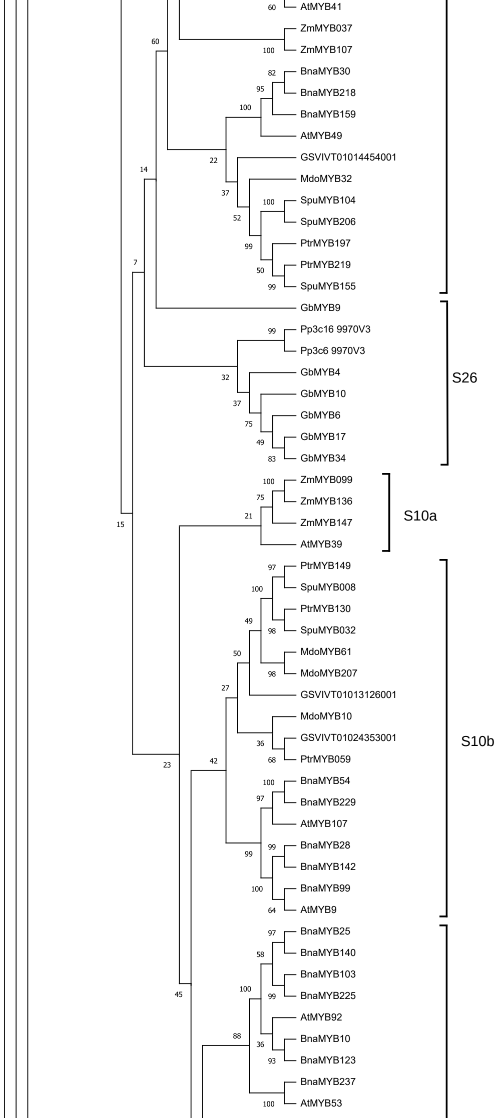

AtMYB41
ZmMYB037
ZmMYB107
BnaMYB30
BnaMYB218
BnaMYB159
AtMYB49
GSVIVT01014454001
MdoMYB32
SpuMYB104
SpuMYB206
PtrMYB197
PtrMYB219
SpuMYB155
GbMYB9
Pp3c16 9970V3
Pp3c6 9970V3
GbMYB4
GbMYB10
GbMYB6
GbMYB17
GbMYB34
S26
ZmMYB099
ZmMYB136
ZmMYB147
AtMYB39
S10a
PtrMYB149
SpuMYB008
PtrMYB130
SpuMYB032
MdoMYB61
MdoMYB207
GSVIVT01013126001
MdoMYB10
GSVIVT01024353001
PtrMYB059
BnaMYB54
BnaMYB229
AtMYB107
BnaMYB28
BnaMYB142
BnaMYB99
AtMYB9
S10b
BnaMYB25
BnaMYB140
BnaMYB103
BnaMYB225
AtMYB92
BnaMYB10
BnaMYB123
BnaMYB237
AtMYB53

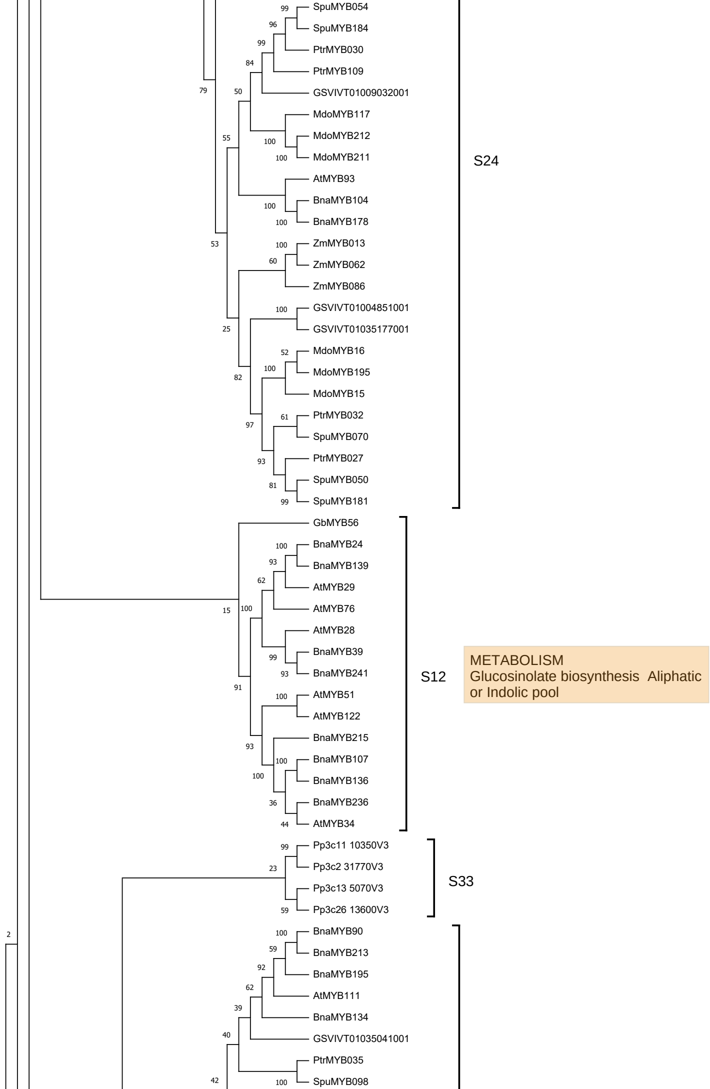
SpuMYB054
SpuMYB184
PtrMYB030
PtrMYB109
GSVIVT01009032001
MdoMYB117
MdoMYB212
MdoMYB211
AtMYB93
BnaMYB104
BnaMYB178
ZmMYB013
ZmMYB062
ZmMYB086
GSVIVT01004851001
GSVIVT01035177001
MdoMYB16
MdoMYB195
MdoMYB15
PtrMYB032
SpuMYB070
PtrMYB027
SpuMYB050
SpuMYB181
S24
GbMYB56
BnaMYB24
BnaMYB139
AtMYB29
AtMYB76
AtMYB28
BnaMYB39
BnaMYB241
AtMYB51
AtMYB122
BnaMYB215
BnaMYB107
BnaMYB136
BnaMYB236
AtMYB34
S12
METABOLISM
Glucosinolate biosynthesis Aliphatic or Indolic pool
Pp3c11 10350V3
Pp3c2 31770V3
Pp3c13 5070V3
Pp3c26 13600V3
S33
BnaMYB90
BnaMYB213
BnaMYB195
AtMYB111
BnaMYB134
GSVIVT01035041001
PtrMYB035
SpuMYB098

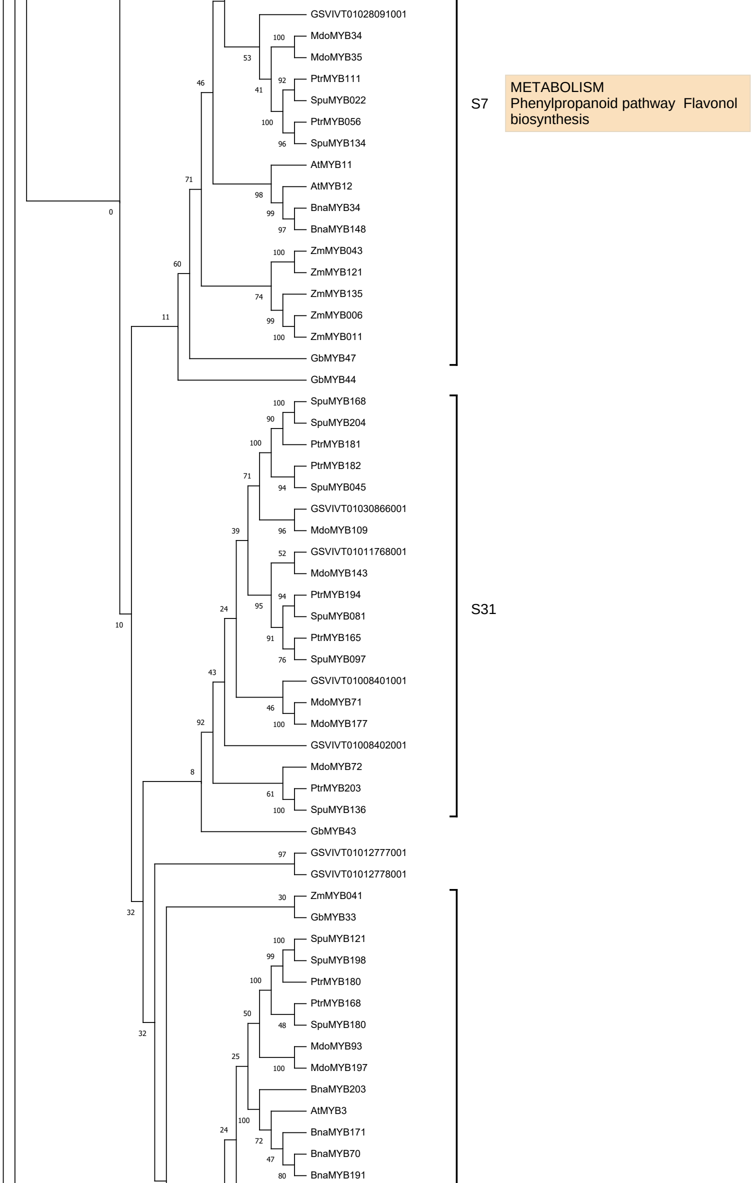
GSVIVT01028091001
MdoMYB34
MdoMYB35
PtrMYB111
SpuMYB022
PtrMYB056
SpuMYB134
AtMYB11
AtMYB12
BnaMYB34
BnaMYB148
ZmMYB043
ZmMYB121
ZmMYB135
ZmMYB006
ZmMYB011
GbMYB47
GbMYB44
S7
METABOLISM
Phenylpropanoid pathway Flavonol biosynthesis
SpuMYB168
SpuMYB204
PtrMYB181
PtrMYB182
SpuMYB045
GSVIVT01030866001
MdoMYB109
GSVIVT01011768001
MdoMYB143
PtrMYB194
SpuMYB081
PtrMYB165
SpuMYB097
GSVIVT01008401001
MdoMYB71
MdoMYB177
GSVIVT01008402001
MdoMYB72
PtrMYB203
SpuMYB136
S31
GbMYB43
GSVIVT01012777001
GSVIVT01012778001
ZmMYB041
GbMYB33
SpuMYB121
SpuMYB198
PtrMYB180
PtrMYB168
SpuMYB180
MdoMYB93
MdoMYB197
BnaMYB203
AtMYB3
BnaMYB171
BnaMYB70
BnaMYB191

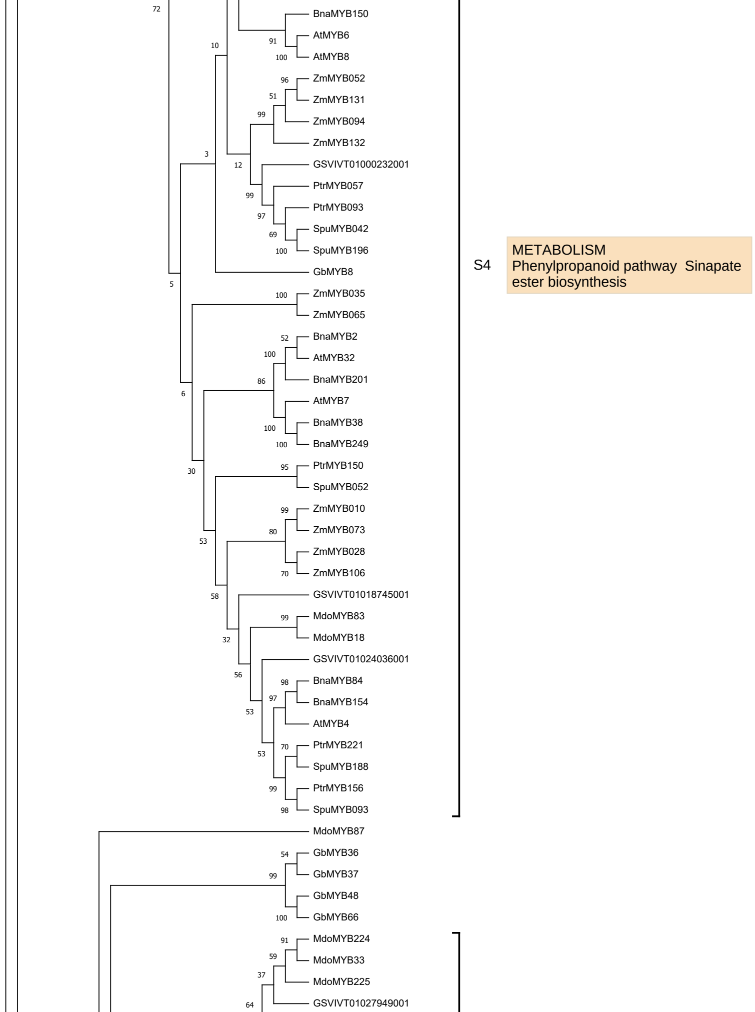

BnaMYB150
AtMYB6
AtMYB8
ZmMYB052
ZmMYB131
ZmMYB094
ZmMYB132
GSVIVT01000232001
PtrMYB057
PtrMYB093
SpuMYB042
SpuMYB196
GbMYB8
ZmMYB035
ZmMYB065
BnaMYB2
AtMYB32
BnaMYB201
AtMYB7
BnaMYB38
BnaMYB249
PtrMYB150
SpuMYB052
ZmMYB010
ZmMYB073
ZmMYB028
ZmMYB106
GSVIVT01018745001
MdoMYB83
MdoMYB18
GSVIVT01024036001
BnaMYB84
BnaMYB154
AtMYB4
PtrMYB221
SpuMYB188
PtrMYB156
SpuMYB093
MdoMYB87
GbMYB36
GbMYB37
GbMYB48
GbMYB66
MdoMYB224
MdoMYB33
MdoMYB225
GSVIVT01027949001
S4
METABOLISM
Phenylpropanoid pathway Sinapate ester biosynthesis

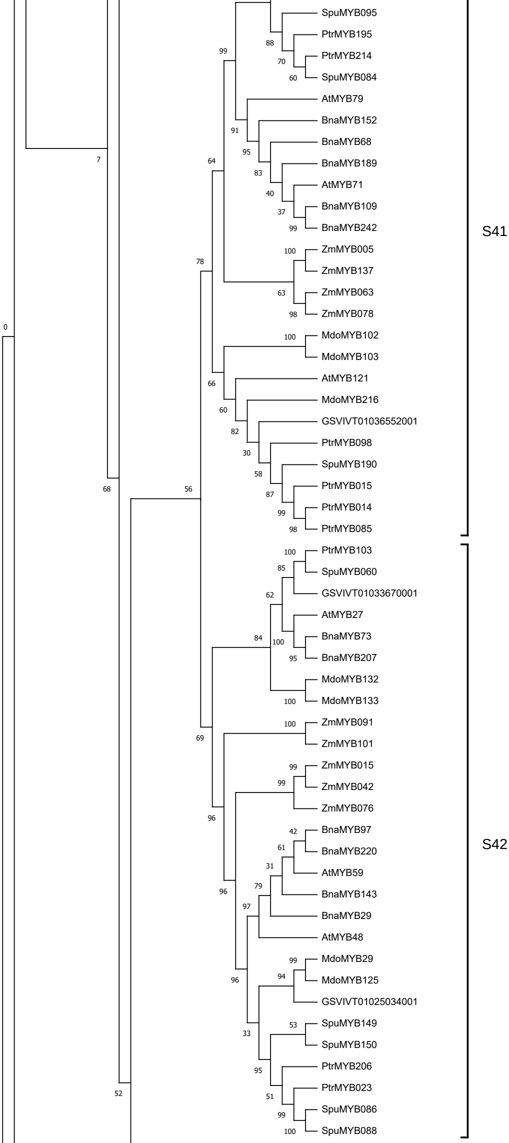
SpuMYB095
PtrMYB195
PtrMYB214
SpuMYB084
AtMYB79
BnaMYB152
BnaMYB68
BnaMYB189
AtMYB71
BnaMYB109
BnaMYB242
ZmMYB005
ZmMYB137
ZmMYB063
ZmMYB078
MdoMYB102
MdoMYB103
AtMYB121
MdoMYB216
GSVIVT01036552001
PtrMYB098
SpuMYB190
PtrMYB015
PtrMYB014
PtrMYB085
S41
PtrMYB103
SpuMYB060
GSVIVT01033670001
AtMYB27
BnaMYB73
BnaMYB207
MdoMYB132
MdoMYB133
ZmMYB091
ZmMYB101
ZmMYB015
ZmMYB042
ZmMYB076
BnaMYB97
BnaMYB220
AtMYB59
BnaMYB143
BnaMYB29
AtMYB48
MdoMYB29
MdoMYB125
GSVIVT01025034001
SpuMYB149
SpuMYB150
PtrMYB206
PtrMYB023
SpuMYB086
SpuMYB088
S42

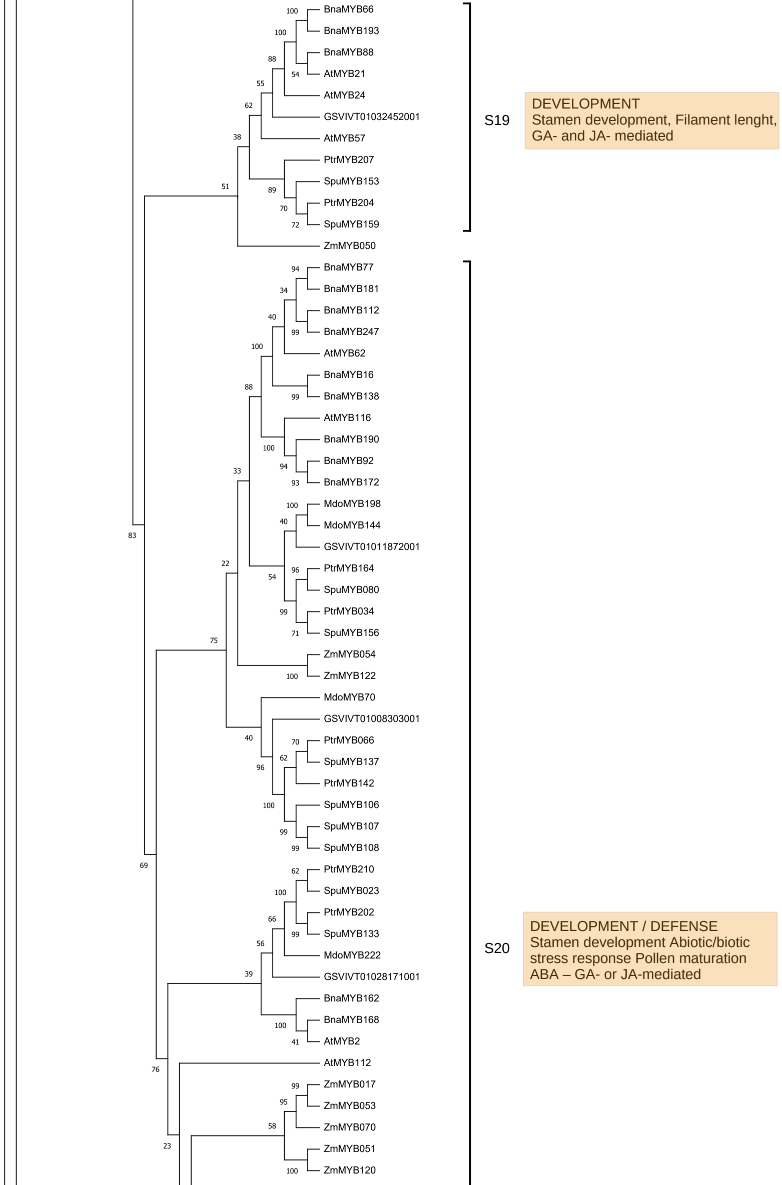
BnaMYB66
BnaMYB193
BnaMYB88
AtMYB21
AtMYB24
GSVIVT01032452001
AtMYB57
PtrMYB207
SpuMYB153
PtrMYB204
SpuMYB159
ZmMYB050
S19
DEVELOPMENT
Stamen development, Filament lenght,
GA- and JA- mediated
BnaMYB77
BnaMYB181
BnaMYB112
BnaMYB247
AtMYB62
BnaMYB16
BnaMYB138
AtMYB116
BnaMYB190
BnaMYB92
BnaMYB172
MdoMYB198
MdoMYB144
GSVIVT01011872001
PtrMYB164
SpuMYB080
PtrMYB034
SpuMYB156
ZmMYB054
ZmMYB122
MdoMYB70
GSVIVT01008303001
PtrMYB066
SpuMYB137
PtrMYB142
SpuMYB106
SpuMYB107
SpuMYB108
PtrMYB210
SpuMYB023
PtrMYB202
SpuMYB133
MdoMYB222
GSVIVT01028171001
BnaMYB162
BnaMYB168
AtMYB2
AtMYB112
ZmMYB017
ZmMYB053
ZmMYB070
ZmMYB051
ZmMYB120
S20
DEVELOPMENT / DEFENSE
Stamen development Abiotic/biotic
stress response Pollen maturation
ABA – GA- or JA-mediated

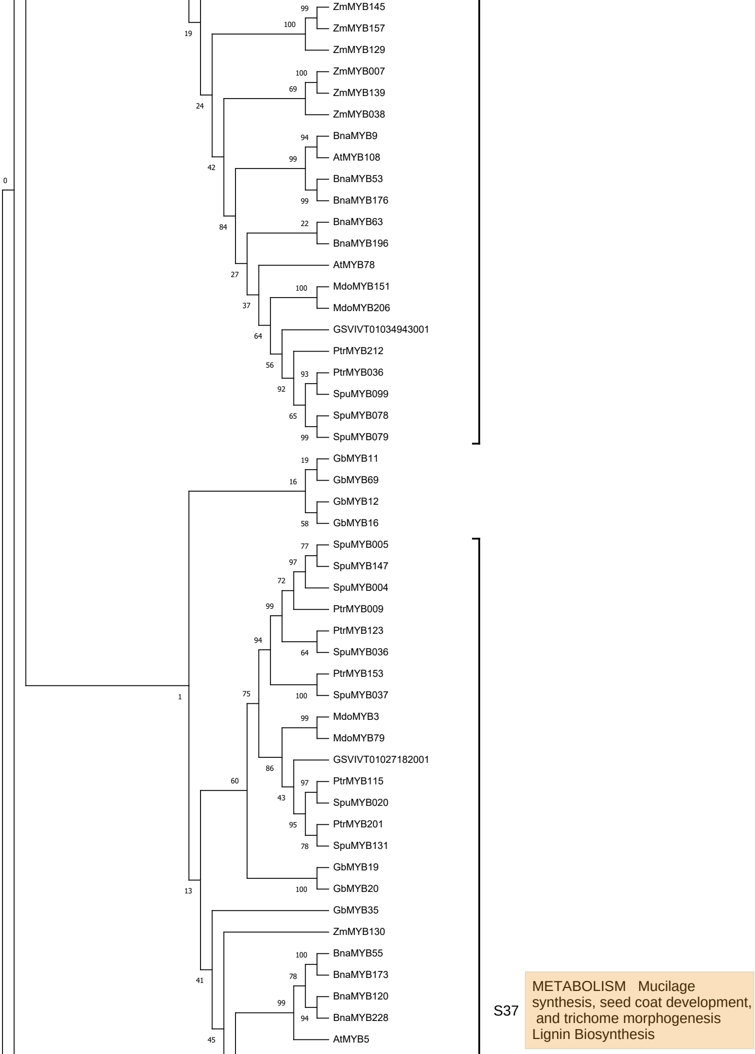
ZmMYB145
ZmMYB157
ZmMYB129
ZmMYB007
ZmMYB139
ZmMYB038
BnaMYB9
AtMYB108
BnaMYB53
BnaMYB176
BnaMYB63
BnaMYB196
AtMYB78
MdoMYB151
MdoMYB206
GSVIVT01034943001
PtrMYB212
PtrMYB036
SpuMYB099
SpuMYB078
SpuMYB079
GbMYB11
GbMYB69
GbMYB12
GbMYB16
SpuMYB005
SpuMYB147
SpuMYB004
PtrMYB009
PtrMYB123
SpuMYB036
PtrMYB153
SpuMYB037
MdoMYB3
MdoMYB79
GSVIVT01027182001
PtrMYB115
SpuMYB020
PtrMYB201
SpuMYB131
GbMYB19
GbMYB20
GbMYB35
ZmMYB130
BnaMYB55
BnaMYB173
BnaMYB120
BnaMYB228
AtMYB5
S37
METABOLISM Mucilage synthesis, seed coat development, and trichome morphogenesis
Lignin Biosynthesis

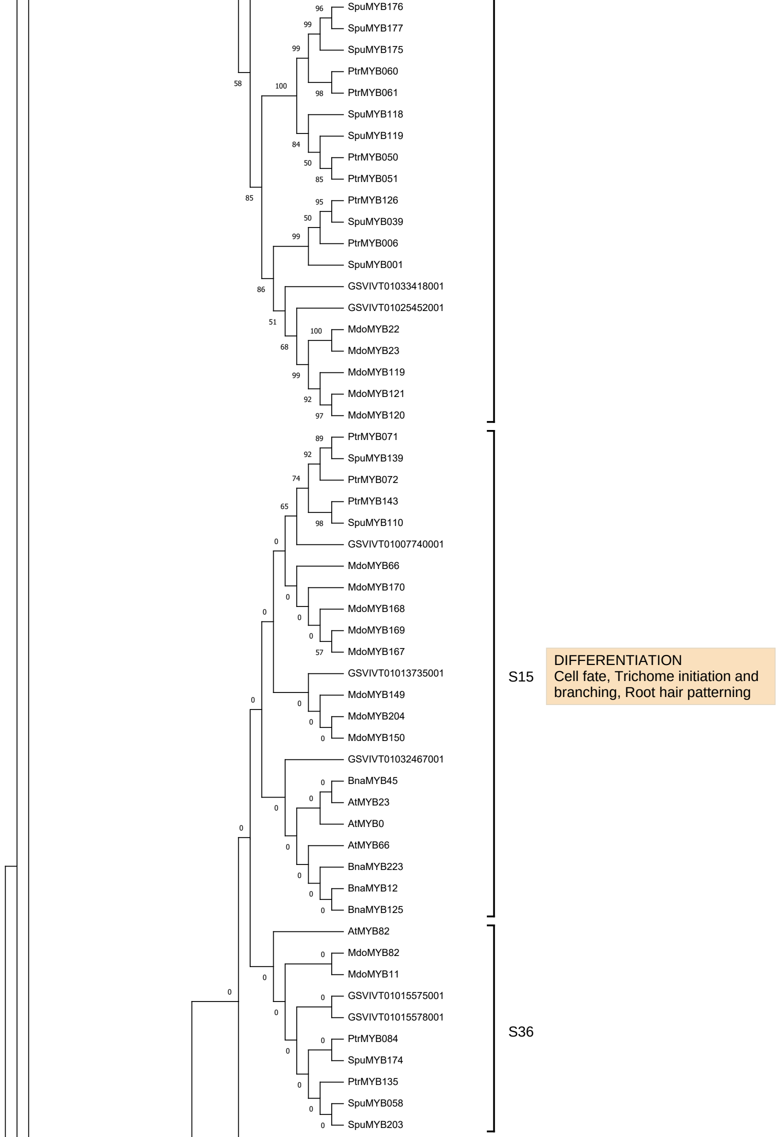

SpuMYB176
SpuMYB177
SpuMYB175
PtrMYB060
PtrMYB061
SpuMYB118
SpuMYB119
PtrMYB050
PtrMYB051
PtrMYB126
SpuMYB039
PtrMYB006
SpuMYB001
GSVIVT01033418001
GSVIVT01025452001
MdoMYB22
MdoMYB23
MdoMYB119
MdoMYB121
MdoMYB120
PtrMYB071
SpuMYB139
PtrMYB072
PtrMYB143
SpuMYB110
GSVIVT01007740001
MdoMYB66
MdoMYB170
MdoMYB168
MdoMYB169
MdoMYB167
GSVIVT01013735001
MdoMYB149
MdoMYB204
MdoMYB150
GSVIVT01032467001
BnaMYB45
AtMYB23
AtMYB0
AtMYB66
BnaMYB223
BnaMYB12
BnaMYB125
AtMYB82
MdoMYB82
MdoMYB11
GSVIVT01015575001
GSVIVT01015578001
PtrMYB084
SpuMYB174
PtrMYB135
SpuMYB058
SpuMYB203
S15
DIFFERENTIATION
Cell fate, Trichome initiation and branching, Root hair patterning
S36

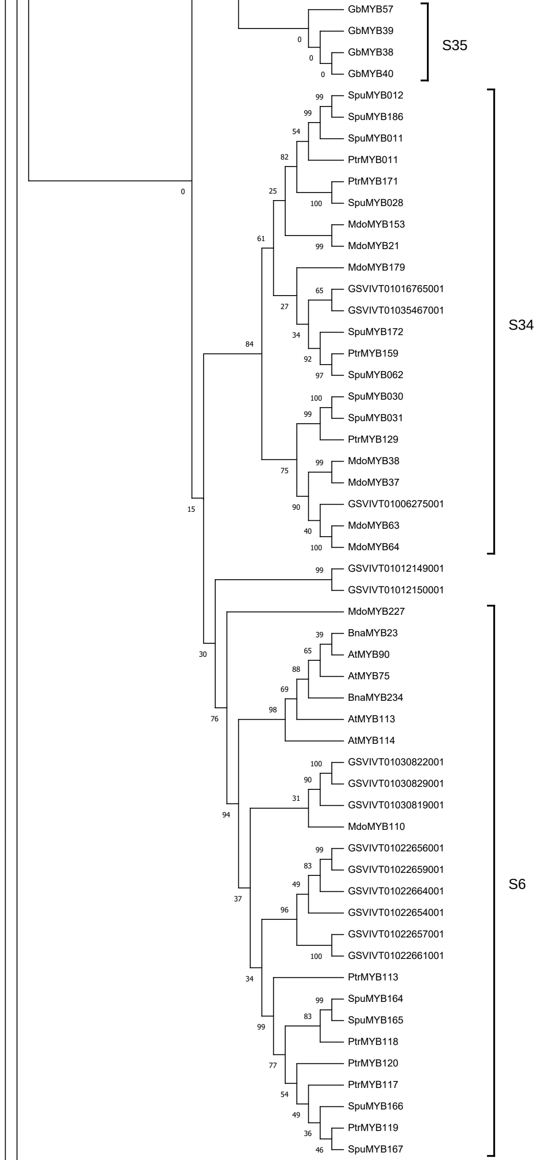
GbMYB57
GbMYB39
GbMYB38
GbMYB40
S35
SpuMYB012
SpuMYB186
SpuMYB011
PtrMYB011
PtrMYB171
SpuMYB028
MdoMYB153
MdoMYB21
MdoMYB179
GSVIVT01016765001
GSVIVT01035467001
SpuMYB172
PtrMYB159
SpuMYB062
SpuMYB030
SpuMYB031
PtrMYB129
MdoMYB38
MdoMYB37
GSVIVT01006275001
MdoMYB63
MdoMYB64
S34
GSVIVT01012149001
GSVIVT01012150001
MdoMYB227
BnaMYB23
AtMYB90
AtMYB75
BnaMYB234
AtMYB113
AtMYB114
GSVIVT01030822001
GSVIVT01030829001
GSVIVT01030819001
MdoMYB110
GSVIVT01022656001
GSVIVT01022659001
GSVIVT01022664001
GSVIVT01022654001
GSVIVT01022657001
GSVIVT01022661001
PtrMYB113
SpuMYB164
SpuMYB165
PtrMYB118
PtrMYB120
PtrMYB117
SpuMYB166
PtrMYB119
SpuMYB167
S6

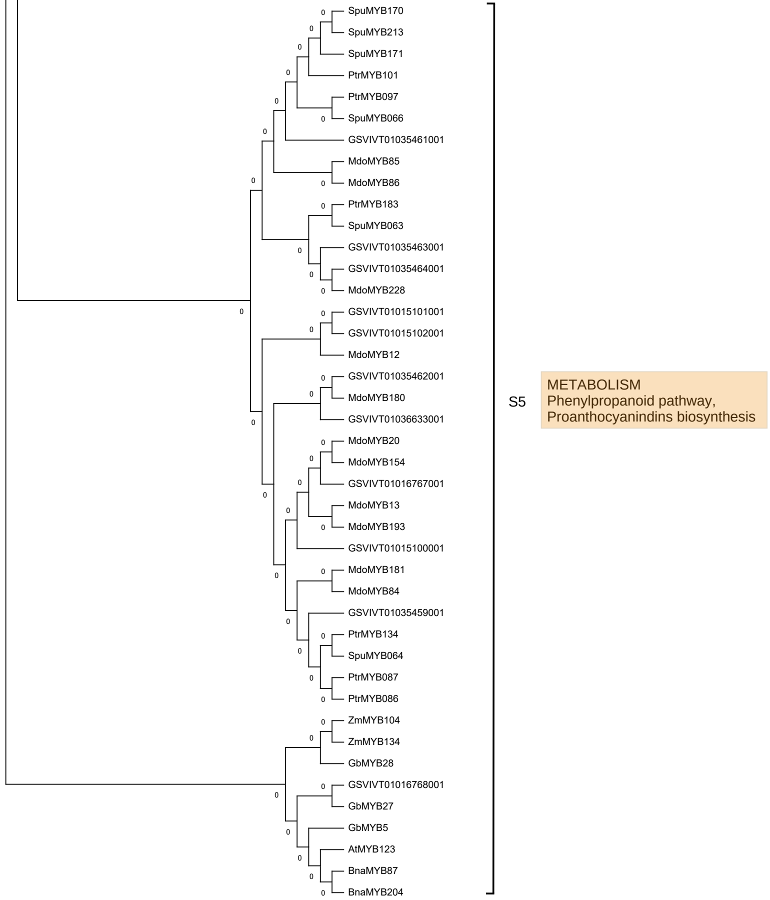
SpuMYB170
SpuMYB213
SpuMYB171
PtrMYB101
PtrMYB097
SpuMYB066
GSVIVT01035461001
MdoMYB85
MdoMYB86
PtrMYB183
SpuMYB063
GSVIVT01035463001
GSVIVT01035464001
MdoMYB228
GSVIVT01015101001
GSVIVT01015102001
MdoMYB12
GSVIVT01035462001
MdoMYB180
GSVIVT01036633001
MdoMYB20
MdoMYB154
GSVIVT01016767001
MdoMYB13
MdoMYB193
GSVIVT01015100001
MdoMYB181
MdoMYB84
GSVIVT01035459001
PtrMYB134
SpuMYB064
PtrMYB087
PtrMYB086
ZmMYB104
ZmMYB134
GbMYB28
GSVIVT01016768001
GbMYB27
GbMYB5
AtMYB123
BnaMYB87
BnaMYB204
S5
METABOLISM
Phenylpropanoid pathway,
Proanthocyanindins biosynthesis
